# Highly Efficient Kinetic Resolution of Aryl-Alkenyl Alcohols by Ru-Catalyzed Hydrogen Transfer

**DOI:** 10.3390/molecules26247475

**Published:** 2021-12-10

**Authors:** Yipeng You, Ming Yu Jin, Guanyu Tao, Xiangyou Xing

**Affiliations:** Shenzhen Grubbs Institute and Department of Chemistry, Guangdong Key Laboratory of Catalysis, Southern University of Science and Technology, Shenzhen 518055, China; 12031087@mail.sustech.edu.cn (Y.Y.); jinmy@sustech.edu.cn (M.Y.J.); taogy@mail.sustech.edu.cn (G.T.)

**Keywords:** asymmetric transfer hydrogenation, kinetic resolution, aryl-alkenyl alcohols, selectivity factor, Ru-catalyst

## Abstract

No matter through asymmetric reduction of ketones or kinetic resolution of secondary alcohols, enantioselective synthesis of the corresponding secondary alcohols is challenging when the two groups attached to the prochiral or chiral centers are spatially or electronically similar. For examples, dialkyl (sp^3^ vs. sp^3^), diaryl (sp^2^ vs. sp^2^), and aryl-alkenyl (sp^2^ vs. sp^2^) alcohols are difficult to produce with high enantioselectivities. By exploiting our recently developed Ru-catalysts of minimal stereogenicity, we reported herein a highly efficient kinetic resolution of aryl-alkenyl alcohols through hydrogen transfer. This method enabled such versatile chiral building blocks for organic synthesis as allylic alcohols, to be readily accessed with excellent enantiomeric excesses at practically useful conversions.

## 1. Introduction

**Figure 1:**
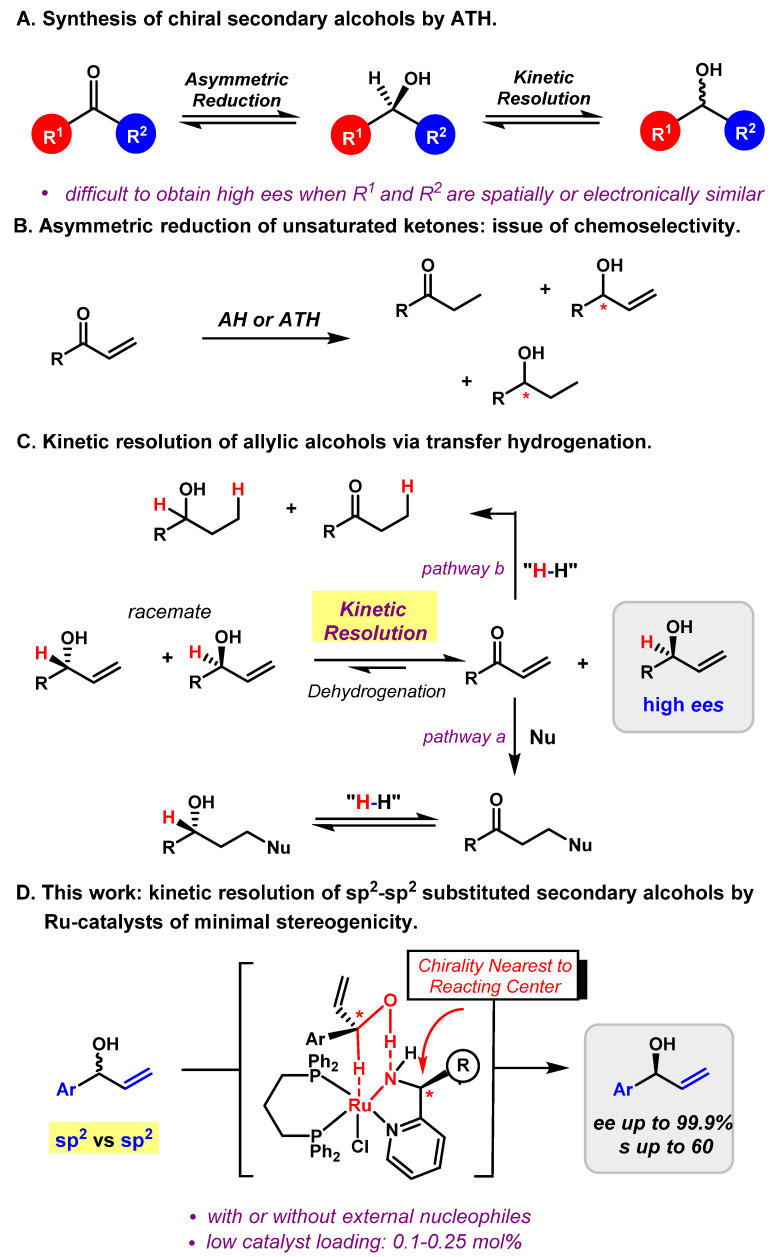
Kinetic resolution of racemic aryl-alkenyl alcohols via a Ru-catalyzed hydrogen transfer.

The Noyori asymmetric hydrogenation (AH) and asymmetric transfer hydrogenation (ATH) of carbonyl compounds are the most practical and atom-economical approaches for producing optically enriched secondary alcohol products, and have found widespread applications in both academic and industrial settings [1,2,3]. Asymmetric transfer hydrogenation, from the viewpoint of its mechanism, has two ways to generate enantiomerically enriched secondary alcohols, either by asymmetric reduction of the corresponding ketones [4,5,6,7,8,9] or by kinetic resolution of racemic secondary alcohols (Figure 1A) [10,11,12,13]. However, no matter through asymmetric reduction or kinetic resolution, it is challenging to achieve enantioselectivites of the alcohol products when the two groups attached to the prochiral or chiral centers are spatially or electronically similar [14]. For examples, due to the obstacles for chiral catalysts to differentiate two planar sp^2^ hybridized groups, aryl-alkenyl (sp^2^ vs. sp^2^) alcohols are difficult to produce with high enantioselectivities [15,16].

Secondary chiral allylic alcohols are an important and highly versatile class of building blocks for organic synthesis. However, direct asymmetric reductions of α,β-unsaturated ketones by either AH or ATH lead to a mixture of conjugate reduction, 1,2-reduction, and fully reduced products (Figure 1B) [16,17,18,19,20,21,22,23,24,25,26,27]. As such, kinetic resolution serves as an appealing approach for accessing the enantio-enriched secondary allylic alcohols, considering that the corresponding racemates are readily available and inexpensive [28,29,30,31,32,33,34,35,36,37,38,39,40]. Due to the reversibility of the dehydrogenation-hydrogenation process, kinetic resolution of secondary alcohols via transfer hydrogenation is highly dependent on the redox properties of the alcohols formed as well as the chiral recognition capabilities of the catalyst, and still remains a challenging task [10,11,12,13,14]. We hypothesized that the reversibility issue in kinetic resolution could be solved by the following two ways. Firstly, adding an external nucleophile, such as an amine or an alcohol, would drive the reversible dehydrogenation of one reactive enantiomer to the corresponding α,β-unsaturated ketone that would then undergo a subsequent conjugate addition (Figure 1C, pathway a). Secondly, the hydride abstracted from one enantiomer could be transferred back to the dehydrogenated intermediate (α,β-unsaturated ketone) by conjugate reduction, thus driving the equilibrium forward (Figure 1C, pathway b). By either way, the less reactive enantiomer would be recovered with high enantiomeric excesses (ees).

According to the excellent performances of our recently developed Ru-catalysts of minimal stereogenicity (i.e., merely a single chirality element) in transfer hydrogenations [41,42,43,44], we envisioned that kinetic resolution of the aryl-alkenyl (sp^2^ vs. sp^2^) secondary alcohols (aryl substituted allylic alcohols) could be realized via the Ru-catalyzed hydrogen transfer, with or without an external nucleophile. Herein, a highly efficient kinetic resolution of aryl-alkenyl alcohols is described (Figure 1D). This reaction features a low catalyst loading (0.1–0.25 mol%) and a broad substrate scope with high enantioselectivities (ee up to 99.9%). To our knowledge, the kinetic resolution of secondary allylic alcohols through transfer hydrogenation has been rarely reported [10,11,12,13,14].

## 2. Results and Discussion

### 2.1. Optimization of the Reaction Conditions

**Scheme 1:**
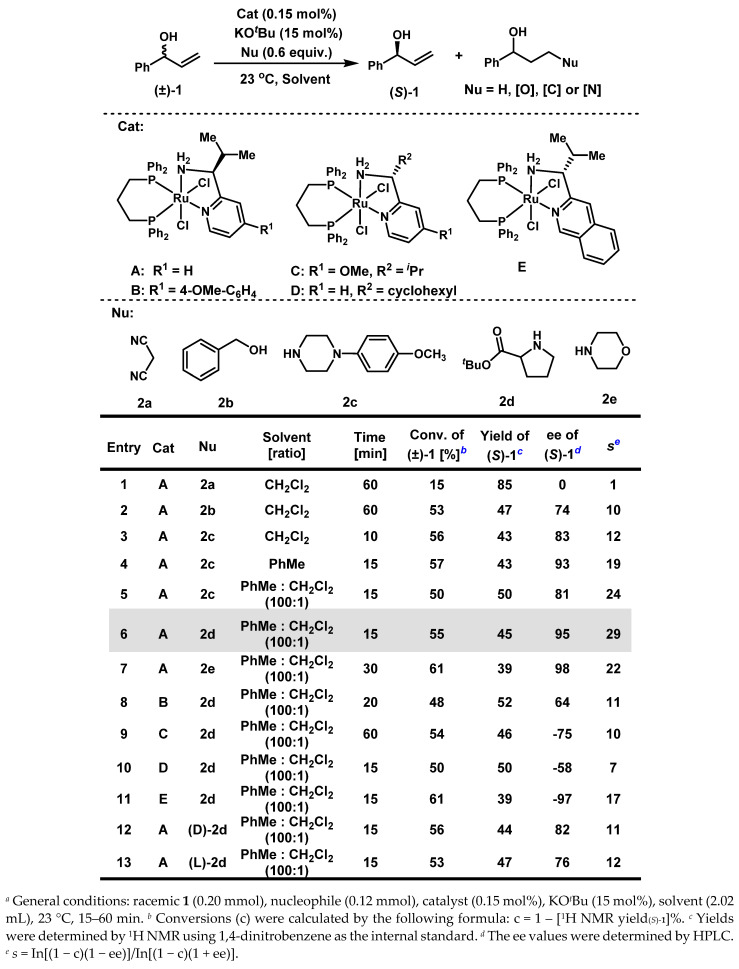
Optimization of Conditions *^a^*.

Our studies began with kinetic resolution of (±)-1-phenylallyl alcohol **1** by employing 0.15 mol% of Ru-catalyst **A** (Scheme 1). Ideally, racemic **1** could be kinetically resolved with one enantiomer recovered in high enantioselectivity at around 50% conversion, and the other enantiomer undergoing a cascade reaction of dehydrogenation/1,4-reduction or conjugate addition/1,2- reduction to afford fully reduced alcohols or γ-functionalized alcohols. Both selectivity factors (*s*) and ees of resolved substrates are diagnostic indicators for evaluating the kinetic resolution process [45]. The effects of nucleophiles were initially investigated. When the carbon-based nucleophile, malononitrile **2a**, was added, **1** was recovered with no enantioselectivity at 15% conversion (Scheme 1, entry 1). In contrast, when an oxygen-based nucleophile, such as benzyl alcohol **2b,** was added, a successful kinetic resolution was obtained, with **1** being resolved in 74% ee at 53% conversion in 60 min (Scheme 1, entry 2). A more efficient kinetic resolution was realized when a nitrogen-based nucleophile, such as 1-(4-methoxyphenyl)piperazine **2c**, was added: **1** was resolved in 83% ee at 56% conversion within 10 min (Scheme 1, entry 3). When CH_2_Cl_2_ was replaced by toluene, both the enantioselectivity and the selectivity factor of **1** were further improved (Scheme 1, entry 4). We noticed that catalyst **A** did not dissolve very well in toluene; therefore, a tiny amount of CH_2_Cl_2_ was used as co-solvent in combination with toluene to increase the solubility of the catalyst. A 50% yield and 81% ee of recovered **1** was then recorded at 50% conversion, resulting in an increase in the selectivity factor to 24 (Scheme 1, entry 5). Other amine-based nucleophiles, including proline *t*-butyl ester **2d** and morpholine **2e**, were then surveyed, and both provided excellent enantioselectivities and selectivity factors, respectively (Scheme 1, entry 6 and 7). We further explored other Ru-catalysts of minimal stereogenicity [41,42,43,44]. Catalysts **B** and **C**, both with R^1^ as the electron-donating group (Scheme 1, entry 8: R^1^ = 4-OMe-C_6_H_4_ for **B**; Scheme 1, entry 9: R^1^ = OMe for **C**), produced decreased enantioselectivities of **1** at around 50% conversion, but still affording successful kinetic resolutions. When R^2^ is a cyclohexyl group in catalyst **D** (Scheme 1, entry 10), only 58% ee of recovered **1** at 50% conversion was observed, leading to an unsuccessful kinetic resolution (*s* = 7). Gratifyingly, when the pyridyl group was replaced by a more conjugated aryl ring, such as isoquinoline in catalyst **E**, comparable results were obtained as with catalyst **A**: a selectivity factor of 17 and 97% ee for **1** were observed (Scheme 1, entry 11). Finally, the enantiopure (l) and (d)-proline *t*-butyl esters were tested, and the selectivity factors of 11 and 12 were obtained, respectively (Scheme 1, entry 12 and entry 13).

### 2.2. Substrate Scope for the Kinetic Resolution of Aryl-Alkenyl Alcohols

**Figure 2:**
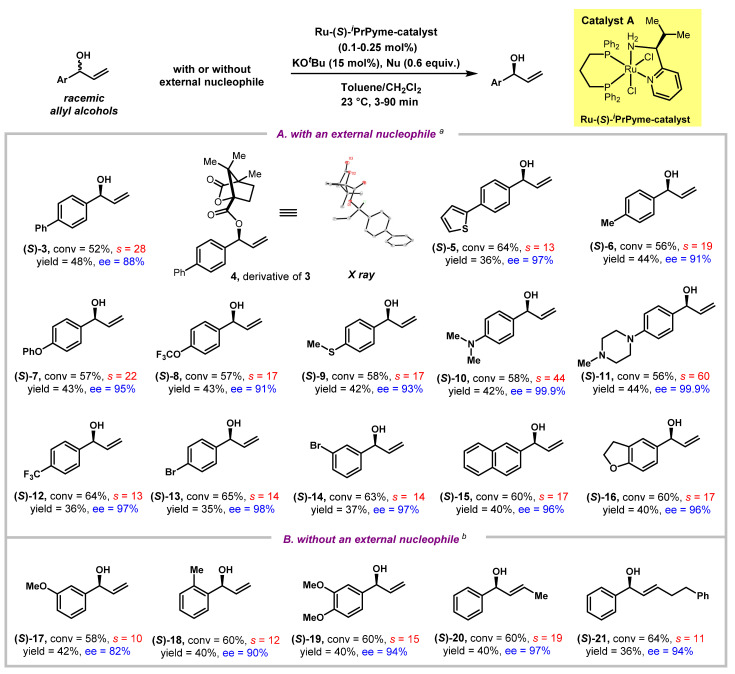
Kinetic resolution of allylic alcohols via hydrogen transfer. General conditions: racemic allylic alcohol (0.2 mmol), Ru-(*S*)-*^i^*PrPyme-catalyst (0.1–0.25 mol%), KO*^t^*Bu (15 mol%), toluene or CH_2_Cl_2_ (2.0 mL), 23 °C. *^a^*An amine nucleophile (**2d** or **2e,** 0.12 mmol) was added. *^b^* No external nucleophile was added. Conversion (c) was calculated by the following formula: c = 1− (^1^H NMR yield_(recovered alcohols)_)%. Yields were determined by ^1^H NMR using 1,4-dinitrobenzene as the internal standard. *s* = In[(1 − c)(1 − ee)]/In[(1 − c)(1 + ee)].

With the optimized condition in hand, we then explored the substrate scope for the kinetic resolution protocol. As shown in Figure 2A, under the catalysis of Ru-catalyst **A** that was used with as low as 0.1–0.25 mol% loading, together with 0.6 equivalent of an amine nucleophile (such as proline *t*-butyl ester **2d** or morpholine **2e**), a variety of racemic allylic alcohols were kinetically resolved with good to excellent ees (82% to 99.9%) at practically useful conversions. Although sometimes kinetic resolution was considered to be poor “atom-economy” due to a maximum yield of 50% based on racemic substrates, the ees obtained in this process could be extremely high at the expense of higher reaction conversions [45,46]. Racemate **3** with *para*-substitution on the aryl ring by a phenyl group underwent catalytic kinetic resolution with a selectivity factor of 28, yielding the recovered substrate **3** in 88% ee at 52% conversion. We were able to grow a single crystal of compound **4,** that was prepared by derivatizing the recovered **3** with the chiral auxiliary (1*S*)-camphanic chloride (see the Supplementary Materials), establishing unambiguously that the less reactive enantiomer of racemate **3** that was recovered had the (*S*)-configuration at its allylic carbon. Then, an array of aryl allyl alcohols were investigated. When the *para*-substitution on the aryl rings varied among thiophene (**5**), methyl (**6**), phenoxy (**7**), trifluoromethoxy (**8**), methylthio (**9**), dimethylamino (**10**), *N*-methylpiperazine (**11**), trifluoromethyl (**12**), and bromide (**13**), being either electron-donating or withdrawing in nature, the selectivity factors were in the range of 13–60, and excellent enantioselectivities of resolved alcohols were obtained in all the cases (**5**–**13**, 91–99.9% ees). Other substitution patterns on the aryl ring, such as *meta*-substituted bromide, were also tolerated, leading to 97% ees in the corresponding resolved alcohol **14** at the 63% conversion. Kinetic resolutions of naphthalenyl and dihydrobenzofuran substituted allyl alcohols were also investigated, and good results were obtained in these cases (**15**, 96% ee, *s* = 17; **16**, 96% ee, *s* = 17).

Additionally, we discovered that besides resolved allylic alcohols, β-functionalized ketones, and γ-functionalized alcohols, fully reduced alcohols deriving from both 1,4- and 1,2-reduction by the Ru-H were also observed in crude ^1^H NMR, hinting on the possibility of conducting the kinetic resolution without adding any external nucleophile as a driving force (Figure 1C, pathway b). Therefore, kinetic resolution of allylic alcohols via hydrogen transfer without any external nucleophiles were then explored (Figure 2B). The racemic substrates, not only with mono-substituted alkenes (**17**–**19**), but also with (*E*)-disubstituted alkenes (**20**–**21**), were all successfully resolved in 82% to 97% ees, with selectivity factors ranging from 10 to 19.

## 3. Materials and Methods

### 3.1. General Information

All reactions were carried out under an argon atmosphere with dry solvents under anhydrous conditions, unless otherwise noted. The catalysts were synthesized following the procedure outlined by our group recently [41,42,43,44]. Anhydrous toluene and THF were distilled from sodium-benzophenone. Dichloromethane and trimethylamine were distilled from calcium hydride. Flash column chromatography was performed using Tsingtao silica gel (60, particle size 0.040–0.063 mm). Reagents were purchased at the highest commercial quality and used without further purification, unless otherwise stated. ^1^H NMR (400 MHz and 600 MHz), ^13^C NMR (101 MHz and 151 MHz), and ^19^F NMR (565 MHz and 376 MHz) spectra were recorded on a Bruker AV III HD spectrometer (Romanshorn, Switzerland), and reported in terms of chemical shift relative to residual CDCl_3_ (δ 7.26 and δ 77.0 ppm, respectively). High-resolution mass spectra (HRMS) data were obtained by using Thermo Scientific™ Q Exactive™ Quadrupole-Orbitrap Mass Spectrometer (Midland, Canada). HPLC (High Performance Liquid Chromatography) analysis was conducted on Agilent 1260 instrument (Waldbronn, Germany) and Shimadzu LC-20A instrument (Kyoto, Japan) using chiral column described below in detail. Specific optical rotation was measured on a Rudolph-Autopol I (Hackettstown, NJ, USA).

### 3.2. General Experimental Procedure for the Kinetic Resolution with or without an External Nucleophile

In an argon filled glovebox, an oven-dried resealable tube equipped with a magnetic stir bar was charged with the Ru-catalyst (0.1–0.25 mol%), racemic allylic alcohol (0.2 mmol), an amine nucleophile (0.12 mmol) or without an external nucleophile, toluene (2.0 mL), and dichloromethane (20 μL). *t*-BuOK (30 μL, 1.0 M in *t*-BuOH) was added into the above reaction mixture. After reaching a 45% to 65% conversion, the reaction was quenched by H_2_O and extracted with ethyl acetate (3 × 5 mL). The combined organic layers were dried over Na_2_SO_4_ and concentrated under reduced pressure. The 1,4-dinitrobenzene (8.4 mg, 0.05 mmol) was added into the crude residue as the internal standard to determine the crude ^1^H NMR yield of the recovered allylic alcohol. The reaction conversion (c) was calculated by the following formula: c = 1 − (^1^H NMR yield_(recovered alcohols)_)%. The enantiomeric excess (ee) of the recovered allylic alcohol was determined by HPLC analysis. The selectivity factor (*s*) of the kinetic resolution was thus calculated by the formula: *s* = In[(1 − c)(1 − ee)]/In[(1 − c)(1 + ee)].

## 4. Conclusions

In conclusion, by exploiting our recently developed Ru-catalysts, an example of kinetic resolution of various aryl-alkenyl secondary alcohols through transfer hydrogenation was reported with high efficiencies. Amine-based nucleophiles were found to serve as a strong driving force for the kinetic resolution process, so that the corresponding α,β-unsaturated ketones that derive from the dehydrogenation of one reactive enantiomer would readily undergo a conjugate addition. In addition, this kinetic resolution can also be realized without an external nucleophile, since the hydride abstracted from the racemic allylic alcohols can be added back to the resulting α,β-unsaturated ketones, thus driving the reaction forward. This discovery enables versatile aryl-alkenyl alcohols to be readily accessed, with excellent enantioselectivities at practically useful conversions. We believed that this work is a good application of the chiral Ru-catalysts in chiral recognition with high levels of efficiency. Research to realize further applications via the asymmetric transfer hydrogenation is under way.

## Data Availability

The data presented in this study are available.

## Supplementary Materials

The following are available online, in the Supplementary Materials: materials and methods, experimental procedures, characterization data, ^1^H^, 13^C, NMR spectra, HPLC chromatograms, mass spectrometry data, and X-ray crystallography data.

## Sample Availability

Samples of the compounds are available from the authors.
