# Supplementary material for: Highly Efficient Kinetic Resolution of Aryl-Alkenyl Alcohols by Ru-Catalyzed Hydrogen Transfer
Source: Molecules. 2021 Dec 10;26(24):7475. doi: 10.3390/molecules26247475 (PMC8705739; doi:10.3390/molecules26247475)
Supplement: Supplementary file 1 [file molecules-26-07475-s001.zip › molecules-1488483-supplementary.pdf]

Supporting Information

Highly Efficient Kinetic Resolution of Aryl-Alkenyl Alcohols by Ru-Catalyzed Hydrogen Transfer

Yipeng You†, Ming Yu Jin†, Guanyu Tao, and Xiangyou Xing*

Shenzhen Grubbs Insitute and Department of Chemistry, Guangdong Provincial Key Laboratory of Catalysis, Southern University of Science and Technology, Shenzhen, 518055, Guangdong, China

†*These authors contributed equally to this work.*

E-mail: xingxy@sustech.edu.cn

1. Materials and methods 3

2. The synthesis and characterization of racemic allylic alcohols 4

3. Optimization of conditions 5

4. Kinetic resolution of allylic alcohols via borrowing hydrogen cascade 17

5. X-Ray crystal data 53

6. References 58

7. NMR 59

1. Materials and methods

All reactions were carried out under an argon atmosphere with dry solvents under anhydrous conditions, unless otherwise noted. All the chemicals were purchased commercially, and used without further purification. The catalysts were synthesized following the procedure outlined by our group recently[1-4]. Anhydrous toluene and THF was distilled from sodium-benzophenone. Dichloromethane and trimethylamine were distilled from calcium hydride. Thin-layer chromatography (TLC) was conducted with 0.25 mm Tsingtao silica gel plates (60F-254) and visualized by exposure to UV light (254 nm). Flash column chromatography was performed using Tsingtao silica gel (60, particle size 0.040–0.063 mm). Reagents were purchased at the highest commercial quality and used without further purification, unless otherwise stated. ^{1}H NMR (400 MHz and 600 MHz), ^{13}C NMR (101 MHz and 151 MHz), ^{19}F NMR (565 MHz and 376 MHz) spectra were recorded on a Bruker AV III HD spectrometer, and were reported in terms of chemical shift relative to residual $CDCl_3$ (δ 7.26 and δ 77.0 ppm, respectively). Data for ^{1}H NMR spectra are reported as follows: chemical shift (δ ppm) (multiplicity, coupling constant (Hz), integration). Abbreviations are used as follows: s = singlet, br = broad singlet, d = doublet, t = triplet, q = quartet, m = complex multiplet. Data for ^{13}C NMR spectra are reported in terms of chemical shift. High-resolution mass spectra (HRMS) data was obtained by using Thermo Scientific™ Q Exactive™ Quadrupole-Orbitrap Mass Spectrometer. HPLC analysis was conducted on Agilent 1260 instrument and Shimadzu LC-20A instrument using chiral column described below in detail. Specific optical rotation was measured on a Rudolph-Autopol I.

2. The synthesis and characterization of racemic allylic alcohols

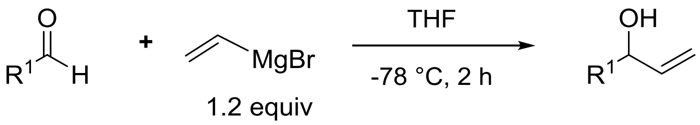

To a stirred solution of aldehyde (10.0 mmol) in dry THF (10 mL), Grignard reagent (12.0 mmol) in THF solution (1 M, 12 mL) was slowly added at -30 ^{o}C under nitrogen. Then the mixture was stirred at -30 ^{o}C for 1 h. The resulting solution was quenched with NH_4Cl aqueous solution (5 mL), and then extracted by ethyl acetate, dried over Na_2SO_4 and concentrated under reduce pressure. The crude product was purified by silica flash chromatography (eluted with hexane/EtOAc) to give racemic allylic alcohols. Compounds **(±)-1**, **3**, 5-**10**, **12-15**, **17**, **19**[2] and **(±)-18**[5] were known. Characterization of compounds **(±)-11** and **(±)-16** were described below in detail.

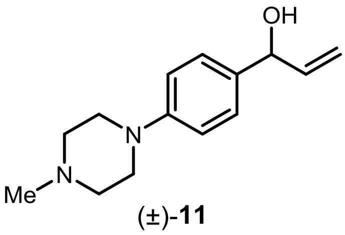

(±)-**11**

^{1}H NMR of (±)-11 (400 MHz, $CDCl_3$): δ 7.25 - 7.27 (m, 2H), 6.89 (d, *J* = 8.6 Hz, 2H), 6.00 - 6.09 (m, 1H), 5.30 - 5.35 (m, 1H), 5.12 - 5.18 (m, 2H), 3.14 - 3.17 (m, 4H), 2.54 - 2.56 (m, 4H), 2.33 (s, 3H) ppm.

^{13}C NMR of (±)-11 (101 MHz, $CDCl_3$): δ 150.9, 140.5, 133.9, 127.4, 116.0, 114.5, 74.9, 55.0, 48.9, 46.1 ppm.

HRMS (ESI) of (±)-11 m/z: $[M + H]^+$ Calcd for $C_{14}H_{21}N_2O$ 233.1648; Found 233.1649.

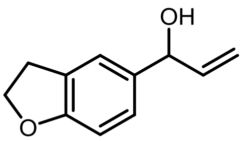

(±)-**16**

^{1}H NMR of (±)-16 (600 MHz, $CDCl_3$): δ 7.21 (s, 1H), 7.09 (d, *J* = 8.2 Hz, 1H), 6.74 (d, *J* = 8.1 Hz, 1H), 5.99 - 6.08 (m, 1H), 5.33 (d, *J* = 17.0 Hz, 1H), 5.17 (d, *J* = 10.4 Hz, 1H), 5.12 (d, *J* = 5.7 Hz, 1H), 4.56 (t, *J* = 8.7 Hz, 2H), 3.19 (t, *J* = 8.7 Hz, 2H), 2.12 (s, 1H) ppm.

^{13}C NMR of (±)-16 (151 MHz, $CDCl_3$): δ 159.7, 140.5, 140.5, 134.9, 127.3, 127.3, 126.5, 123.2, 114.5, 109.1, 75.1, 71.3, 29.6 ppm.

HRMS (ESI) of (±)-16 m/z: $[M - H]^-$ Calcd for $C_{11}H_{11}O_2$ 175.0754; Found 175.0756.

3. Optimization of conditions

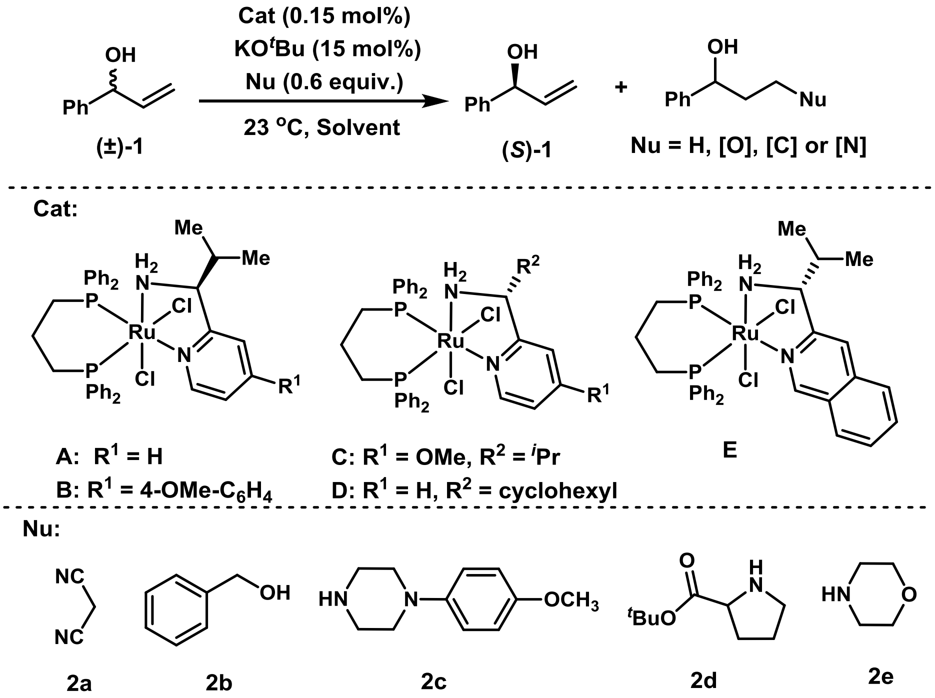

Entry	Cat	Nu	Solvent [ratio]	Time [min]	Conv. of (±)-1 [%][b]	Yield of (S)-1[c]	ee of (S)-1[d]	s[e]
1	A	2a	CH_2Cl_2	60	15	85	0	1
2	A	2b	CH_2Cl_2	60	53	47	74	10
3	A	2c	CH_2Cl_2	10	56	43	83	12
4	A	2c	PhMe	15	57	43	93	19
5	A	2c	PhMe : CH_2Cl_2 (100:1)	15	50	50	81	24
6	A	2d	PhMe : CH_2Cl_2 (100:1)	15	55	45	95	29
7	A	2e	PhMe : CH_2Cl_2 (100:1)	30	61	39	98	22
8	B	2d	PhMe : CH_2Cl_2 (100:1)	20	48	52	64	11
9	C	2d	PhMe : CH_2Cl_2 (100:1)	60	54	46	-75	10
10	D	2d	PhMe : CH_2Cl_2 (100:1)	15	50	50	-58	7
11	E	2d	PhMe : CH_2Cl_2 (100:1)	15	61	39	-97	17
12	A	(D)-2d	PhMe : CH_2Cl_2 (100:1)	15	56	44	82	11
13	A	(L)-2d	PhMe : CH_2Cl_2 (100:1)	15	53	47	76	12

[a] Reaction conditions: allylic alcohol **1** (0.2 mmol), nucleophile **2** (0.12 mmol), KO^{t}Bu (15 mol%), catalyst (0.15 mol%). [b] Conversion (c) was calculated by the following formula: c = 1- (^{1}H NMR yield$_{(s)-1}$)%. [c] Yields were determined by ^{1}H NMR using 1,4-dinitrobenzene as the internal standard. [d] The ee values were determined by HPLC. [e] s = In[(1-c)(1-ee)]/In[(1-c)(1+ee)].

Crude ^{1}H NMR of entry **1**

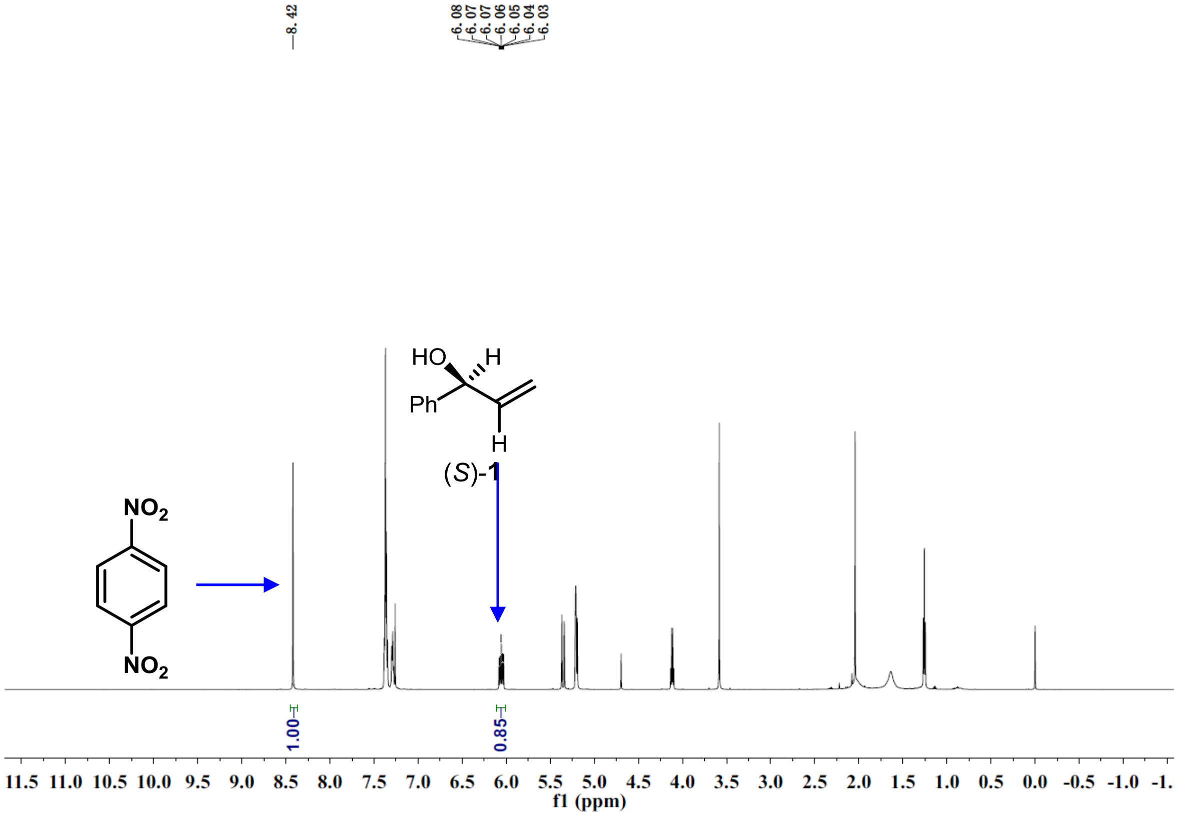

Crude ^{1}H NMR of entry **2**

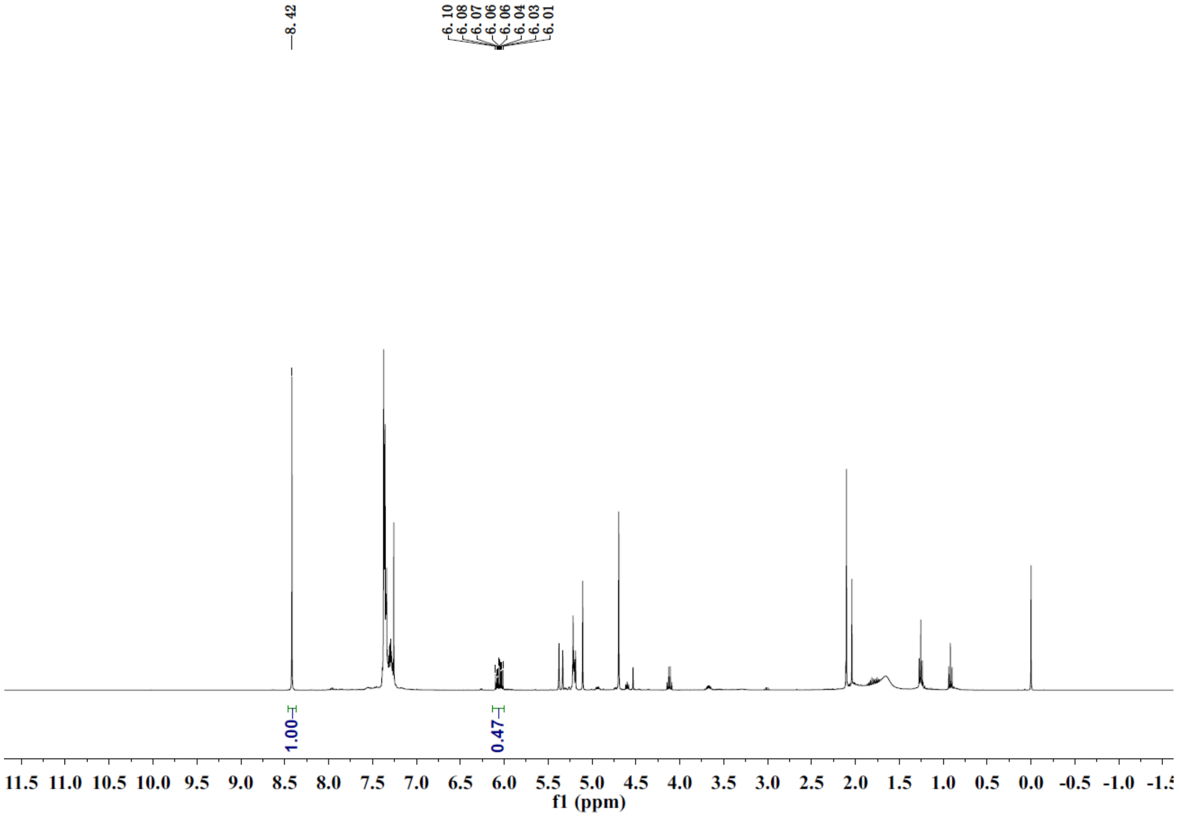

Crude ^{1}H NMR of entry **3**

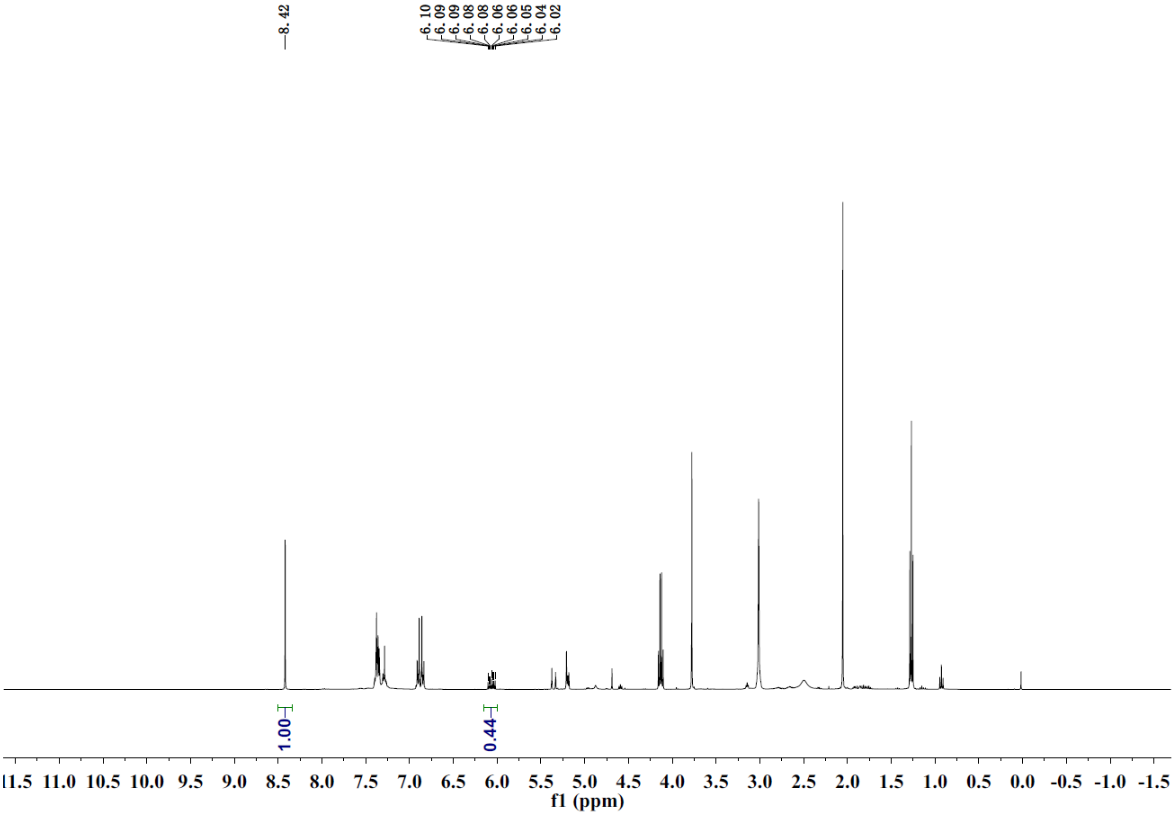

Crude ^{1}H NMR of entry **4**

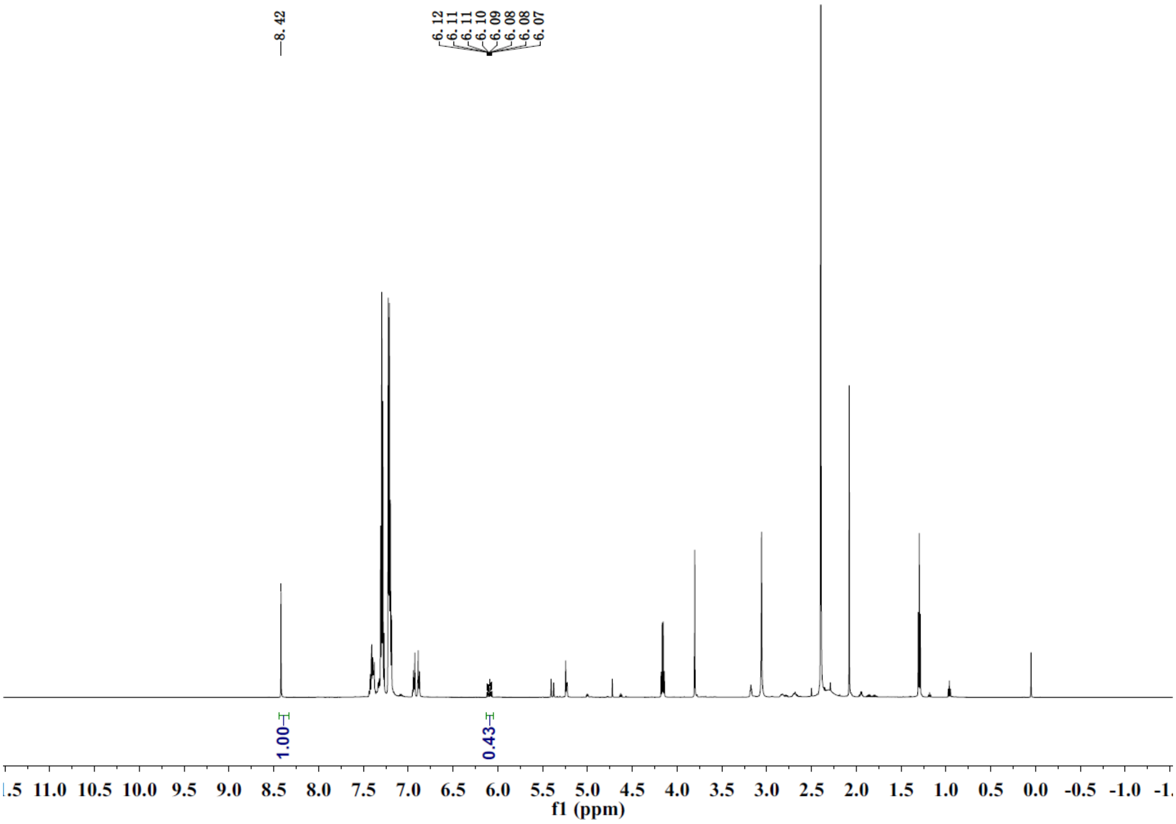

Crude ^{1}H NMR of entry **5**

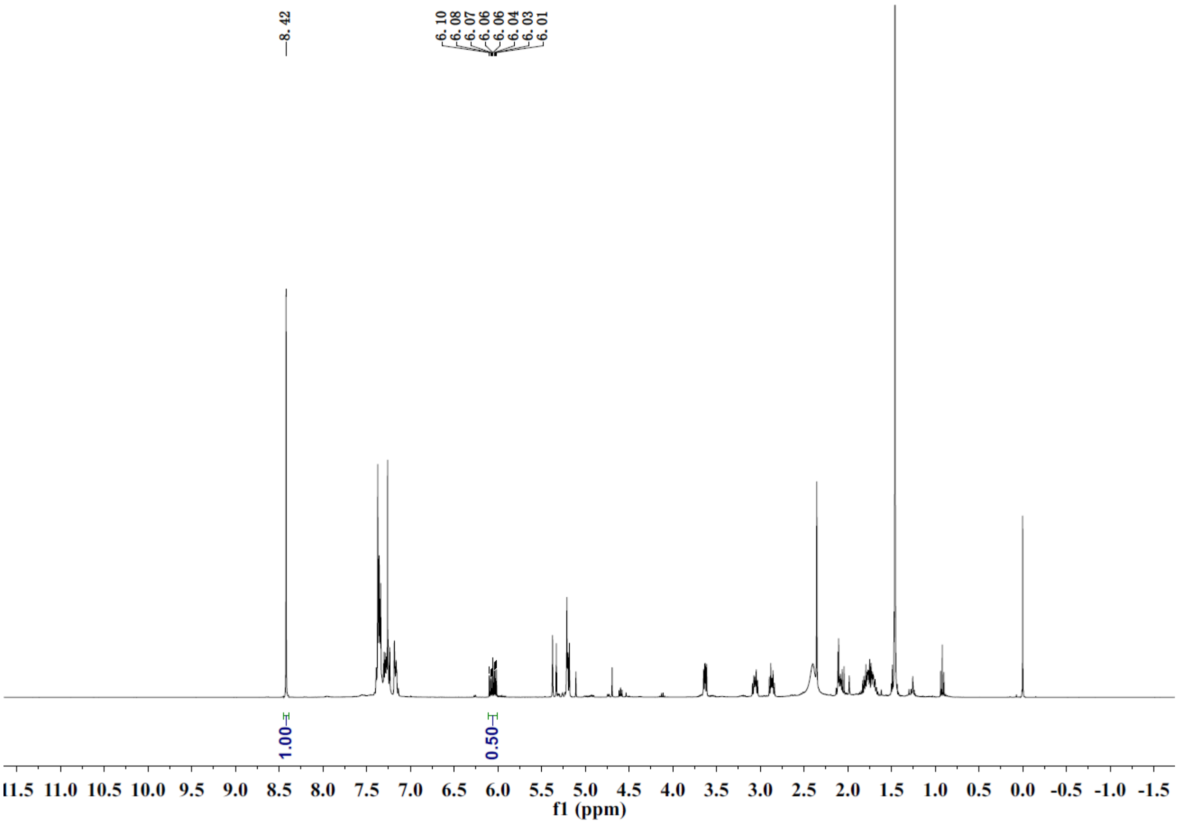

Crude ^{1}H NMR of entry **6**

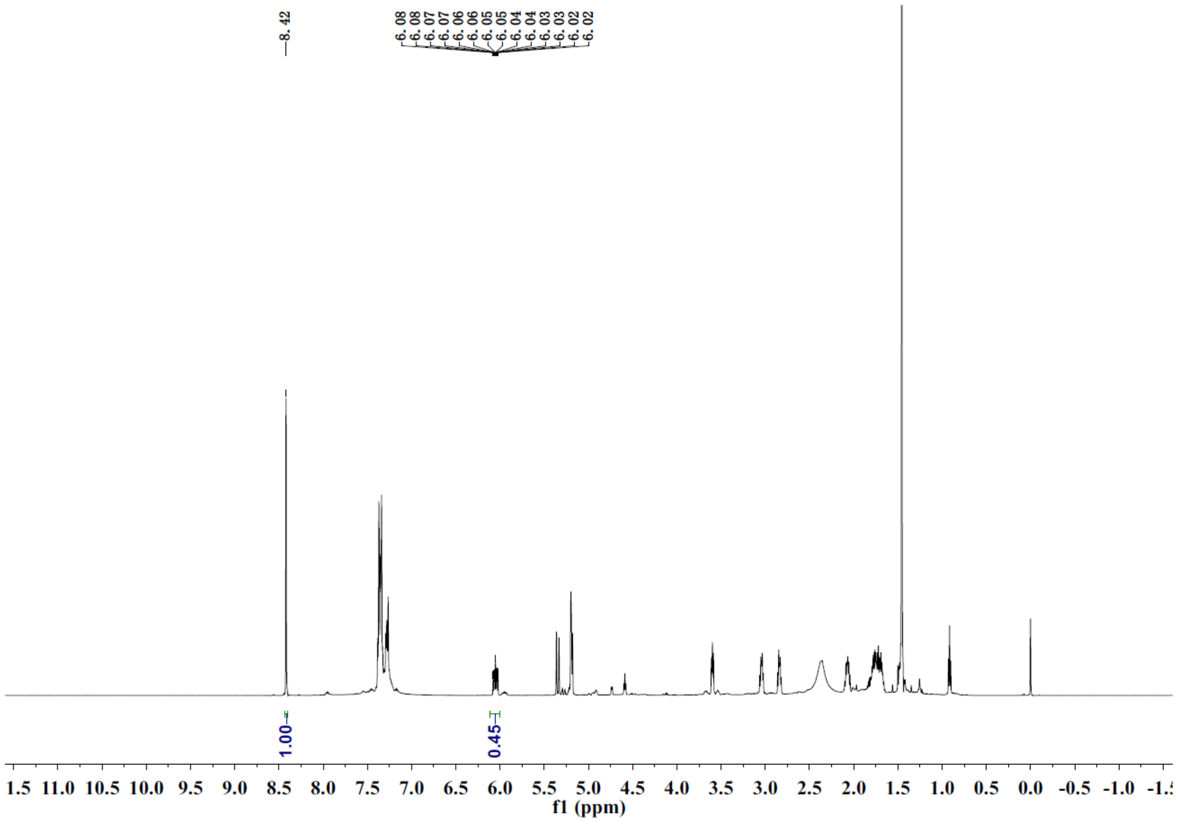

Crude ^{1}H NMR of entry **7**

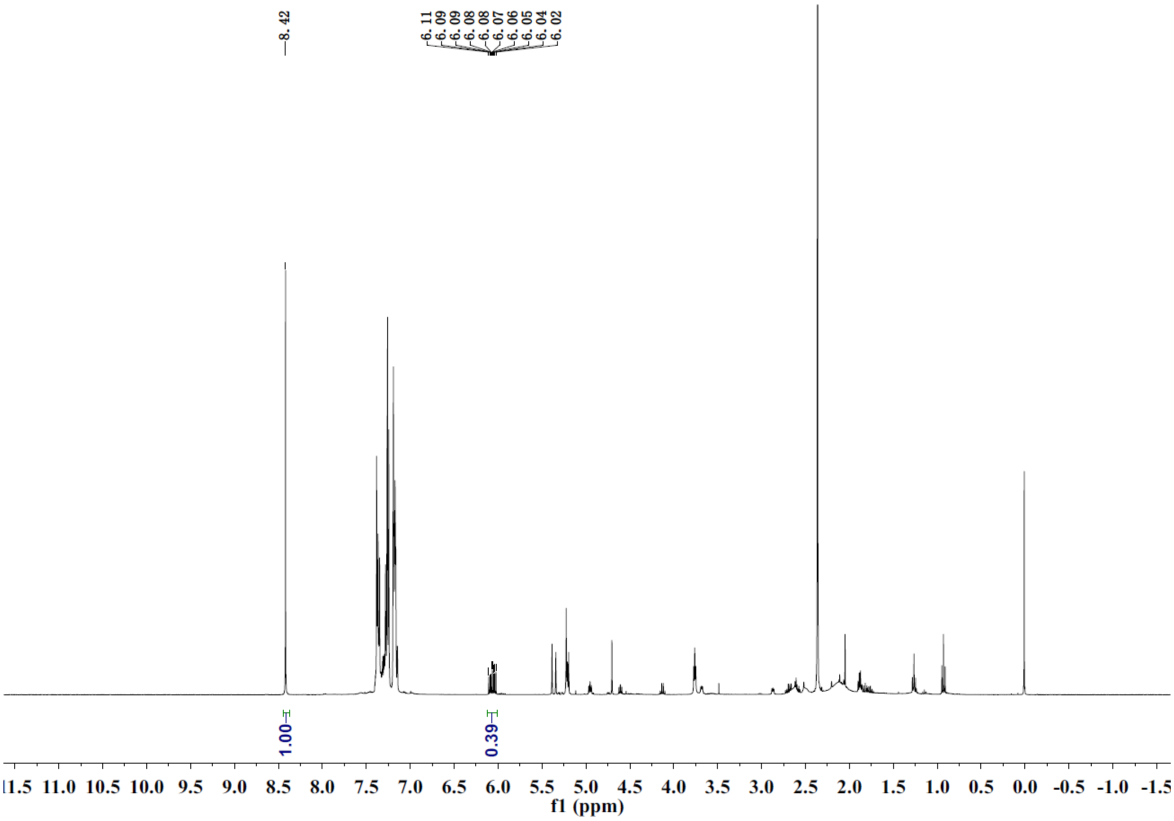

Crude ^{1}H NMR of entry **8**

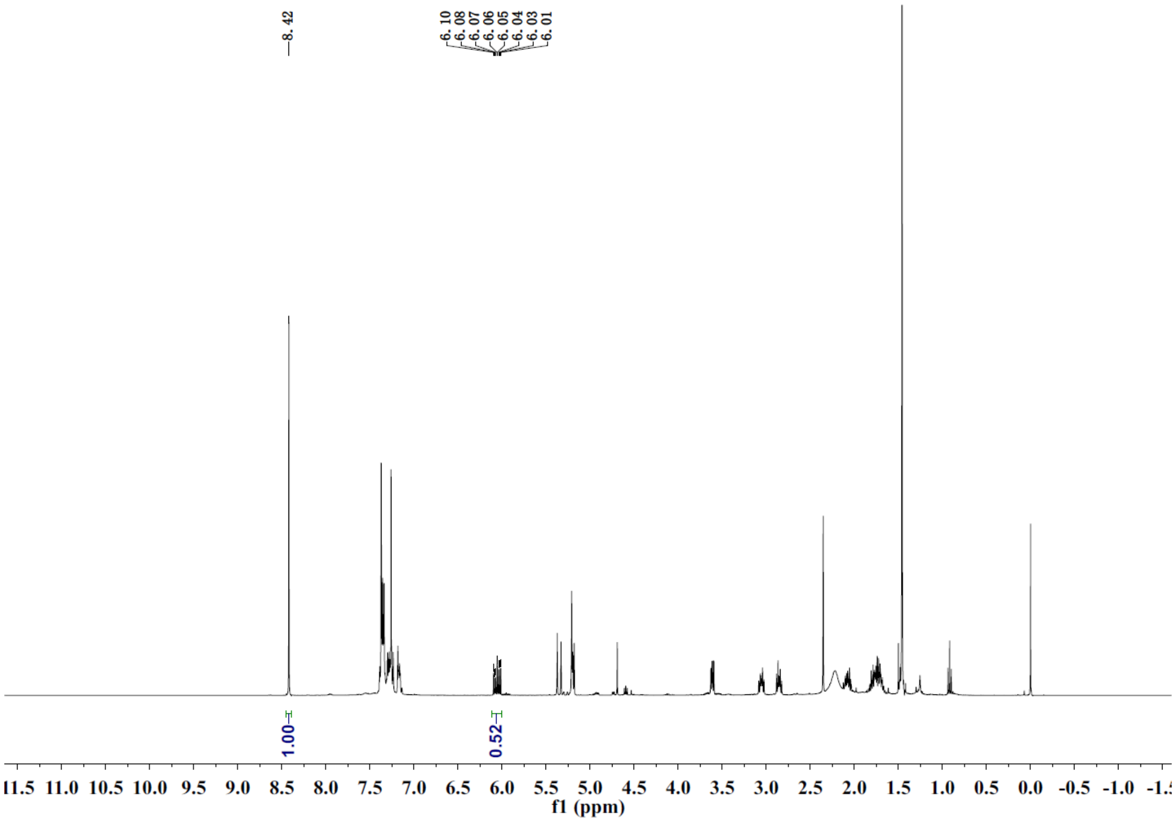

Crude ^1H NMR of entry **9**

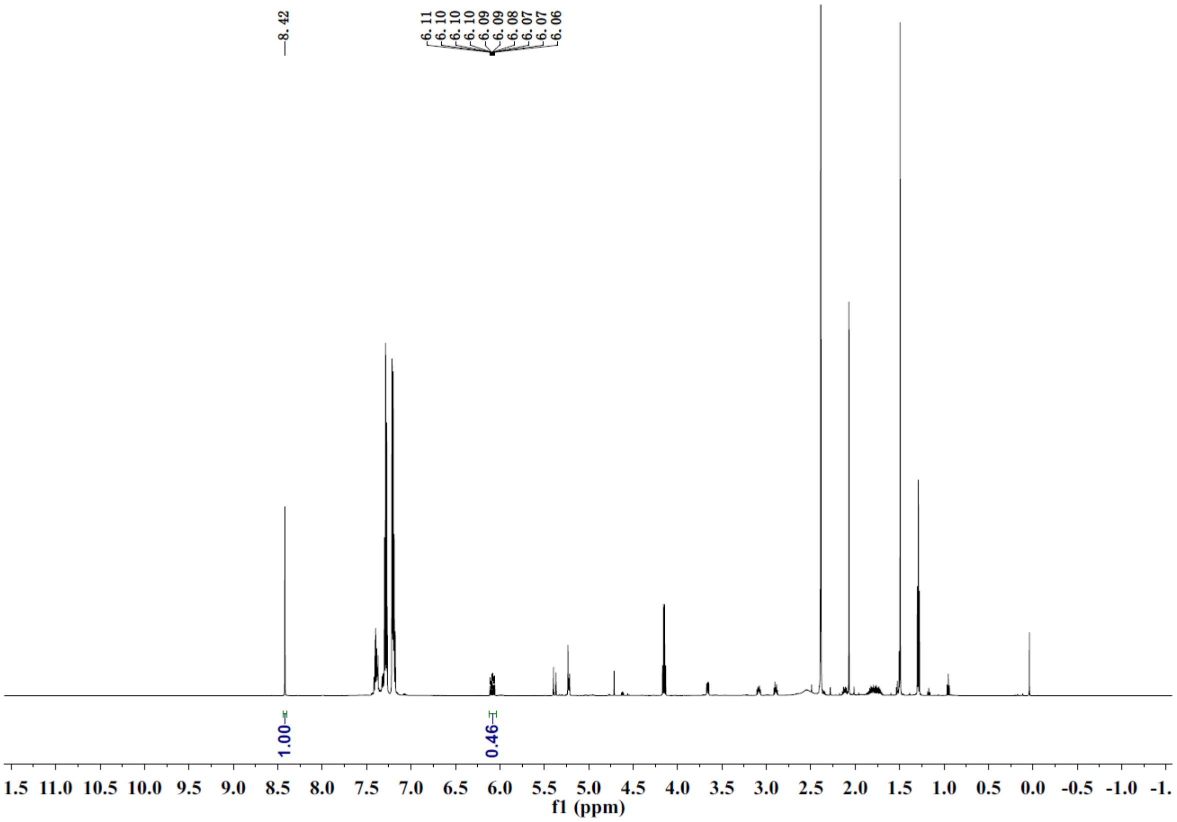

Crude ^1H NMR of entry **10**

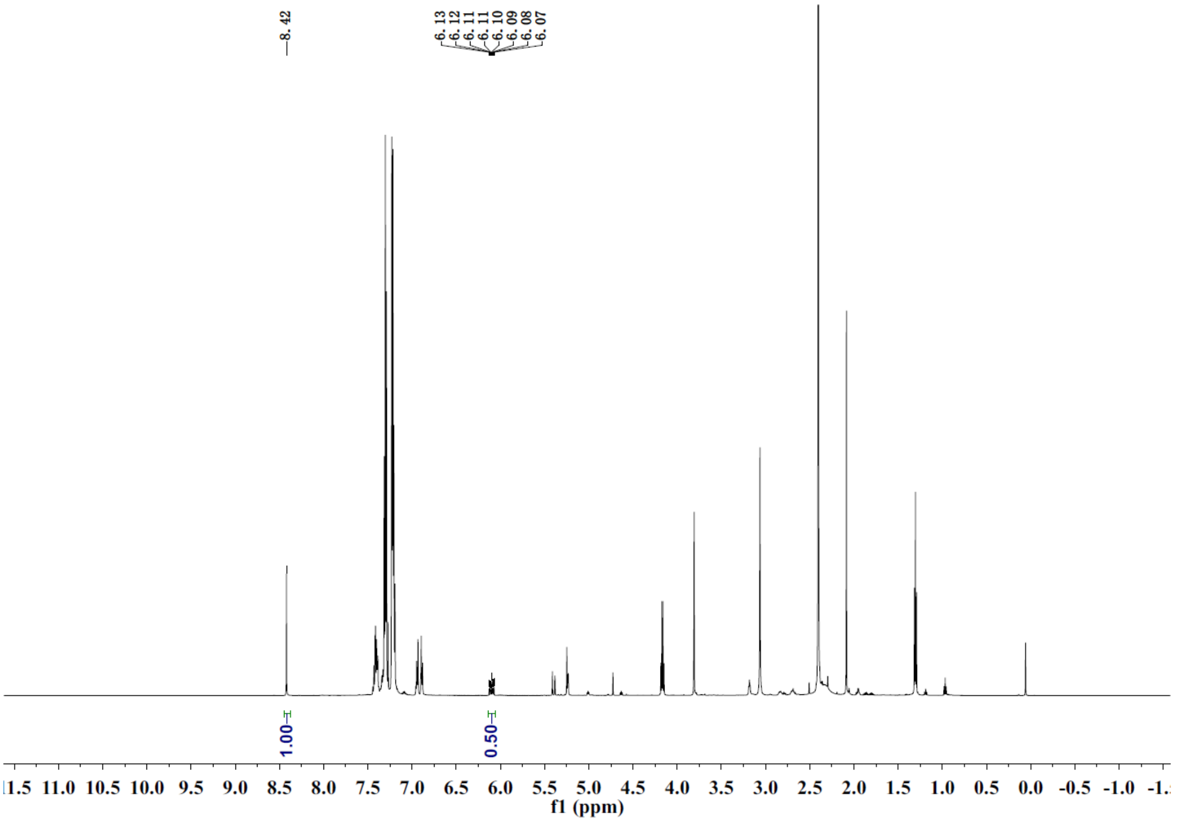

Crude ^{1}H NMR of entry **11**

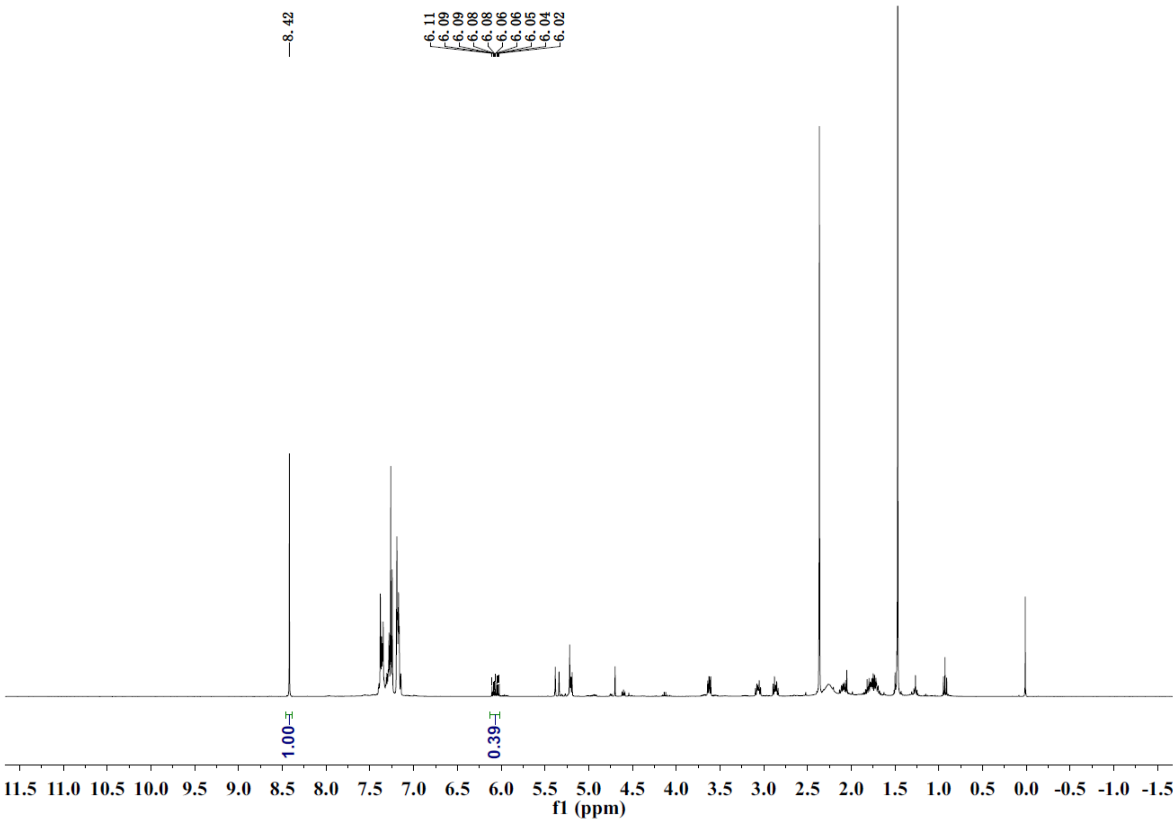

Crude ^{1}H NMR of entry **12**

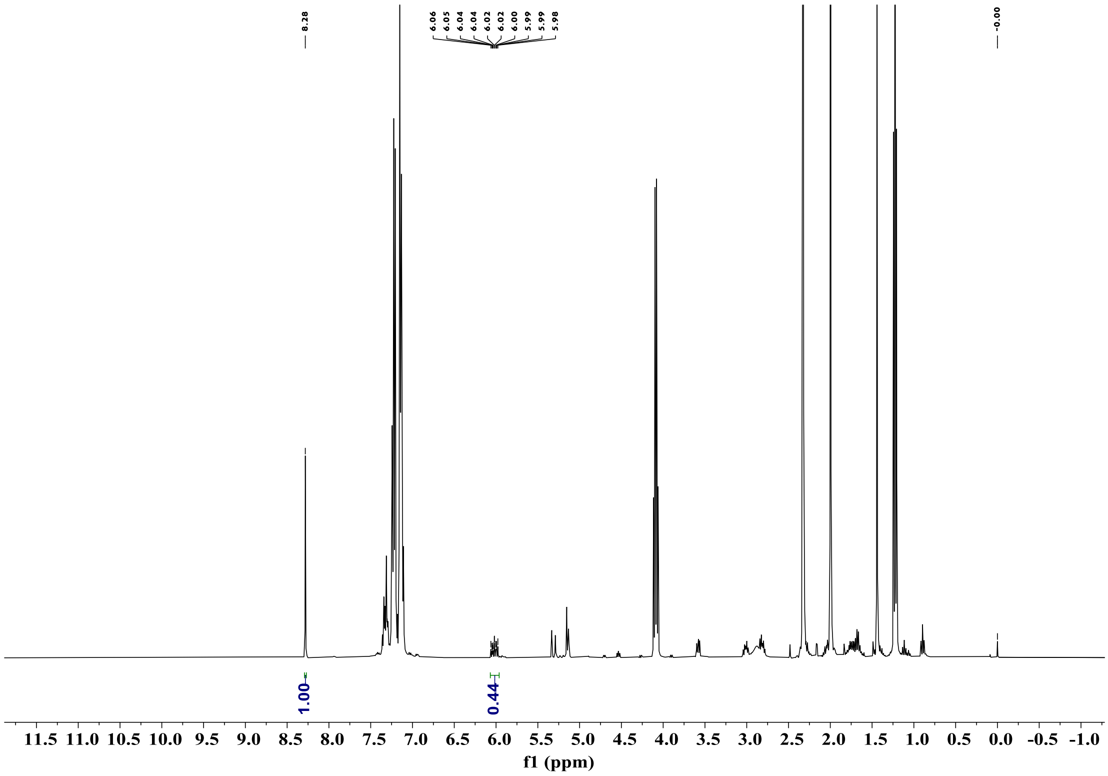

Crude ^{1}H NMR of entry **13**

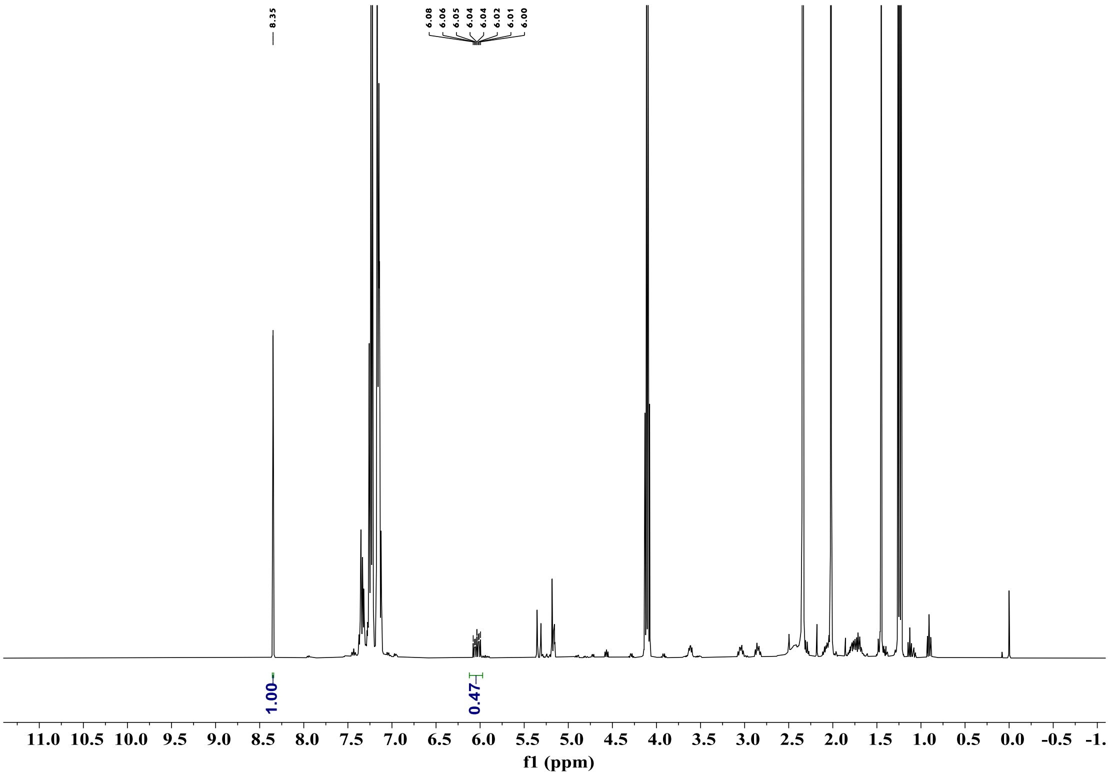

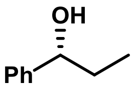

22

^{1}H NMR of 22 (600 MHz, CDCl3): δ 7.31 - 7.35 (m, 4H), 7.24 - 7.27 (m, 1H), 4.56 (t, *J* = 6.6 Hz, 1H), 1.69 - 1.84 (m, 2H), 2.05 (br, 1H), 0.90 (t, *J* = 7.4 Hz, 1H) ppm

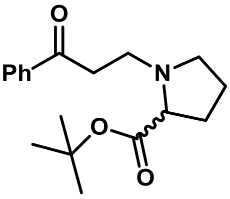

23

^{1}H NMR of 23 (600 MHz, $CDCl_3$): δ 7.93 - 7.99 (m, 2H), 7.52 - 7.58 (m, 1H), 7.42 - 7.50 (m, 2H), 3.11 - 3.31 (m, 5H), 2.85 - 2.94 (m, 1H), 2.40 - 2.49 (m, 1H), 2.07 - 2.14 (m, 1H), 1.87 - 1.96 (m, 2H), 1.79 - 1.84 (m, 1H), 1.45 (s, 9H) ppm.

^{13}C NMR of 23 (151 MHz, $CDCl_3$): δ 199.1, 173.4, 136.9, 133.1, 128.6, 128.1, 80.8, 66.5, 53.8, 49.5, 38.2, 29.3, 28.1, 23.1 ppm.

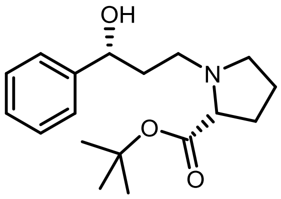

(D)-24

^{1}H NMR of (D)-24 (600 MHz, $CDCl_3$): δ 7.38 - 7.43 (m, 2H), 7.29 - 7.36 (m, 2H), 7.20 - 7.25 (m, 1H), 5.0 (dd, *J* = 3.5 Hz, 1H), 3.16 - 3.25 (m, 2H), 2.93 - 2.99 (m, 1H), 2.59 - 2.65 (m, 1H), 2.49 - 2.55 (m, 1H), 2.06 - 2.16 (m, 1H), 1.80 - 2.02 (m, 4H), 1.72 - 1.78 (m, 1H), 1.47 (s, 9H) ppm.

^{13}C NMR of (D)-24 (151 MHz, $CDCl_3$): δ 173.8, 145.1, 128.1, 126.7, 125.7, 81.2, 73.1, 66.8, 52.8, 51.5, 36.2, 29.4, 28.1, 23.3 ppm.

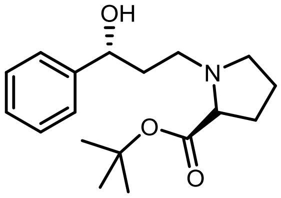

(L)-24

^{1}H NMR of (L)-24 (600 MHz, $CDCl_3$): δ 7.38 - 7.42 (m, 2H), 7.31 - 7.35 (m, 2H), 7.22 - 7.25 (m, 1H), 4.93 (dd, *J* = 12.0, 6.0 Hz, 1H), 3.42 - 3.46 (m, 1H), 3.11 - 3.17 (m, 1H), 3.06 - 3.10 (m, 1H), 2.65 - 2.69 (m, 1H), 2.24 - 2.30 (m, 1H), 2.08 - 2.14 (m, 1H), 1.80 – 1.94 (m, 4H), 1.71 - 1.76 (m, 1H), 1.50 (s, 9H) ppm.

^{13}C NMR of (L)-24 (151 MHz, $CDCl_3$): δ 172.3, 145.0, 128.2, 126.9, 125.6, 81.2, 76.1, 67.5, 54.8, 53.1, 36.6, 29.1, 28.1, 28.1, 23.1 ppm.

HRMS of the reaction mixture:

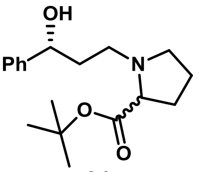

24

[M+H]⁺ 306.2064

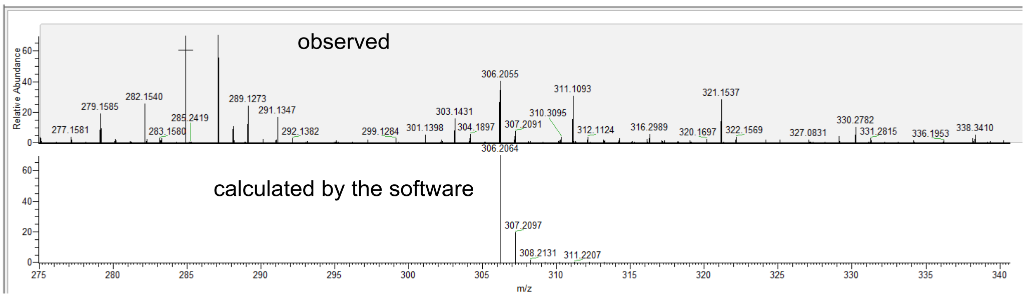

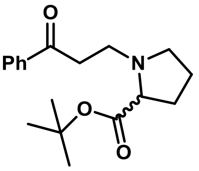

23

[M+H]⁺ 304.1907

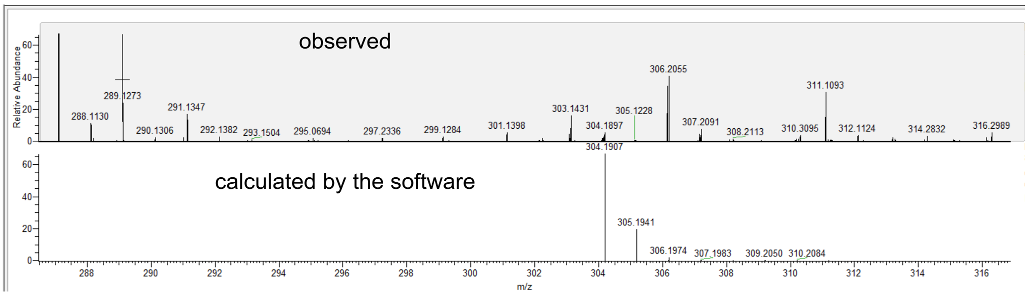

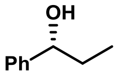

22

[M-H_2O]⁺ 119.0855

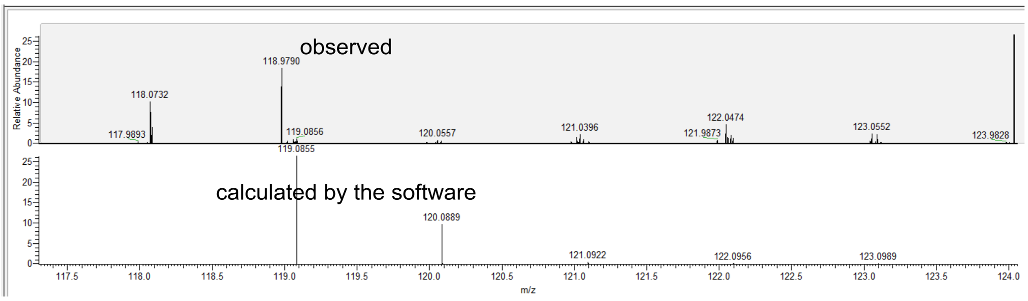

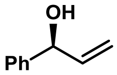

(*S*)-1

[M+H]⁺ 135.0804

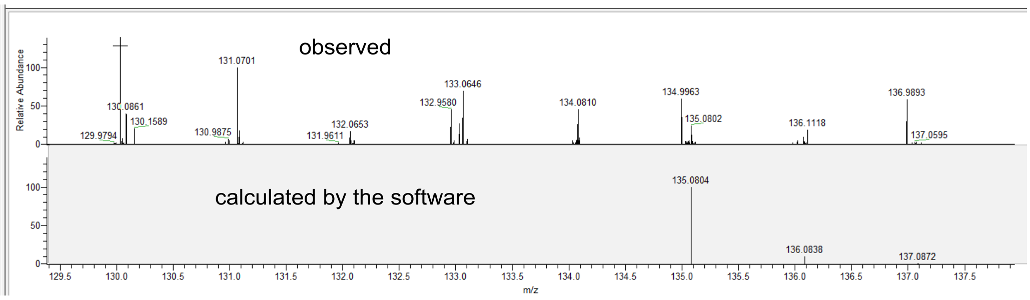

HPLC of 1 (OJ-H, 0.46*25 cm, 5μm, hexane/isopropanol = 90/10, flow 1.0 mL/min, detection at 210 nm) retention time = 9.029 min and 10.639 min.

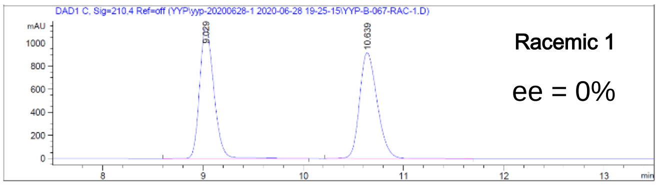

Signal 2: DAD1 C, Sig=210,4 Ref=off

Peak #	RetTime [min]	Type	Width [min]	Area [mAU*s]	Height [mAU]	Area %
1	9.029	BB	0.1537	1.12644e4	1135.40259	50.1072
2	10.639	BB	0.1883	1.12162e4	919.25592	49.8928

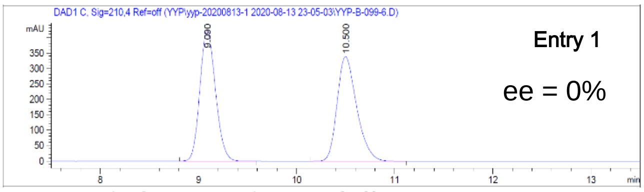

Signal 2: DAD1 C, Sig=210,4 Ref=off

Peak #	RetTime [min]	Type	Width [min]	Area [mAU*s]	Height [mAU]	Area %
1	9.090	BB	0.1704	4680.00293	425.10657	49.9350
2	10.500	BB	0.2094	4692.19287	339.20691	50.0650

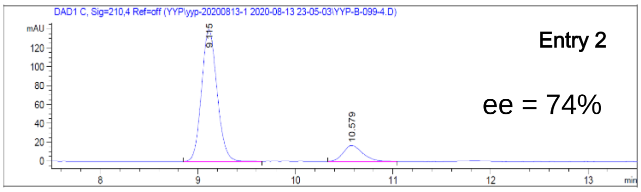

Signal 2: DAD1 C, Sig=210,4 Ref=off

Peak #	RetTime [min]	Type	Width [min]	Area [mAU*s]	Height [mAU]	Area %
1	9.115	BB	0.1704	1535.54883	139.54103	87.0205
2	10.579	BB	0.2030	229.03424	16.80051	12.9795

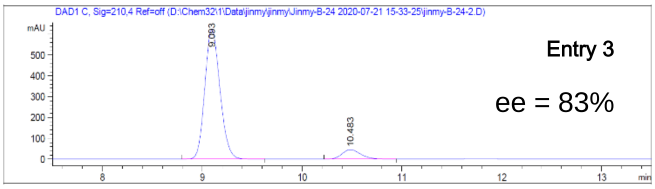

Signal 2: DAD1 C, Sig=210,4 Ref=off

Peak #	RetTime [min]	Type	Width [min]	Area [mAU*s]	Height [mAU]	Area %
1	9.093	BB	0.1625	6545.37939	623.19421	91.6773
2	10.483	BB	0.1996	594.20544	45.74141	8.3227

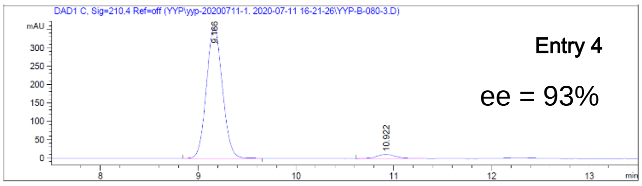

Signal 2: DAD1 C, Sig=210,4 Ref=off

Peak #	RetTime [min]	Type	Width [min]	Area [mAU*s]	Height [mAU]	Area %
1	9.166	BB	0.1763	4036.34082	355.87466	96.4984
2	10.922	BB	0.2013	146.46463	11.29414	3.5016

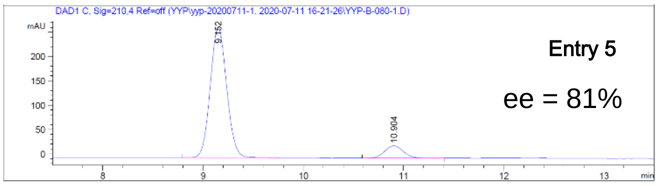

Signal 2: DAD1 C, Sig=210,4 Ref=off

Peak #	RetTime [min]	Type	Width [min]	Area [mAU*s]	Height [mAU]	Area %
1	9.152	BB	0.1766	3033.52124	266.89523	90.2658
2	10.904	BB	0.2007	327.13202	25.31721	9.7342

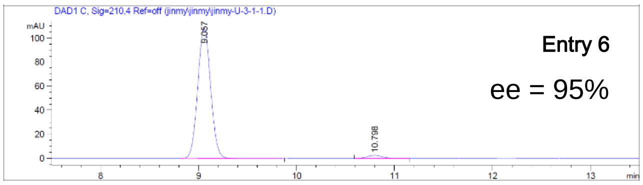

Signal 2: DAD1 C, Sig=210,4 Ref=off

Peak #	RetTime [min]	Type	Width [min]	Area [mAU*s]	Height [mAU]	Area %
1	9.057	BB	0.1426	994.07098	108.81385	97.2985
2	10.798	BB	0.1709	27.60073	2.53704	2.7015

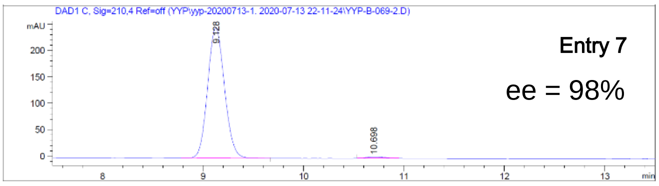

Signal 2: DAD1 C, Sig=210,4 Ref=off

Peak #	RetTime [min]	Type	Width [min]	Area [mAU*s]	Height [mAU]	Area %
1	9.128	BB	0.1817	2873.27954	247.10349	98.8227
2	10.698	MM R	0.2182	34.23132	2.61509	1.1773

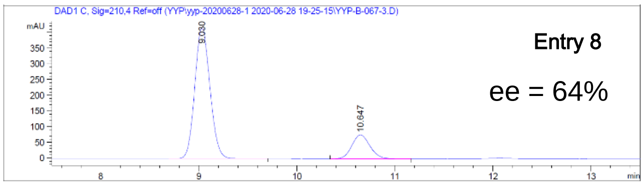

Signal 2: DAD1 C, Sig=210,4 Ref=off

Peak #	RetTime [min]	Type	Width [min]	Area [mAU*s]	Height [mAU]	Area %
1	9.030	BB	0.1672	4470.95557	416.74289	82.2282
2	10.647	BB	0.1982	966.30017	76.06502	17.7718

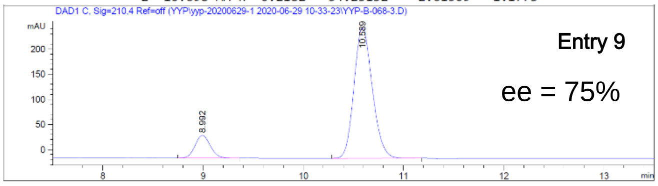

Signal 2: DAD1 C, Sig=210,4 Ref=off

Peak #	RetTime [min]	Type	Width [min]	Area [mAU*s]	Height [mAU]	Area %
1	8.992	BB	0.1649	474.95654	45.08772	12.5730
2	10.589	BB	0.1962	3302.64746	259.97778	87.4270

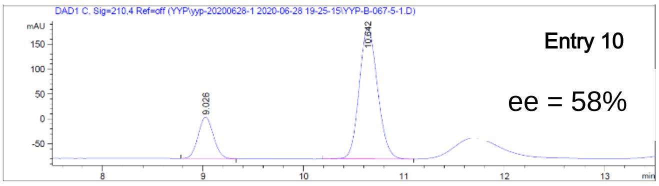

Signal 2: DAD1 C, Sig=210,4 Ref=off

Peak #	RetTime [min]	Type	Width [min]	Area [mAU*s]	Height [mAU]	Area %
1	9.026	BB	0.1656	879.96185	83.09081	20.8450
2	10.642	BB	0.1980	3341.48633	263.32559	79.1550

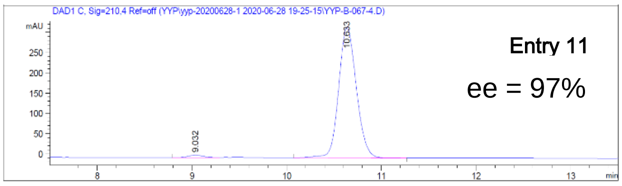

Signal 2: DAD1 C, Sig=210,4 Ref=off

Peak #	RetTime [min]	Type	Width [min]	Area [mAU*s]	Height [mAU]	Area %
1	9.032	BB	0.1649	62.74325	6.05340	1.4804
2	10.633	BB	0.1996	4175.50244	321.43549	98.5196

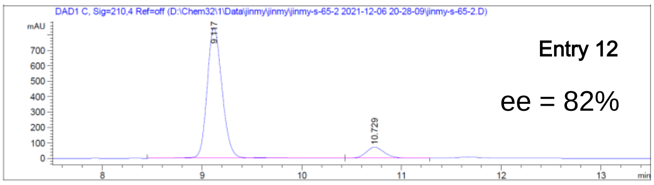

Signal 2: DAD1 C, Sig=210,4 Ref=off

Peak #	RetTime [min]	Type	Width [min]	Area [mAU*s]	Height [mAU]	Area %
1	9.117	BV R	0.1588	8878.36230	845.96808	90.7265
2	10.729	VB	0.1975	907.49030	70.80131	9.2735

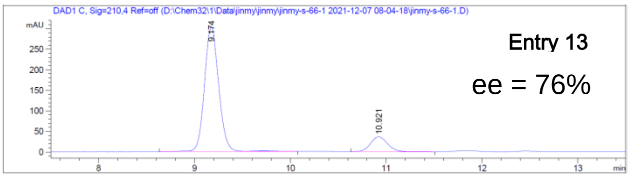

Signal 2: DAD1 C, Sig=210,4 Ref=off

Peak #	RetTime [min]	Type	Width [min]	Area [mAU*s]	Height [mAU]	Area %
1	9.174	BV R	0.1569	3136.33569	304.16455	87.7913
2	10.921	BB	0.1877	436.15665	35.90301	12.2087

4. Kinetic resolution of allylic alcohols via borrowing hydrogen cascade

General procedure II for kinetic resolution of allylic alcohols.

In an argon filled glovebox, an oven-dried resealable tube equipped with a magnetic stir bar was charged with the Ru-catalyst (0.1-0.25 mol%), racemic allylic alcohol (0.2 mmol), an amine nucleophile (0.12 mmol) or without an external nucleophile, toluene (2.0 mL) and dichloromethane (20 μL). ^{t}BuOK (30 μL, 1.0 M in *t*-BuOH) was added into the above reaction mixture. After reaching a 45% to 65% conversion, the reaction was quenched by H_2O and extracted with ethyl acetate (3×5 mL). The combined organic layers were dried over Na_2SO_4 and concentrated under reduced pressure. 1,4-dinitrobenzene (8.4 mg, 0.05 mmol) was added into the crude residue as the internal standard to determine the crude ^{1}H NMR yield of the recovered allylic alcohol. The reaction conversion (c) was calculated by the following formula: c = 1- (^{1}H NMR yield$_{\text{(recovered alcohols)}}$)%. The enantiomeric excess (ee) of the recovered allylic alcohol was determined by HPLC analysis. The selectivity factor (*s*) of the kinetic resolution was thus calculated by the formula: *s* = In[(1-c)(1-ee)]/In[(1-c)(1+ee)].

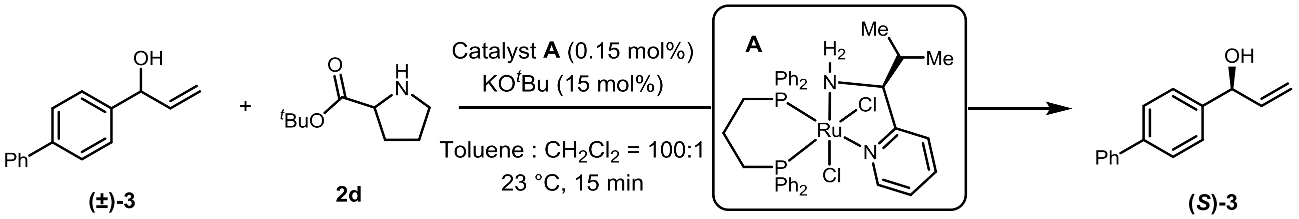

The general procedure **II** was followed. The conversion of **(±)-3** was determined by crude ^{1}H NMR (52% conversion, 48% yield, 88% ee, *s* = 28).

Crude **^{1}H NMR**

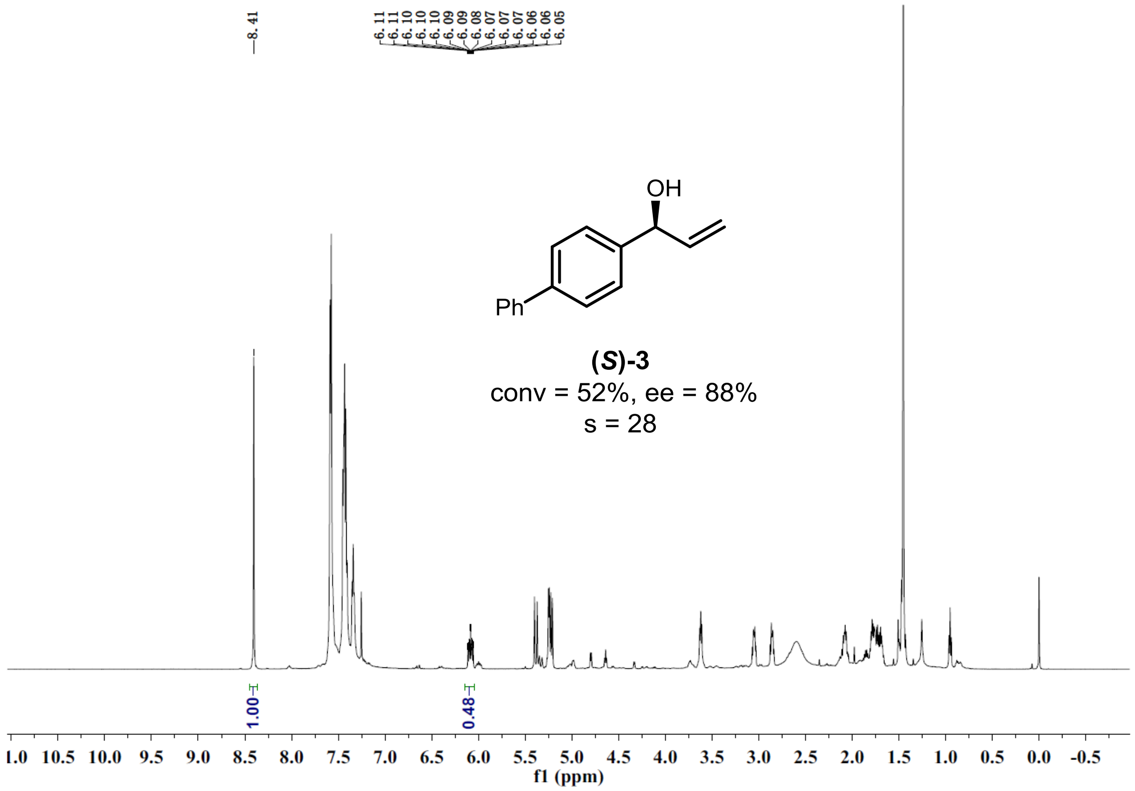

HPLC (AD-H, 0.46*25 cm, 5μm, hexane/isopropanol = 95/5, flow 1 mL/min, detection at 210 nm) retention time = 12.864 min (major) and 14.260 min (minor).

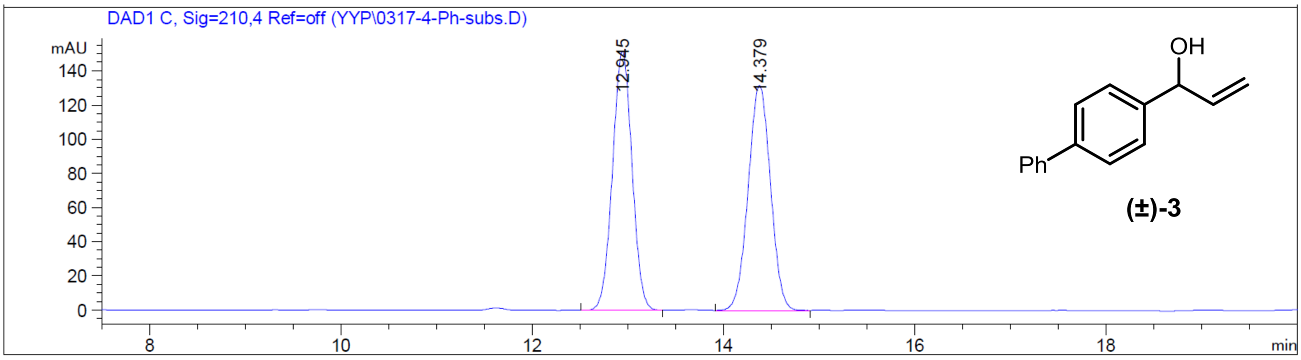

Signal 2: DAD1 C, Sig=210,4 Ref=off

Peak #	RetTime [min]	Type	Width [min]	Area [mAU*s]	Height [mAU]	Area %
1	12.945	BB	0.2219	2156.09106	151.63548	50.1881
2	14.379	BB	0.2533	2139.92651	131.87219	49.8119

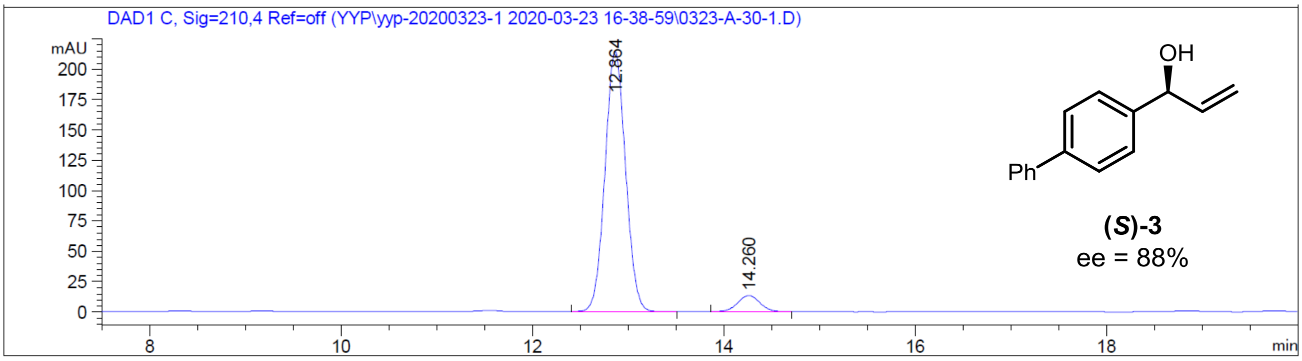

Signal 2: DAD1 C, Sig=210,4 Ref=off

Peak #	RetTime [min]	Type	Width [min]	Area [mAU*s]	Height [mAU]	Area %
1	12.864	BB	0.2324	3203.68872	214.27786	93.7995
2	14.260	BB	0.2482	211.77692	13.26795	6.2005

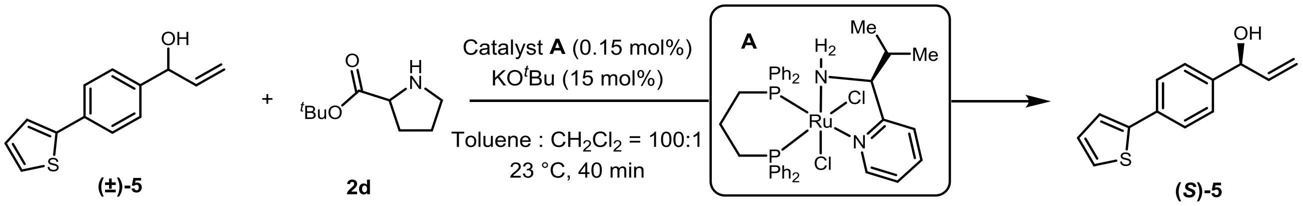

The general procedure **II** was followed. The conversion of **(±)-5** was determined by crude ^{1}H NMR (64% conversion, 36% yield, 97% ee, *s* = 13).

Crude **^{1}H NMR**

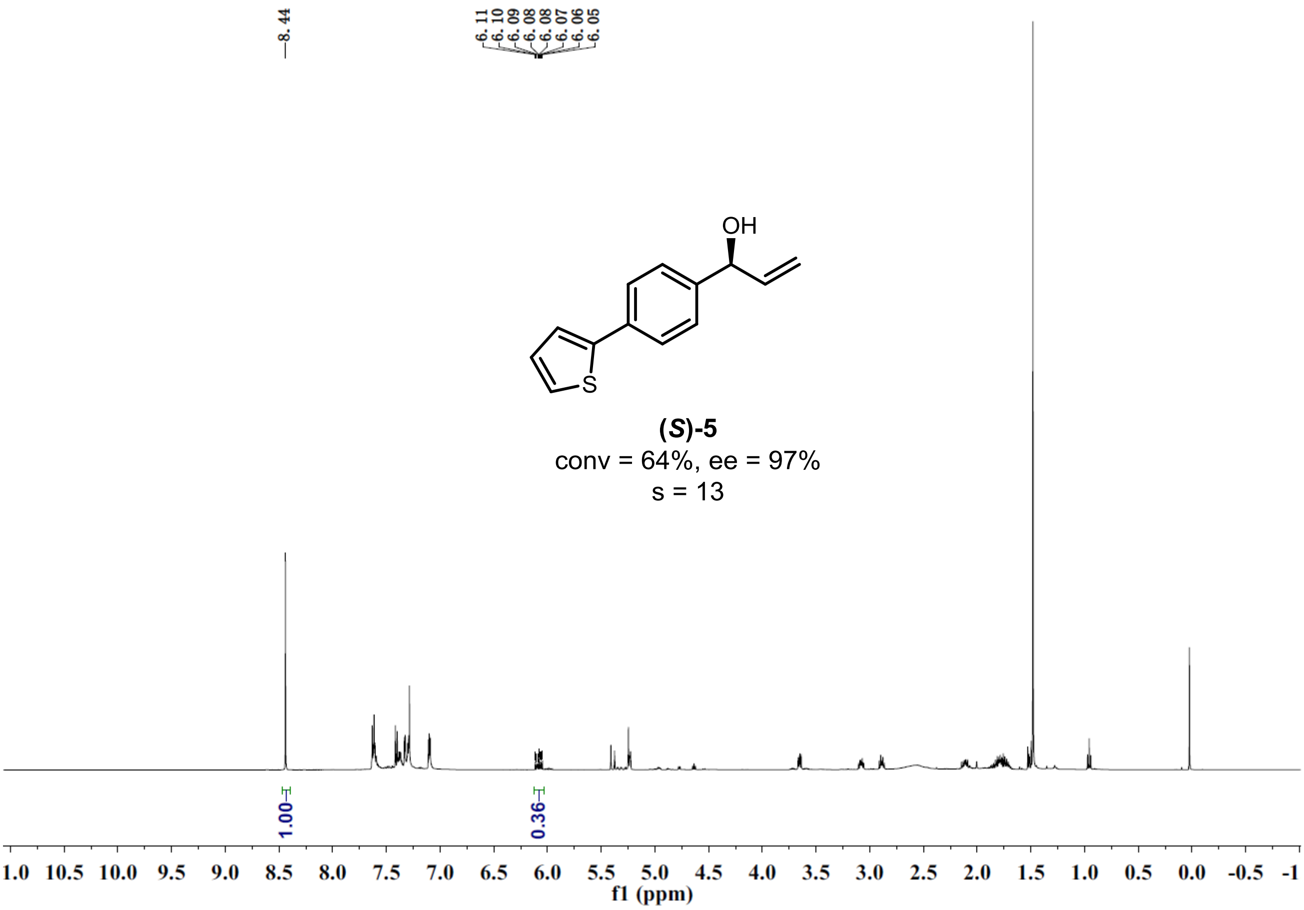

HPLC (AD-H, 0.46*25 cm, 5μm, hexane/isopropanol = 90/10, flow 1 mL/min, detection at 210 nm) retention time = 9.737 min (major) and 10.656 min (minor).

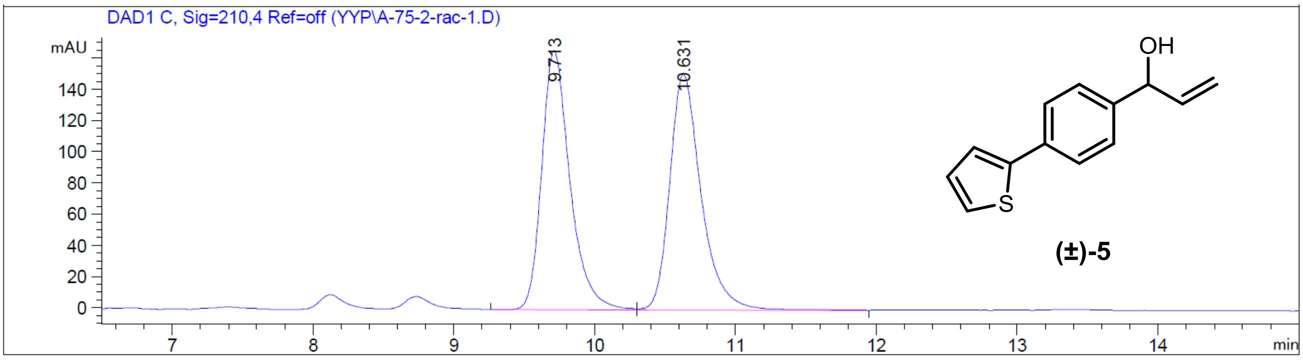

Signal 2: DAD1 C, Sig=210,4 Ref=off

Peak #	RetTime [min]	Type	Width [min]	Area [mAU*s]	Height [mAU]	Area %
1	9.713	BV	0.2149	2316.33691	165.93053	49.6755
2	10.631	VB	0.2333	2346.60278	150.97260	50.3245

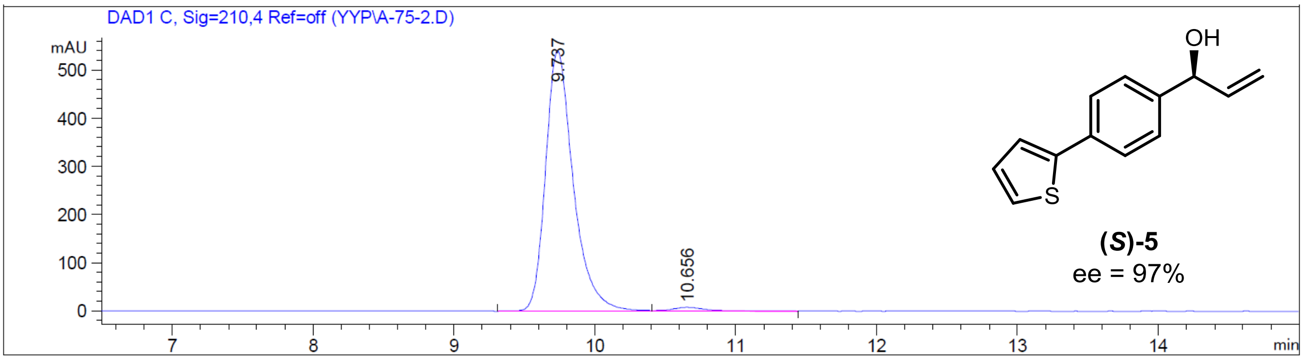

Signal 2: DAD1 C, Sig=210,4 Ref=off

Peak #	RetTime [min]	Type	Width [min]	Area [mAU*s]	Height [mAU]	Area %
1	9.737	BV R	0.2081	7422.06055	540.82062	98.3848
2	10.656	VB E	0.2331	121.84641	7.84751	1.6152

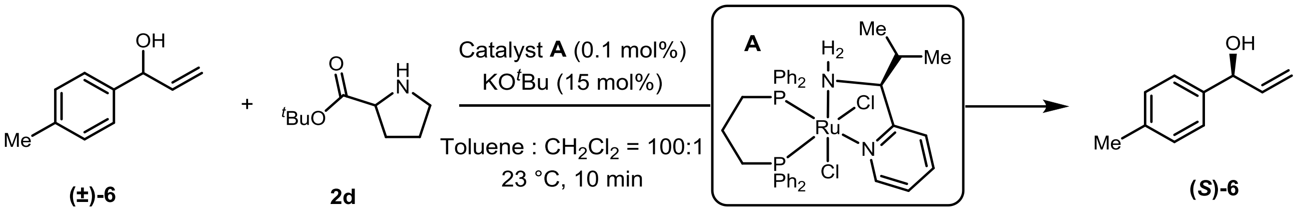

The general procedure **II** was followed. The conversion of **(±)-6** was determined by crude ^{1}H NMR (56% conversion, 44% yield, 91% ee, *s* = 19).

Crude **^{1}H NMR**

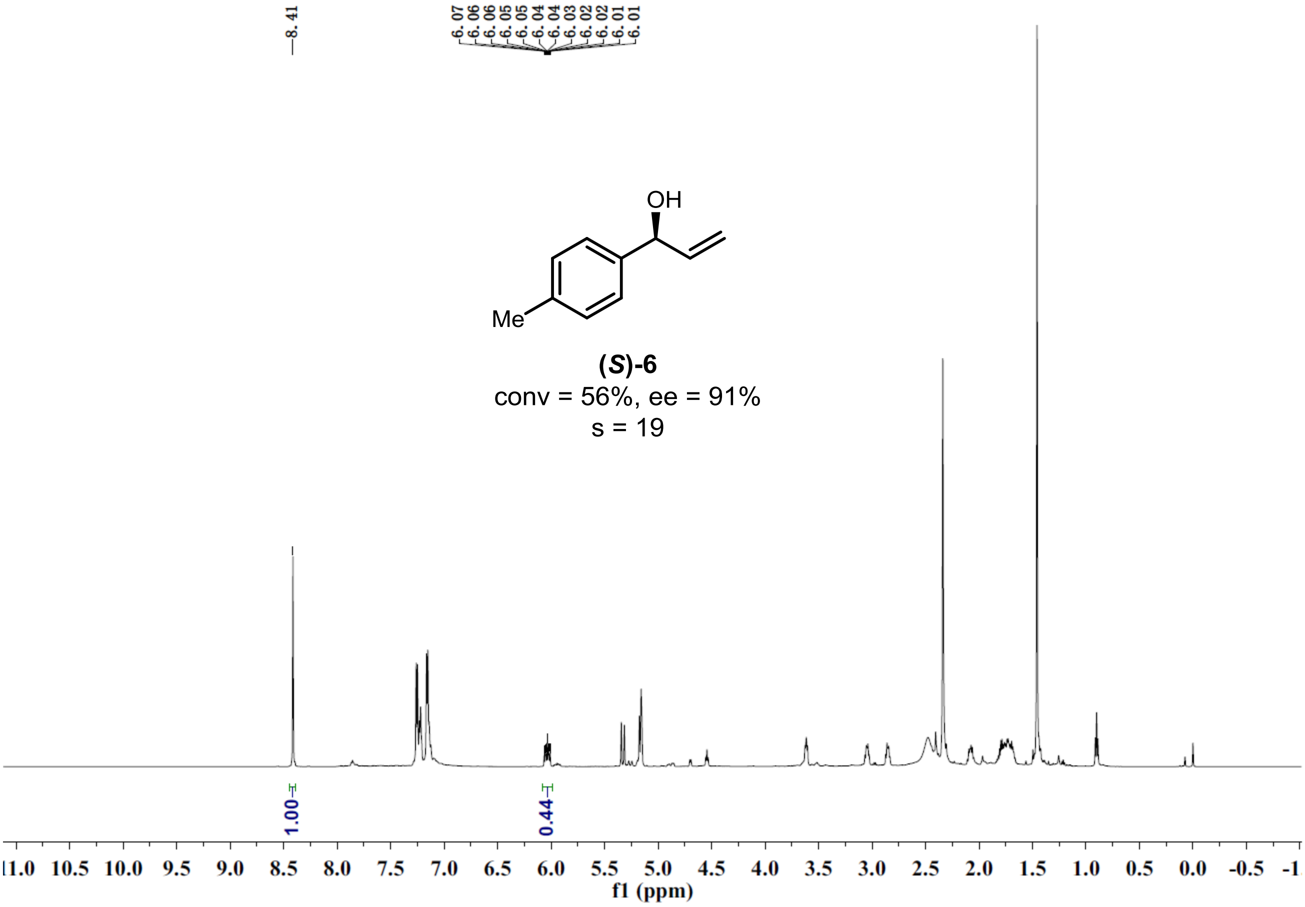

HPLC (OJ-H, 0.46*25 cm, 5μm, hexane/isopropanol = 95/5, flow 1 mL/min, detection at 210 nm) retention time = 13.874 min (major) and 16.942 min (minor).

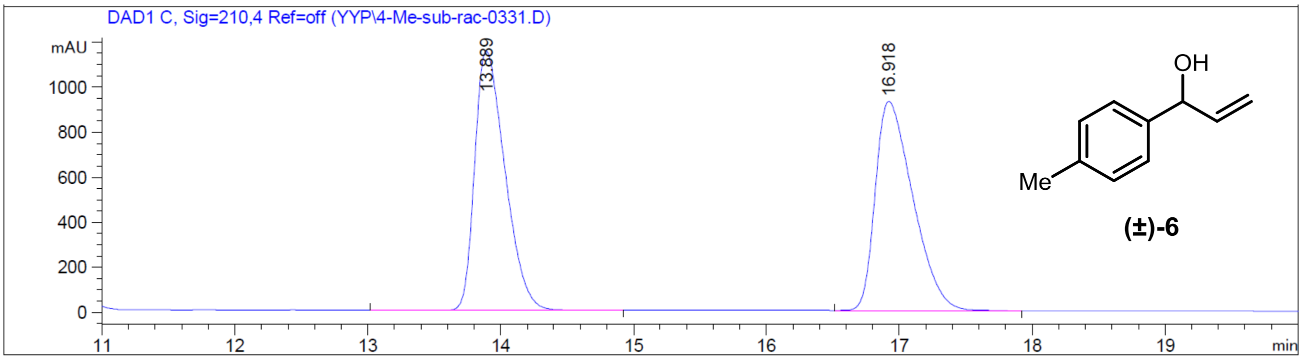

Signal 2: DAD1 C, Sig=210,4 Ref=off

Peak #	RetTime [min]	Type	Width [min]	Area [mAU*s]	Height [mAU]	Area %
1	13.889	VB R	0.2445	1.82340e4	1153.16736	49.9254
2	16.918	BB	0.3036	1.82885e4	929.30920	50.0746

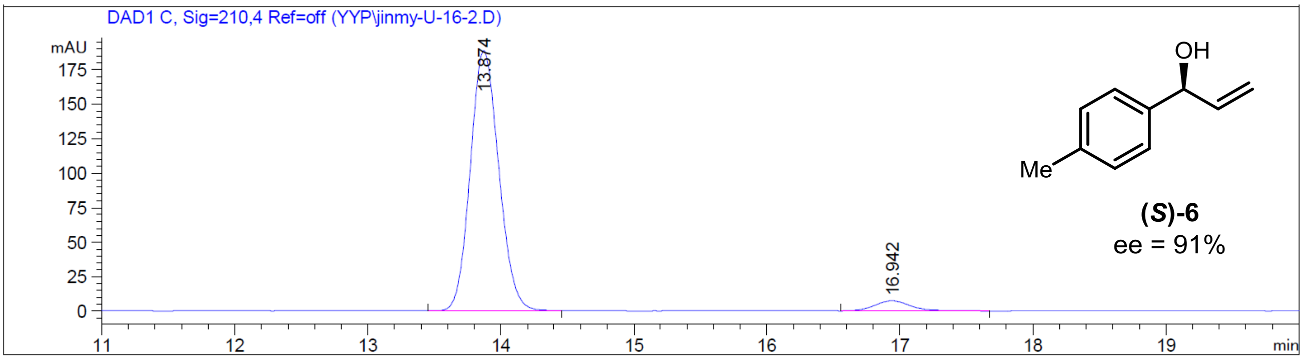

Signal 2: DAD1 C, Sig=210,4 Ref=off

Peak #	RetTime [min]	Type	Width [min]	Area [mAU*s]	Height [mAU]	Area %
1	13.874	BB	0.2318	2807.42163	188.44055	95.3187
2	16.942	BB	0.2841	137.87889	7.30781	4.6813

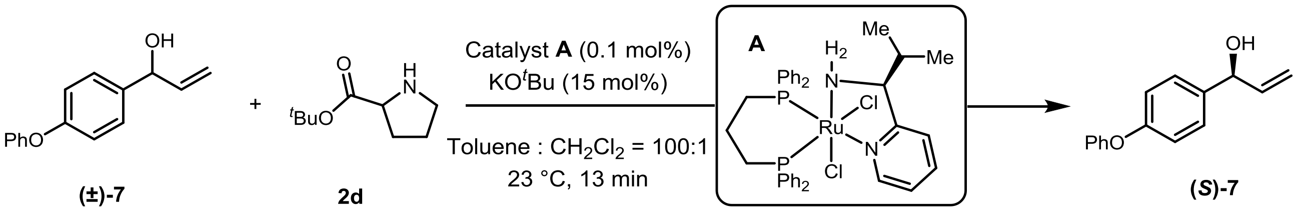

The general procedure **II** was followed. The conversion of **(±)-7** was determined by crude ^{1}H NMR (57% conversion, 43% yield, 95% ee, *s* = 22).

Crude **^{1}H NMR**

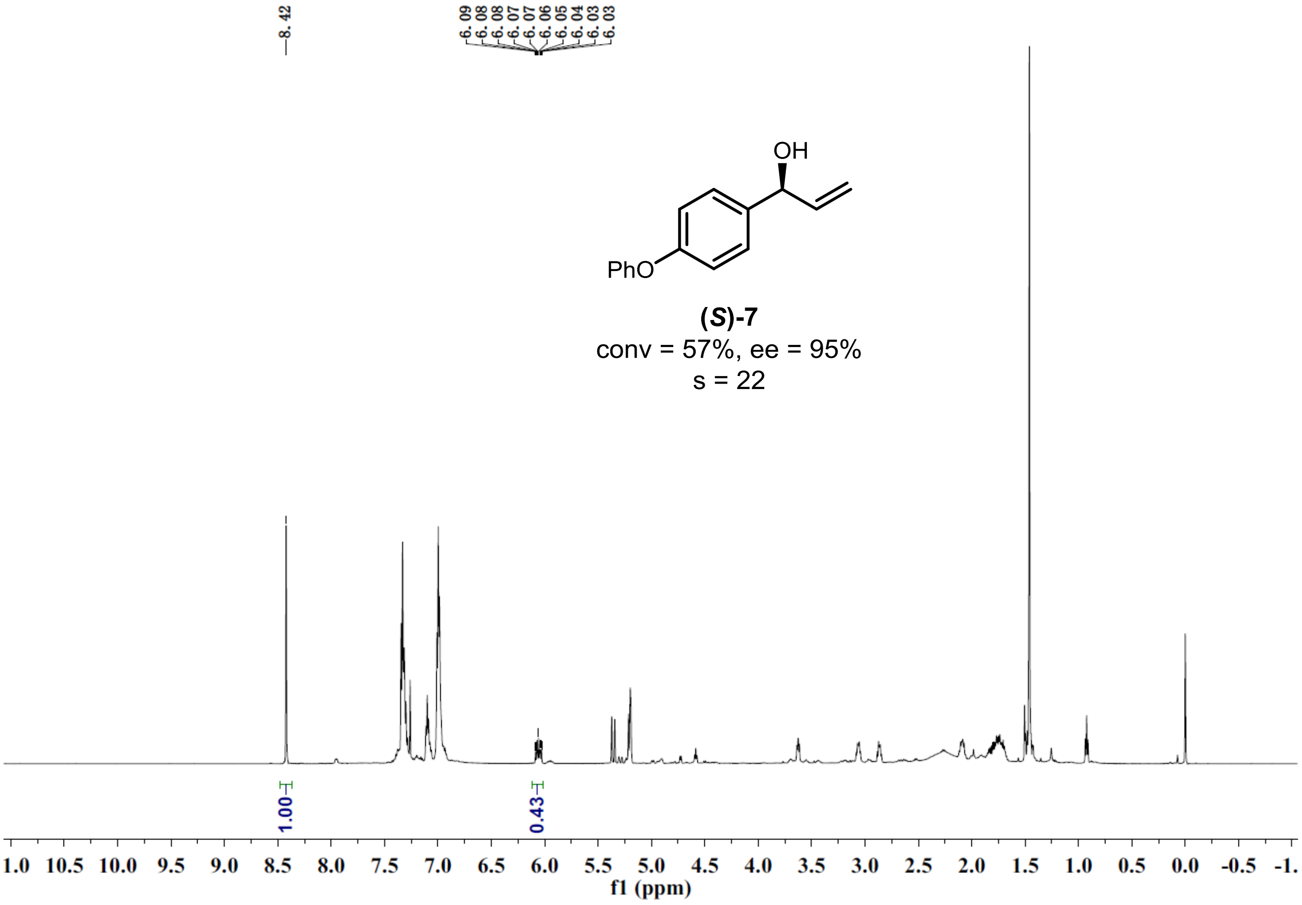

HPLC (OJ-H, 0.46*25 cm, 5µm, hexane/isopropanol = 90/10, flow 1 mL/min, detection at 210 nm) retention time = 15.015 min (minor) and 18.004 min (major).

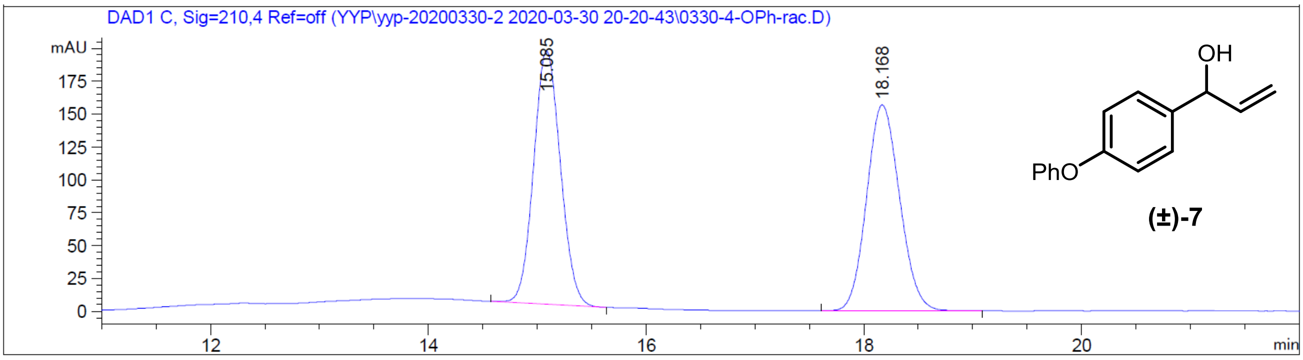

Signal 2: DAD1 C, Sig=210,4 Ref=off

Peak #	RetTime [min]	Type	Width [min]	Area [mAU*s]	Height [mAU]	Area %
1	15.085	BB	0.2648	3286.50488	192.87431	49.9061
2	18.168	BB	0.3278	3298.87231	156.60962	50.0939

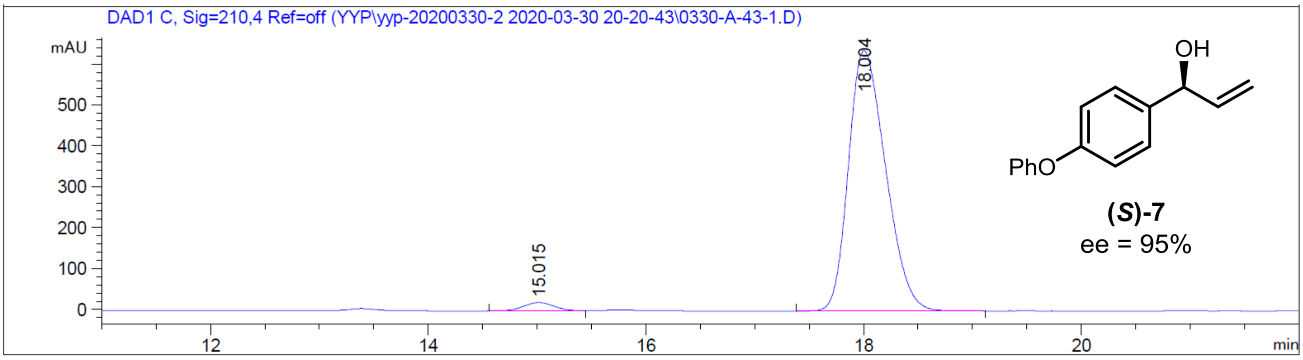

Signal 2: DAD1 C, Sig=210,4 Ref=off

Peak #	RetTime [min]	Type	Width [min]	Area [mAU*s]	Height [mAU]	Area %
1	15.015	BB	0.2962	386.17239	20.09930	2.5028
2	18.004	BB	0.3698	1.50437e4	636.39227	97.4972

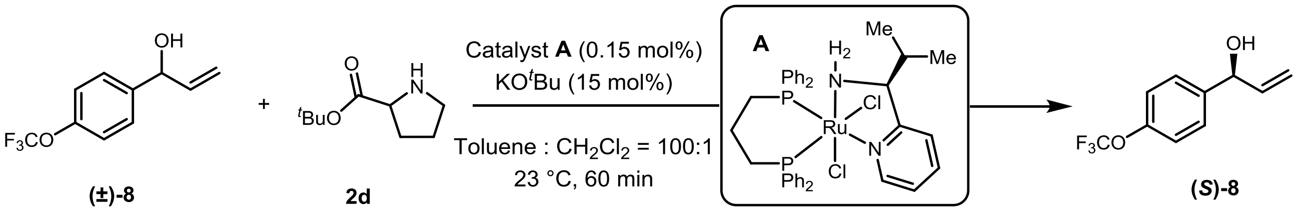

The general procedure **II** was followed. The conversion of **(±)-8** was determined by crude ^{1}H NMR (57% conversion, 43% yield, 91% ee, *s* = 17).

Crude **^{1}H NMR**

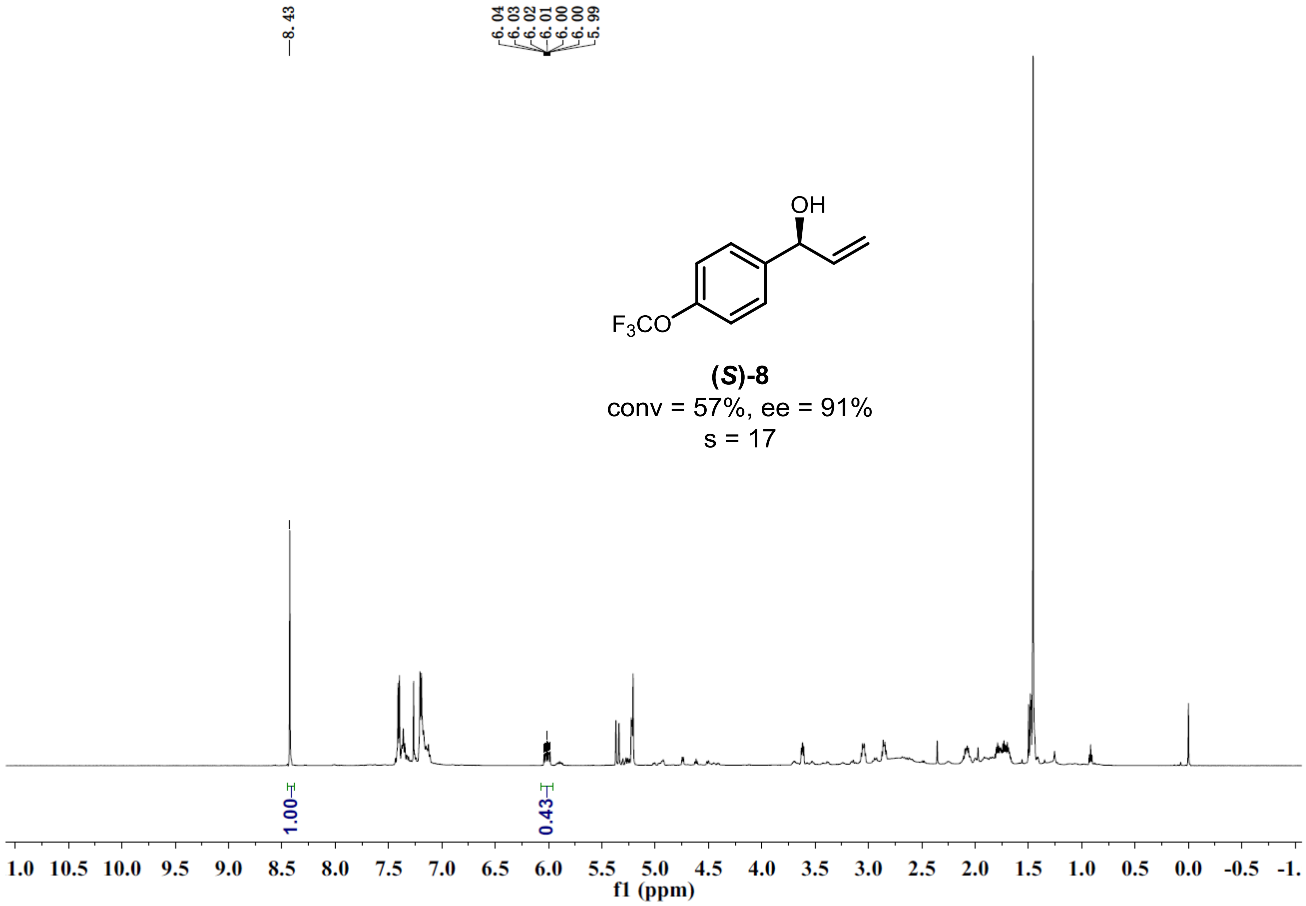

HPLC (OJ-H, 0.46*25 cm, 5μm, hexane/isopropanol = 98/2, flow 1 mL/min, detection at 210 nm) retention time = 11.475 min (major) and 12.308 min (minor).

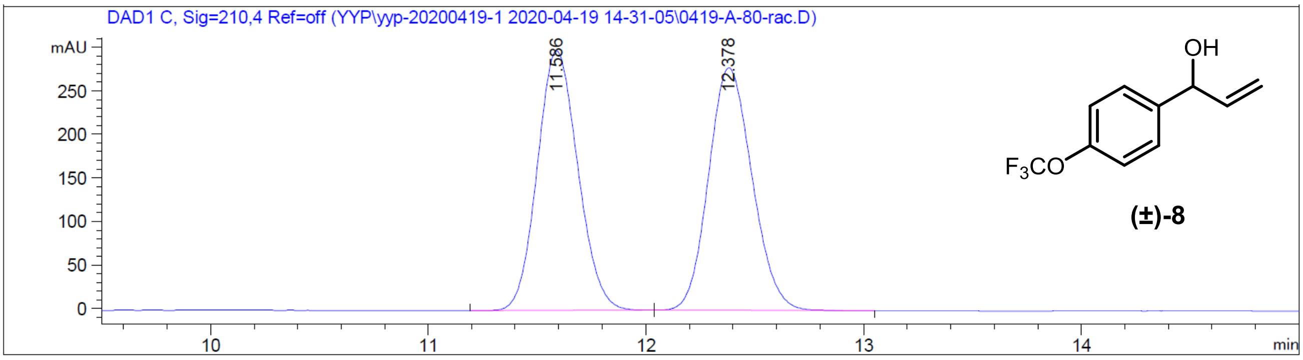

Signal 2: DAD1 C, Sig=210,4 Ref=off

Peak #	RetTime [min]	Type	Width [min]	Area [mAU*s]	Height [mAU]	Area %
1	11.586	BB	0.2035	3871.42554	298.17807	50.0411
2	12.378	BB	0.2160	3865.06494	278.29514	49.9589

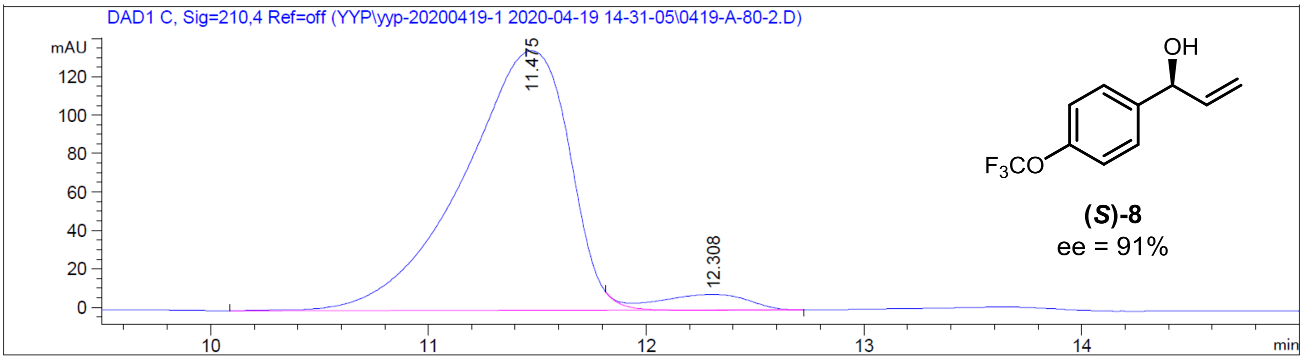

Signal 2: DAD1 C, Sig=210,4 Ref=off

Peak #	RetTime [min]	Type	Width [min]	Area [mAU*s]	Height [mAU]	Area %
1	11.475	BV R	0.5069	4594.96484	135.08310	95.2661
2	12.308	VB E	0.3859	228.33096	8.12499	4.7339

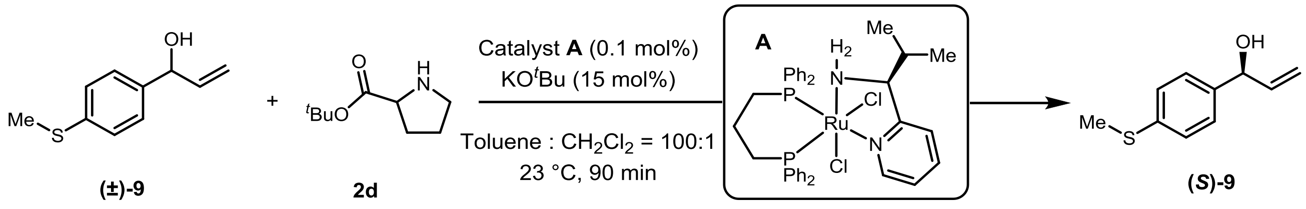

The general procedure **II** was followed. The conversion of **(±)-9** was determined by crude ^{1}H NMR (58% conversion, 42% yield, 93% ee, *s* = 17).

Crude **^{1}H NMR**

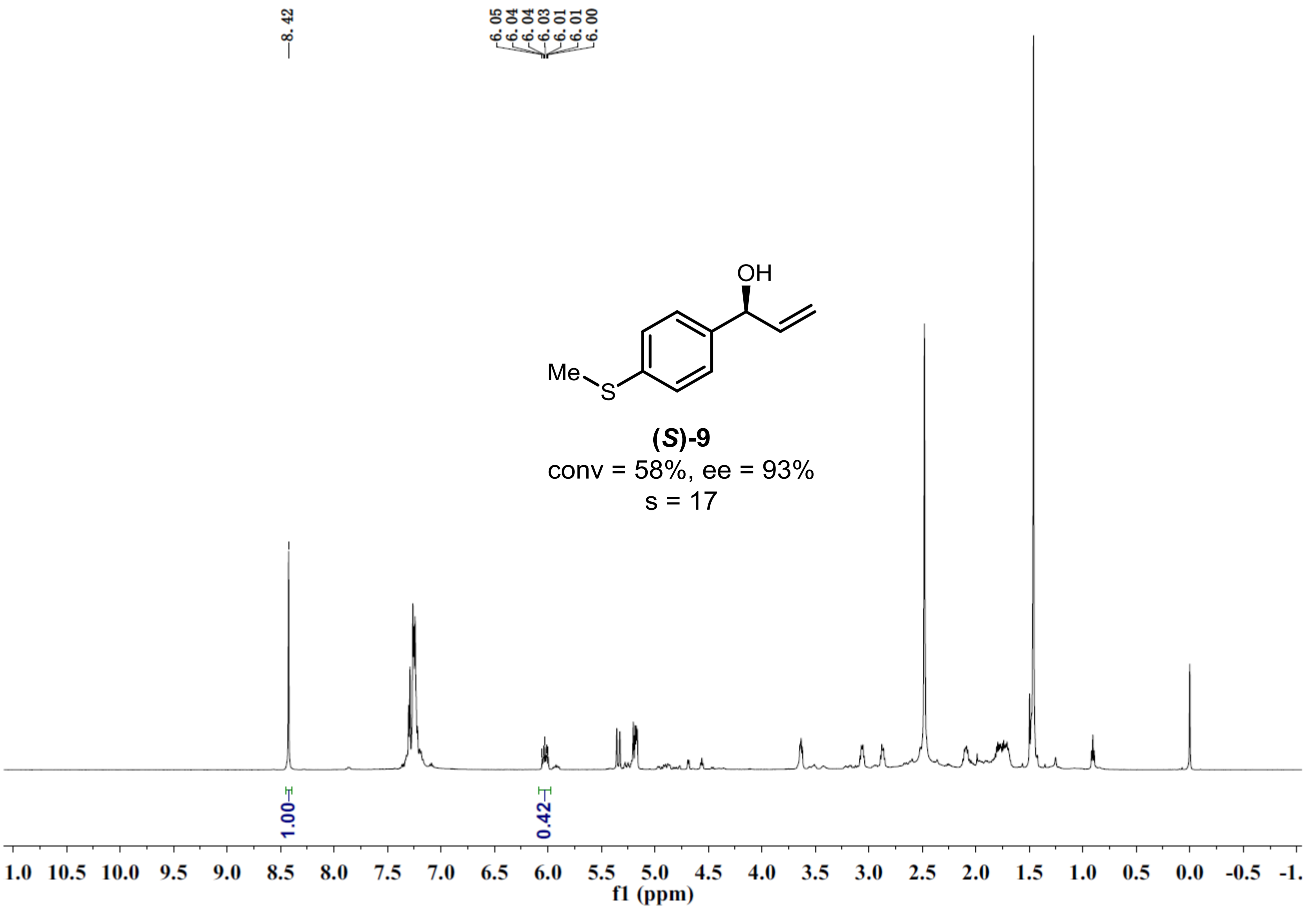

HPLC (AD-H, 0.46*25 cm, 5μm, hexane/isopropanol = 95/5, flow 1 mL/min, detection at 210 nm) retention time = 14.107 min (minor) and 15.048 min (major).

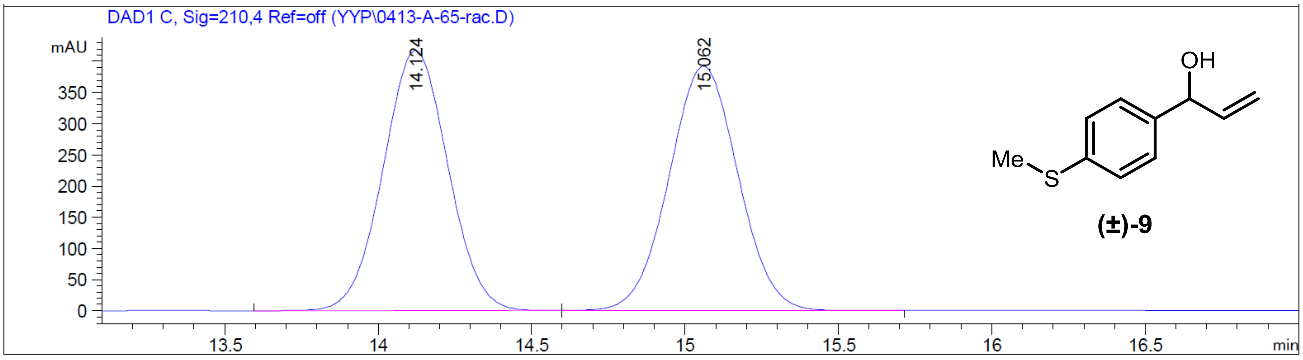

Signal 2: DAD1 C, Sig=210,4 Ref=off

Peak #	RetTime [min]	Type	Width [min]	Area [mAU*s]	Height [mAU]	Area %
1	14.124	BB	0.2293	6130.90918	417.43484	49.9758
2	15.062	BB	0.2446	6136.85645	392.15427	50.0242

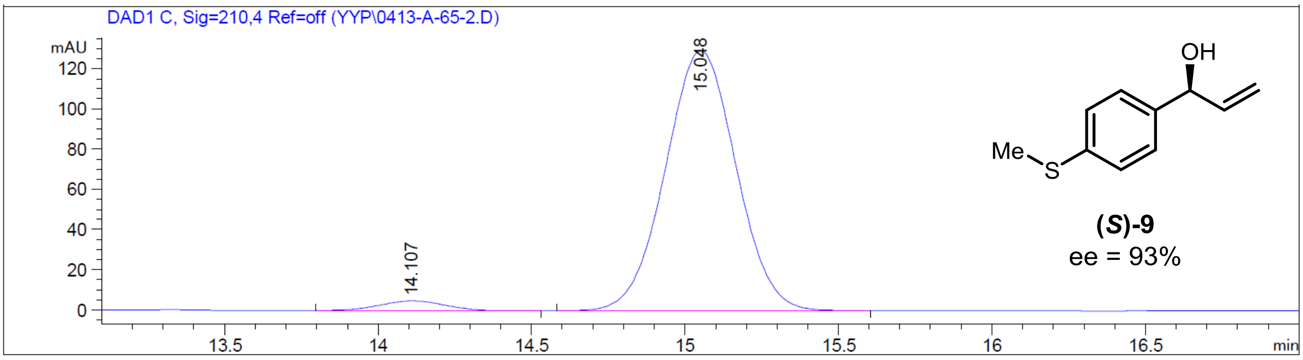

Signal 2: DAD1 C, Sig=210,4 Ref=off

Peak #	RetTime [min]	Type	Width [min]	Area [mAU*s]	Height [mAU]	Area %
1	14.107	BB	0.2318	72.35355	4.85786	3.3736
2	15.048	BB	0.2488	2072.32788	129.44160	96.6264

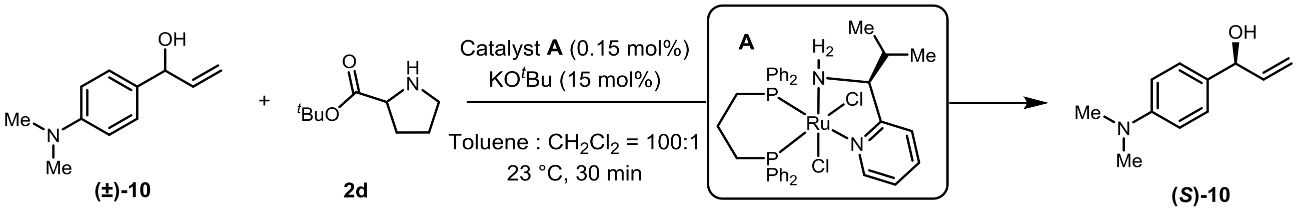

The general procedure **II** was followed. The conversion of **(±)-10** was determined by crude ^{1}H NMR (58% conversion, 42% yield, 99.9% ee, *s* = 44).

Crude **^{1}H NMR**

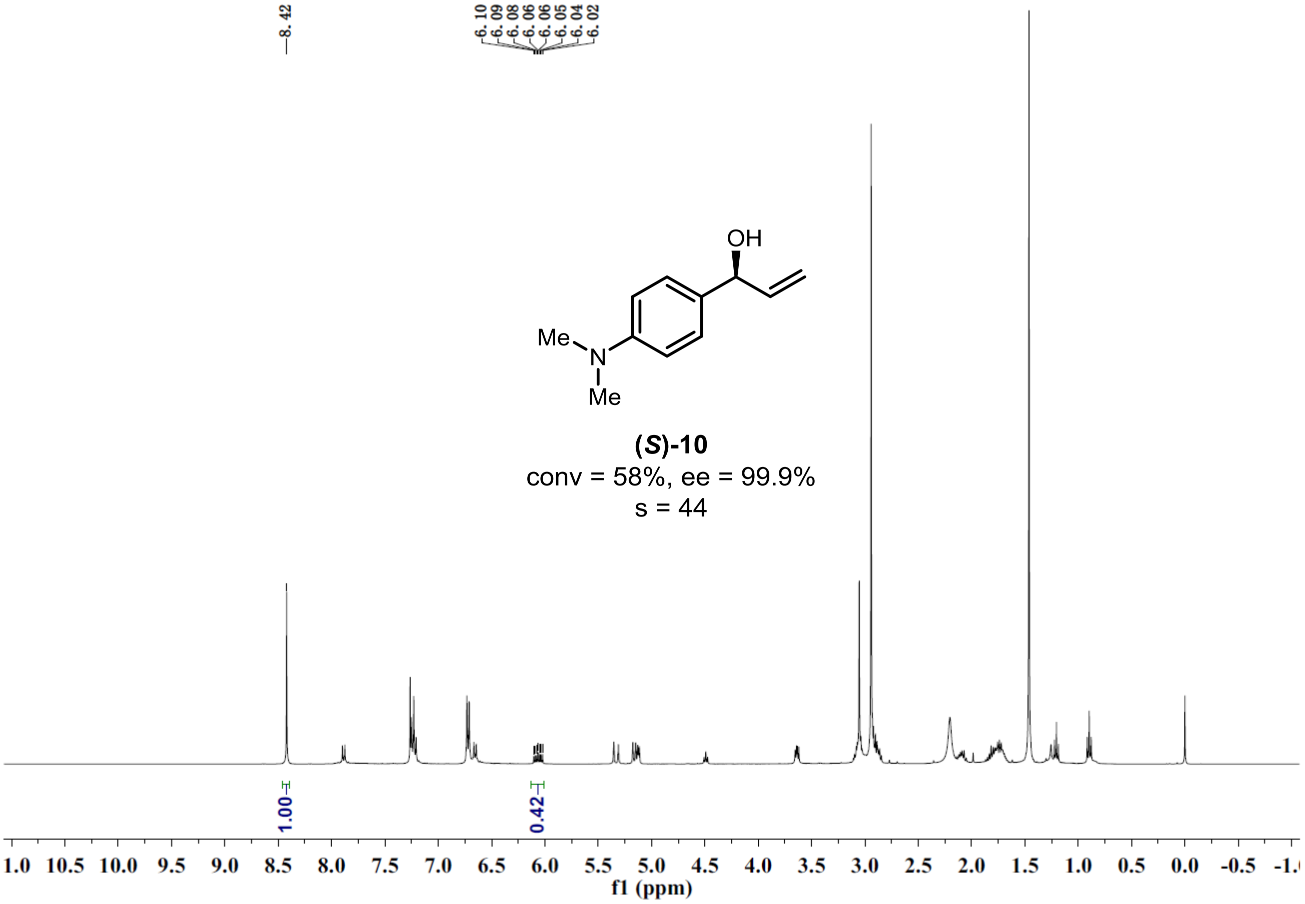

HPLC (AD-H, 0.46*25 cm, 5µm, hexane/isopropanol = 95/5, flow 1 mL/min, detection at 210 nm) retention time = 15.790 min (minor) and 16.525 min (major).

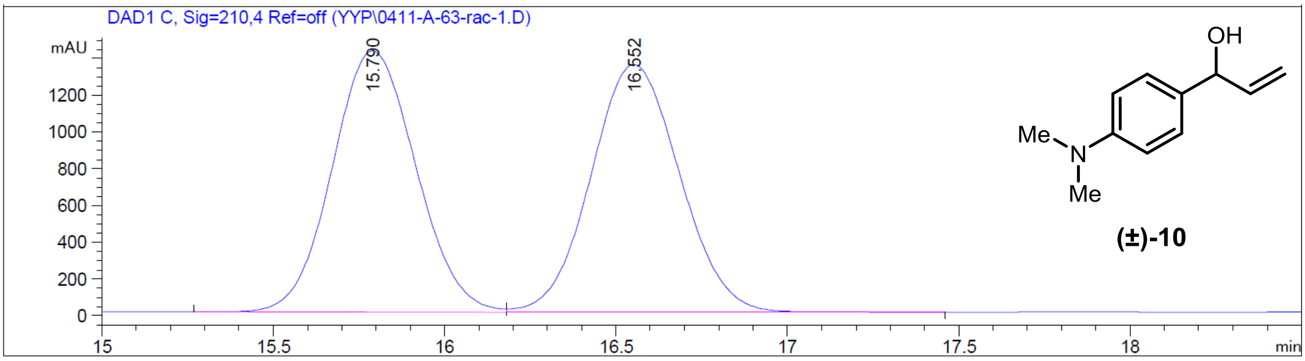

Signal 2: DAD1 C, Sig=210,4 Ref=off

Peak #	RetTime [min]	Type	Width [min]	Area [mAU*s]	Height [mAU]	Area %
1	15.790	BV	0.2660	2.41288e4	1421.91504	49.8937
2	16.552	VB	0.2819	2.42316e4	1346.75793	50.1063

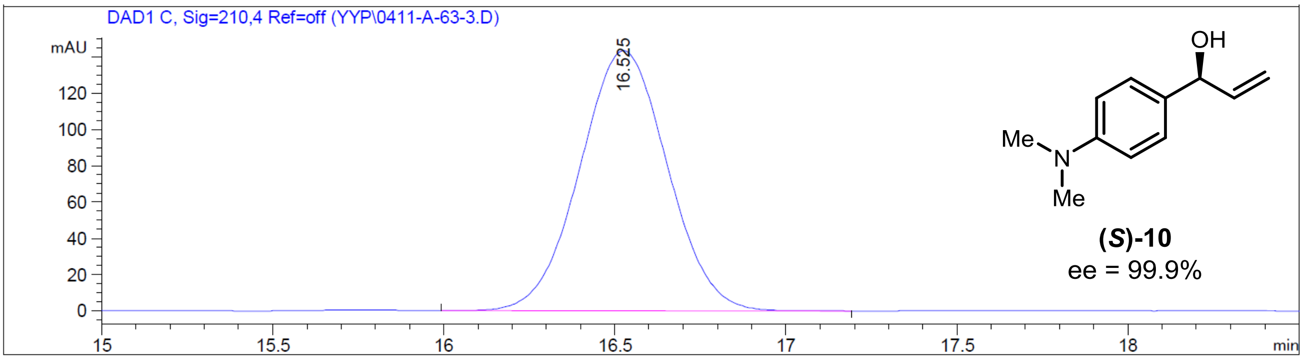

Signal 2: DAD1 C, Sig=210,4 Ref=off

Peak #	RetTime [min]	Type	Width [min]	Area [mAU*s]	Height [mAU]	Area %
1	16.525	BB	0.2782	2559.06909	143.42567	100.0000

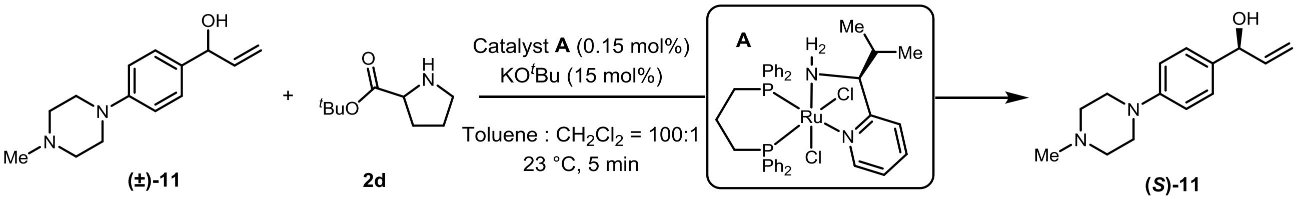

The general procedure **II** was followed. The conversion of **(±)-11** was determined by crude ^{1}H NMR (56% conversion, 44% yield, 99.9% ee, *s* = 60).

Crude ^{1}H NMR

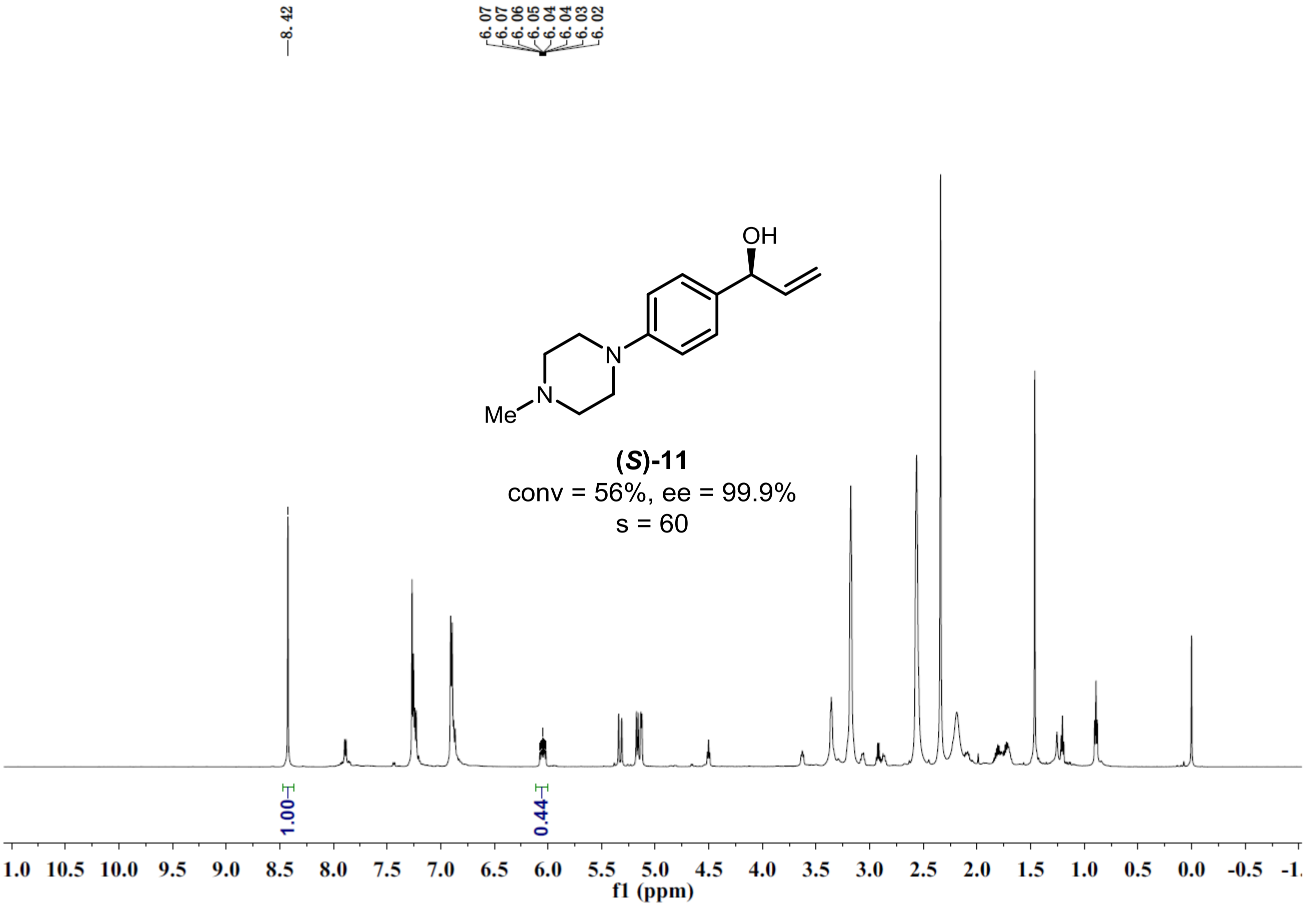

HPLC (OJ-H, 0.46*25 cm, 5μm, hexane/isopropanol = 90/10, flow 1 mL/min, detection at 210 nm) retention time = 24.022 min (major) and 31.224 min (minor).

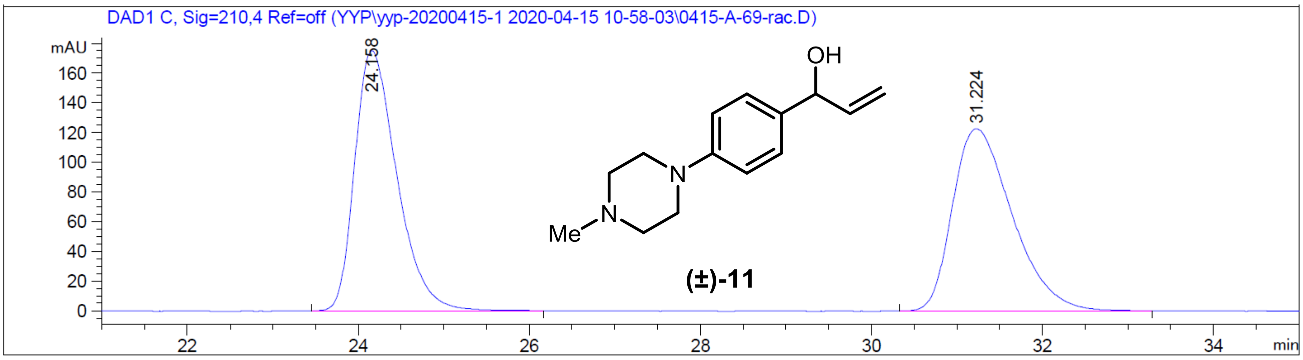

Signal 2: DAD1 C, Sig=210,4 Ref=off

Peak #	RetTime [min]	Type	Width [min]	Area [mAU*s]	Height [mAU]	Area %
1	24.158	BB	0.5276	5991.46094	174.91461	50.1771
2	31.224	BB	0.7442	5949.16455	122.58590	49.8229

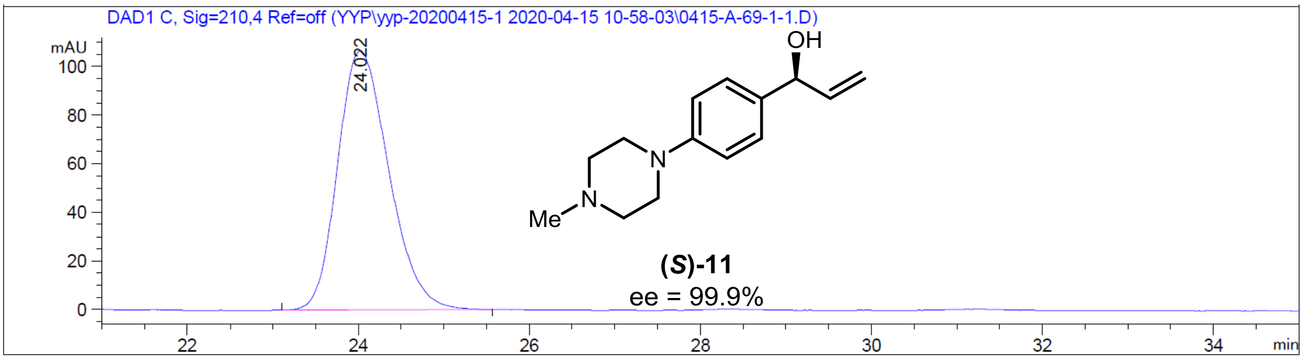

Signal 2: DAD1 C, Sig=210,4 Ref=off

Peak #	RetTime [min]	Type	Width [min]	Area [mAU*s]	Height [mAU]	Area %
1	24.022	BB	0.6446	4428.11523	106.60508	100.0000

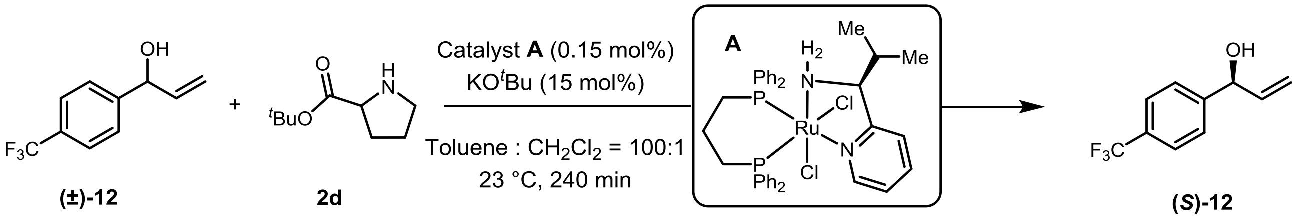

The general procedure **II** was followed. The conversion of **(±)-12** was determined by crude ^{1}H NMR (64% conversion, 36% yield, 97% ee, *s* = 13).

Crude ^{1}**H NMR**

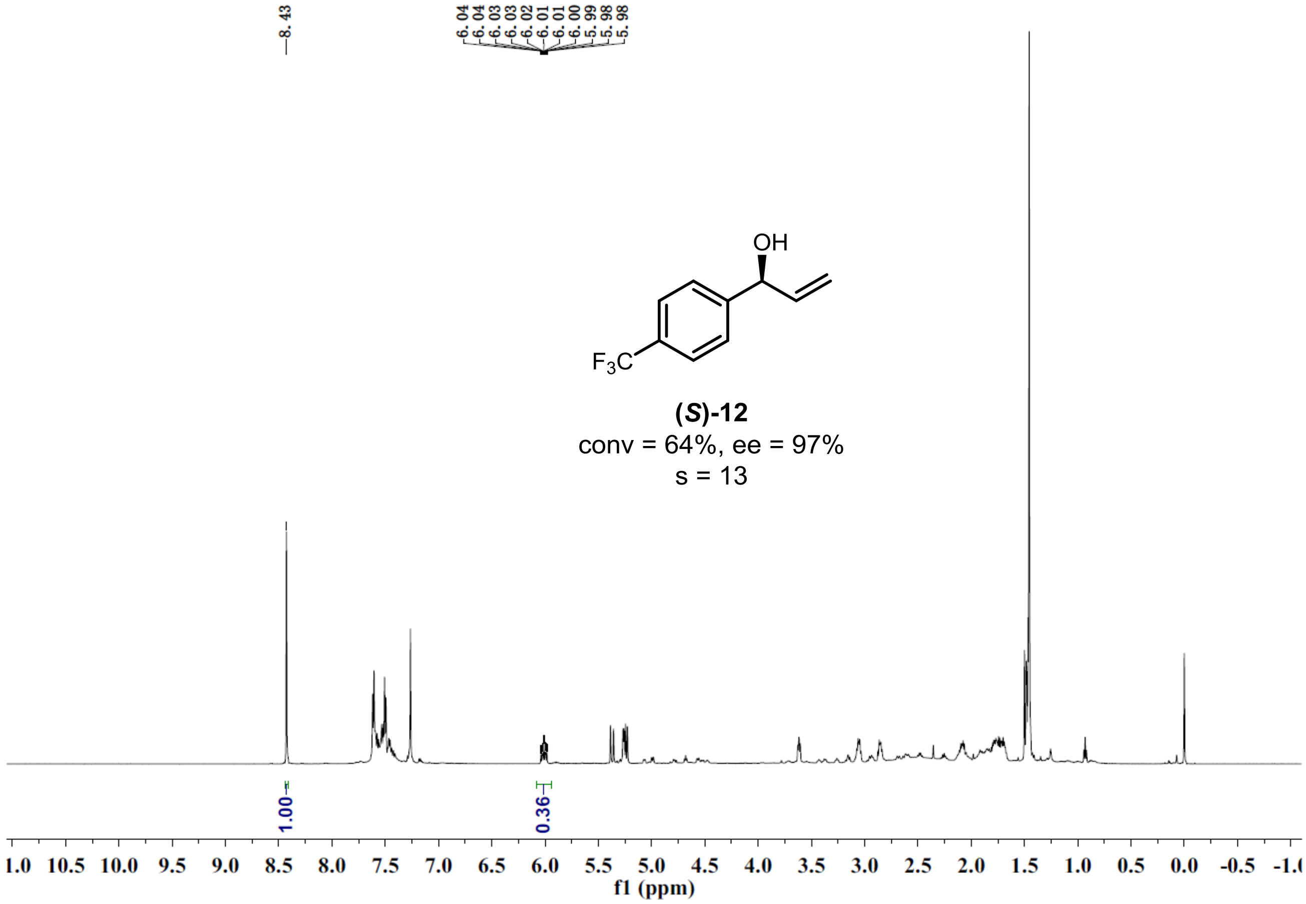

HPLC (AD-H, 0.46*25 cm, 5μm, hexane/isopropanol = 98/2, flow 1 mL/min, detection at 210 nm) retention time = 13.448 min (minor) and 14.891 min (major).

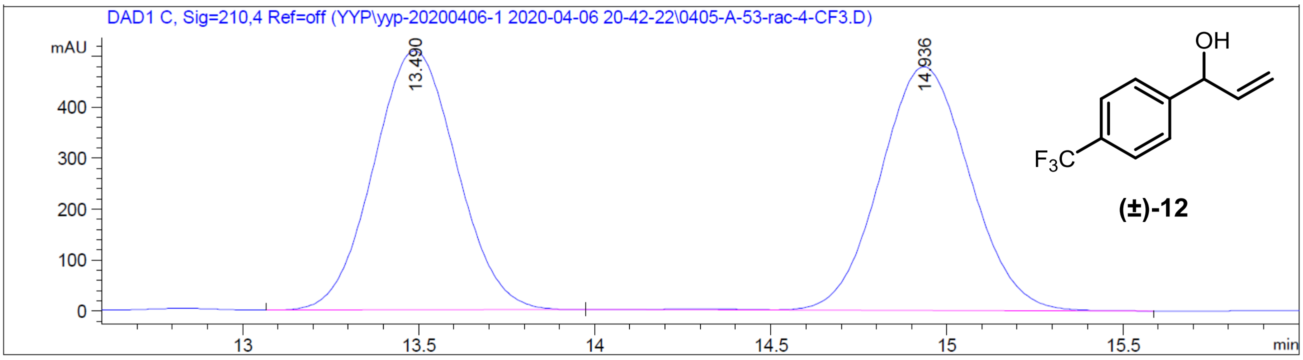

Signal 2: DAD1 C, Sig=210,4 Ref=off

Peak #	RetTime [min]	Type	Width [min]	Area [mAU*s]	Height [mAU]	Area %
1	13.490	BB	0.2579	8379.86914	509.42563	49.6604
2	14.936	VB R	0.2762	8494.47949	478.54047	50.3396

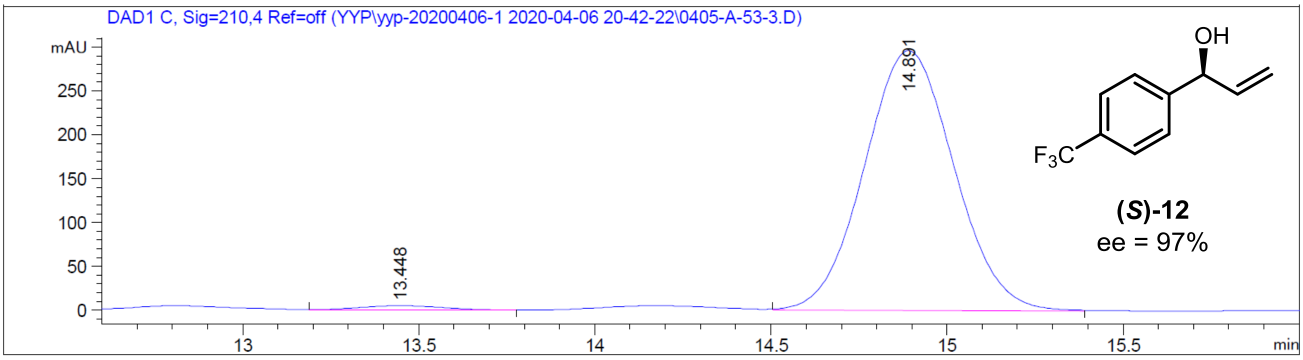

Signal 2: DAD1 C, Sig=210,4 Ref=off

Peak #	RetTime [min]	Type	Width [min]	Area [mAU*s]	Height [mAU]	Area %
1	13.448	MM R	0.2693	77.92544	4.82246	1.4601
2	14.891	MM R	0.2958	5259.23486	296.33502	98.5399

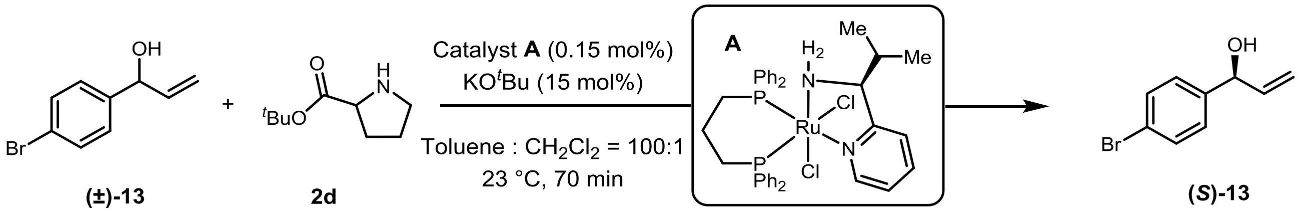

The general procedure **II** was followed. The conversion of **(±)-13** was determined by crude ^{1}H NMR (65% conversion, 35% yield, 98% ee, *s* = 14).

Crude **^{1}H NMR**

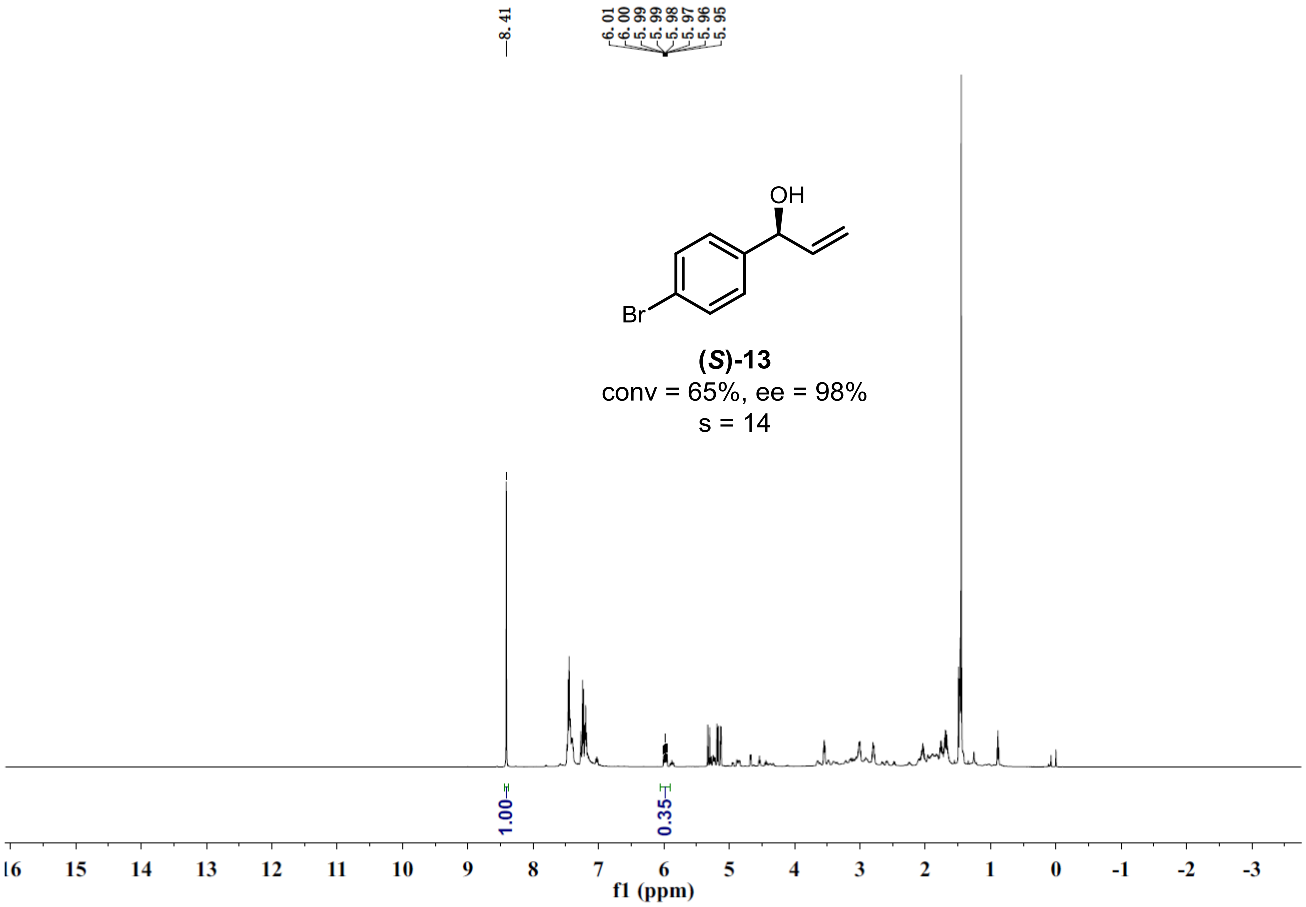

HPLC (OJ-H, 0.46*25 cm, 5µm, hexane/isopropanol = 95/5, flow 1 mL/min, detection at 210 nm) retention time = 12.745 min (major) and 13.449 min (minor).

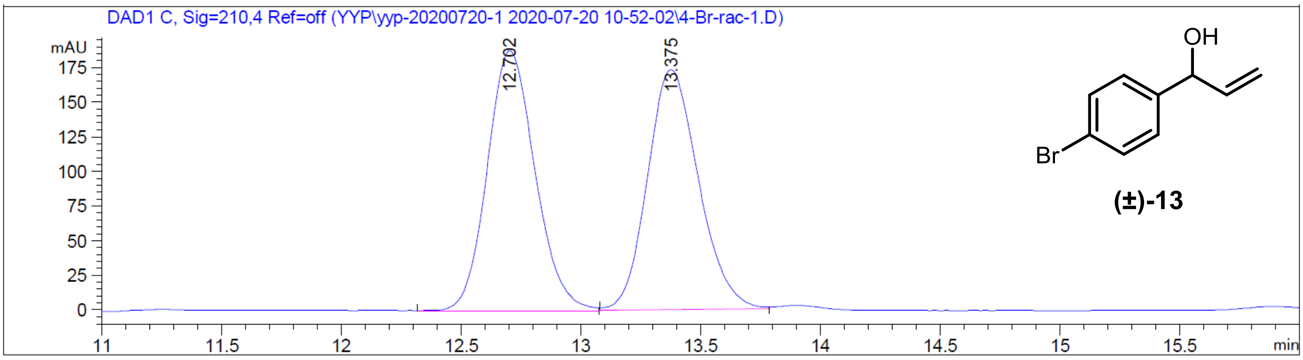

Signal 2: DAD1 C, Sig=210,4 Ref=off

Peak #	RetTime [min]	Type	Width [min]	Area [mAU*s]	Height [mAU]	Area %
1	12.702	BV	0.2138	2602.08398	187.66406	50.3832
2	13.375	MM R	0.2467	2562.50562	173.10497	49.6168

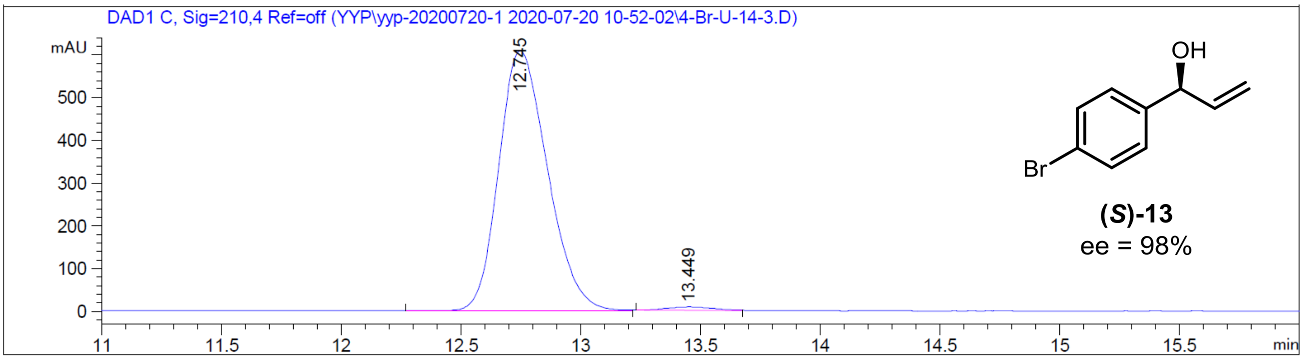

Signal 2: DAD1 C, Sig=210,4 Ref=off

Peak #	RetTime [min]	Type	Width [min]	Area [mAU*s]	Height [mAU]	Area %
1	12.745	MM R	0.2355	8586.74805	607.75439	98.7875
2	13.449	MM R	0.2257	105.38869	7.78128	1.2125

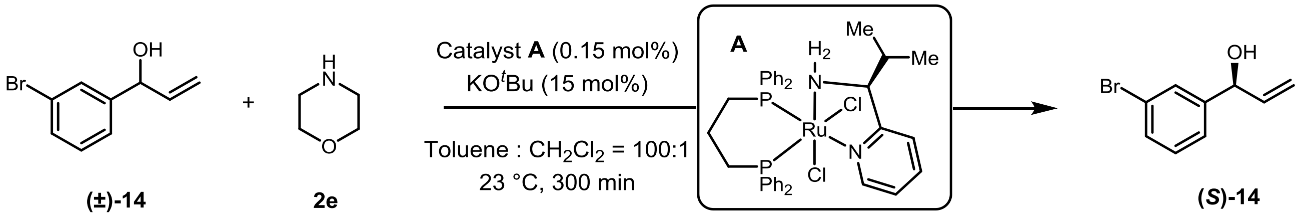

The general procedure **II** was followed. The conversion of **(±)-14** was determined by crude ^{1}H NMR (63% conversion, 37% yield, 97% ee, *s* = 14).

Crude **^{1}H NMR**

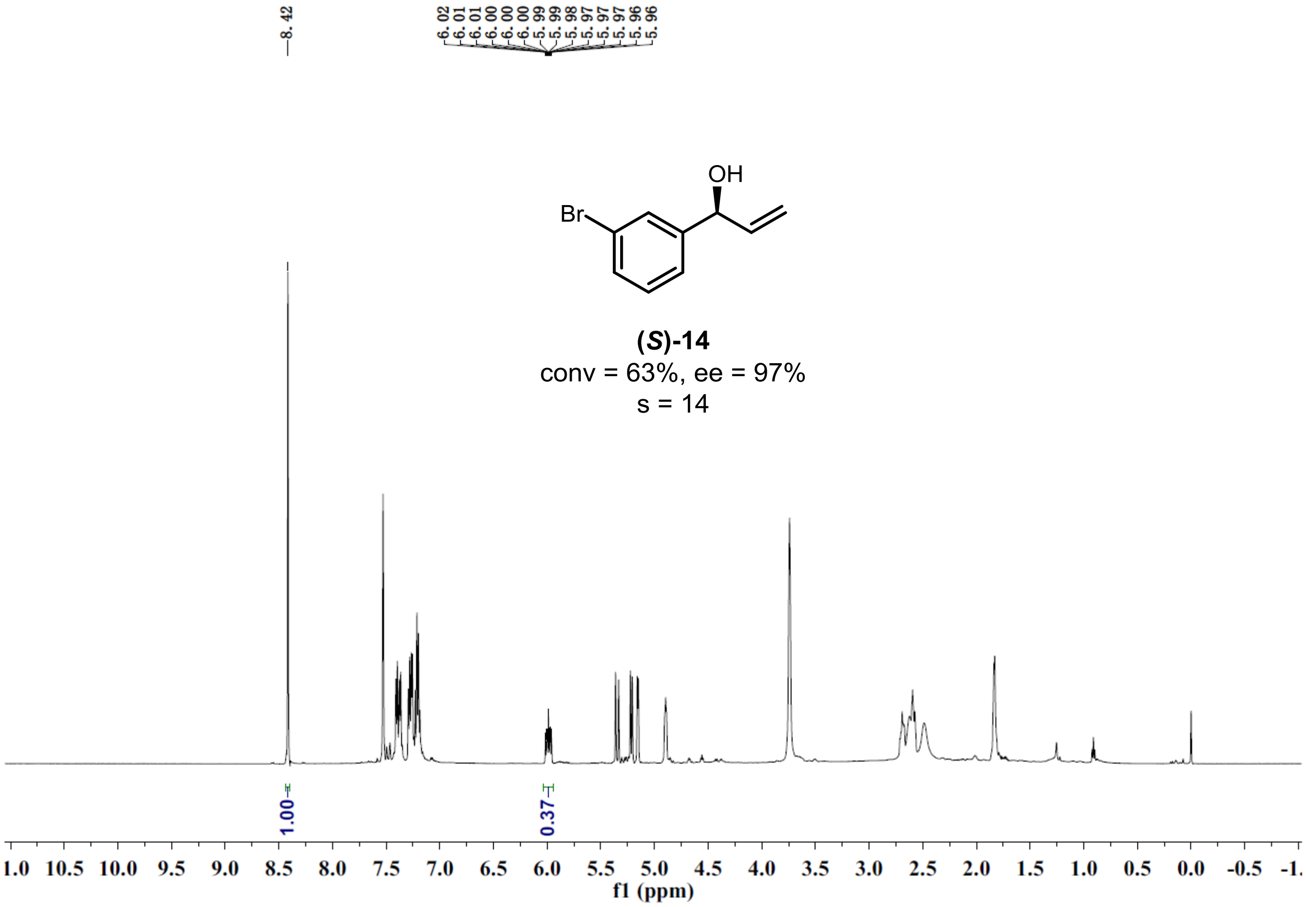

HPLC (OJ-H, 0.46*25 cm, 5μm, hexane/isopropanol = 95/5, flow 1 mL/min, detection at 210 nm) retention time = 11.708 min (major) and 12.604 min (minor).

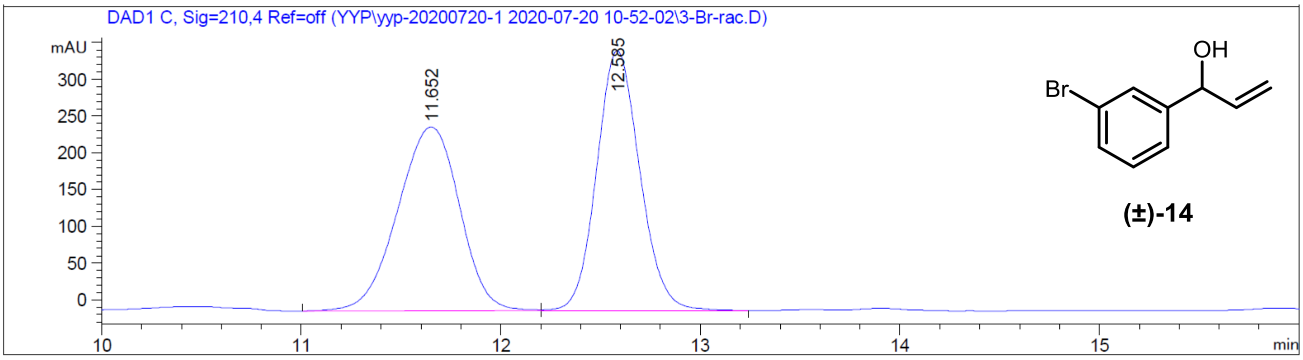

Signal 2: DAD1 C, Sig=210,4 Ref=off

Peak #	RetTime [min]	Type	Width [min]	Area [mAU*s]	Height [mAU]	Area %
1	11.652	BV	0.3392	5388.73584	250.24437	50.3309
2	12.585	VB	0.2313	5317.88086	353.99438	49.6691

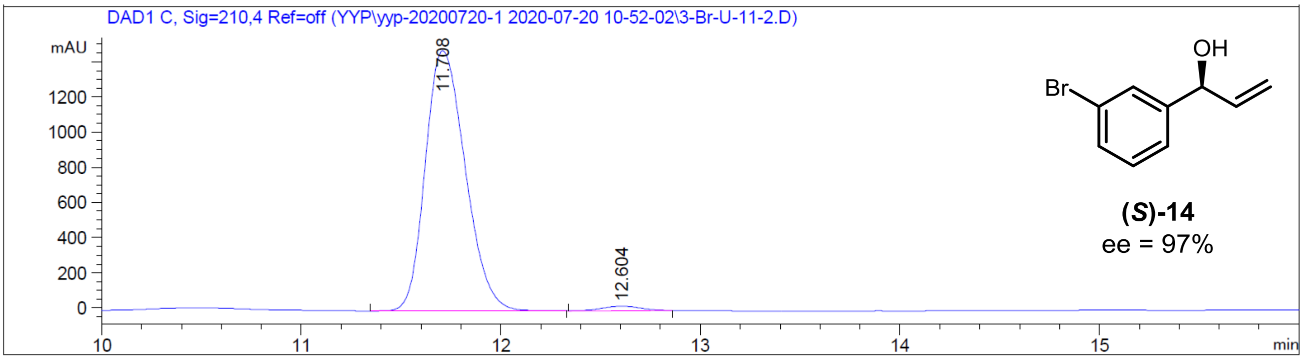

Signal 2: DAD1 C, Sig=210,4 Ref=off

Peak #	RetTime [min]	Type	Width [min]	Area [mAU*s]	Height [mAU]	Area %
1	11.708	BV	0.2183	2.05961e4	1480.60217	98.3427
2	12.604	MM R	0.2240	347.09787	25.82184	1.6573

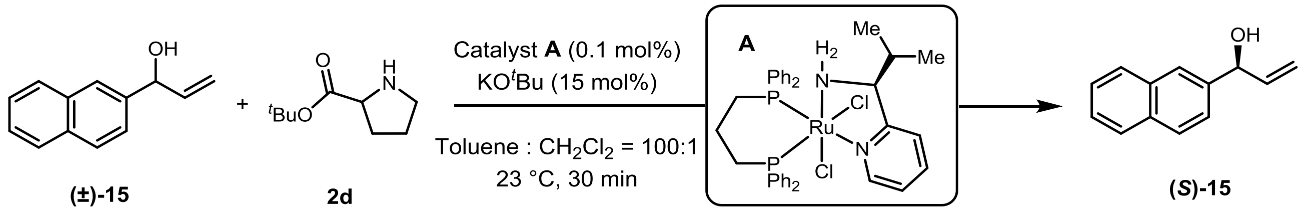

The general procedure **II** was followed. The conversion of **(±)-15** was determined by crude ^{1}H NMR (60% conversion, 40% yield, 96% ee, *s* = 17).

Crude **^{1}H NMR**

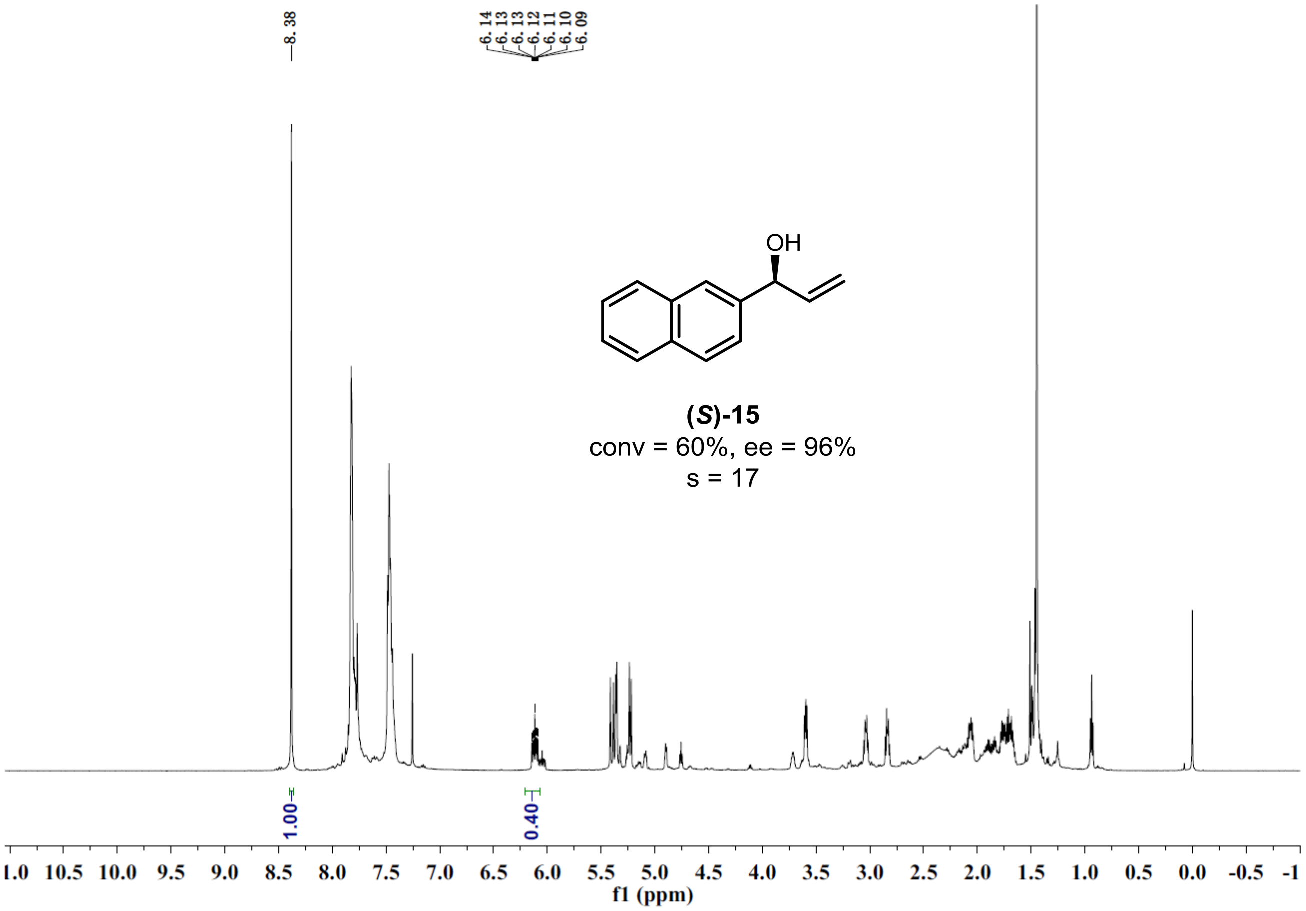

HPLC (AS-H, 0.46*25 cm, 5μm, hexane/isopropanol = 99/1, flow 1 mL/min, detection at 210 nm) retention time = 27.901 min (minor) and 31.993 min (major).

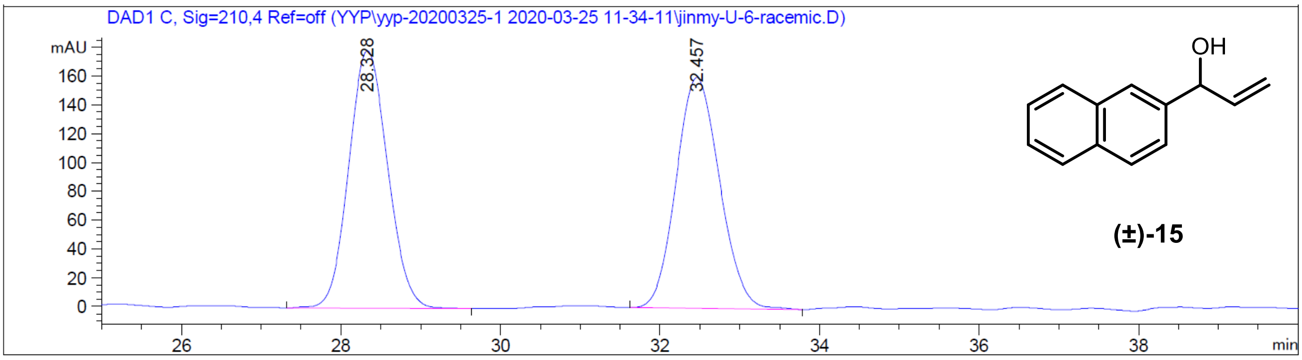

Signal 2: DAD1 C, Sig=210,4 Ref=off

Peak #	RetTime [min]	Type	Width [min]	Area [mAU*s]	Height [mAU]	Area %
1	28.328	BB	0.5296	6057.02979	178.63304	49.9687
2	32.457	BB	0.5918	6064.61328	160.13579	50.0313

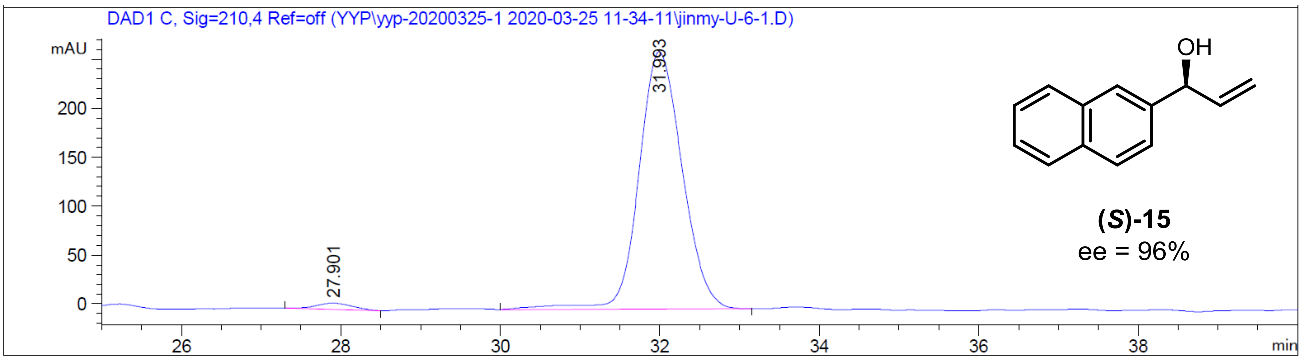

Signal 2: DAD1 C, Sig=210,4 Ref=off

Peak #	RetTime [min]	Type	Width [min]	Area [mAU*s]	Height [mAU]	Area %
1	27.901	BB	0.4512	204.79736	6.47173	2.0158
2	31.993	BB	0.5893	9954.92578	263.16553	97.9842

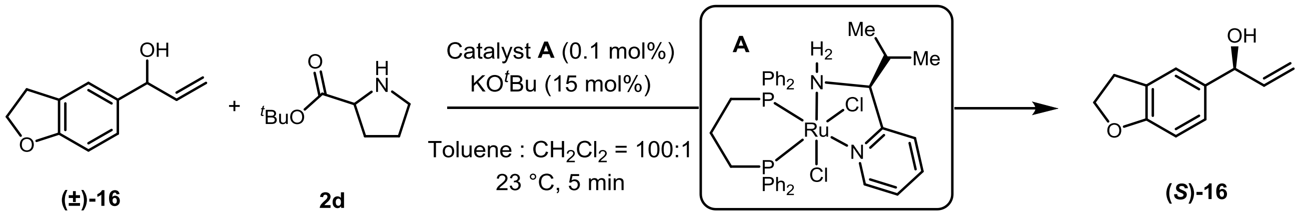

The general procedure **II** was followed. The conversion of **(±)-16** was determined by crude ^{1}H NMR (60% conversion, 40% yield, 96% ee, *s* = 17).

Crude **^{1}H NMR**

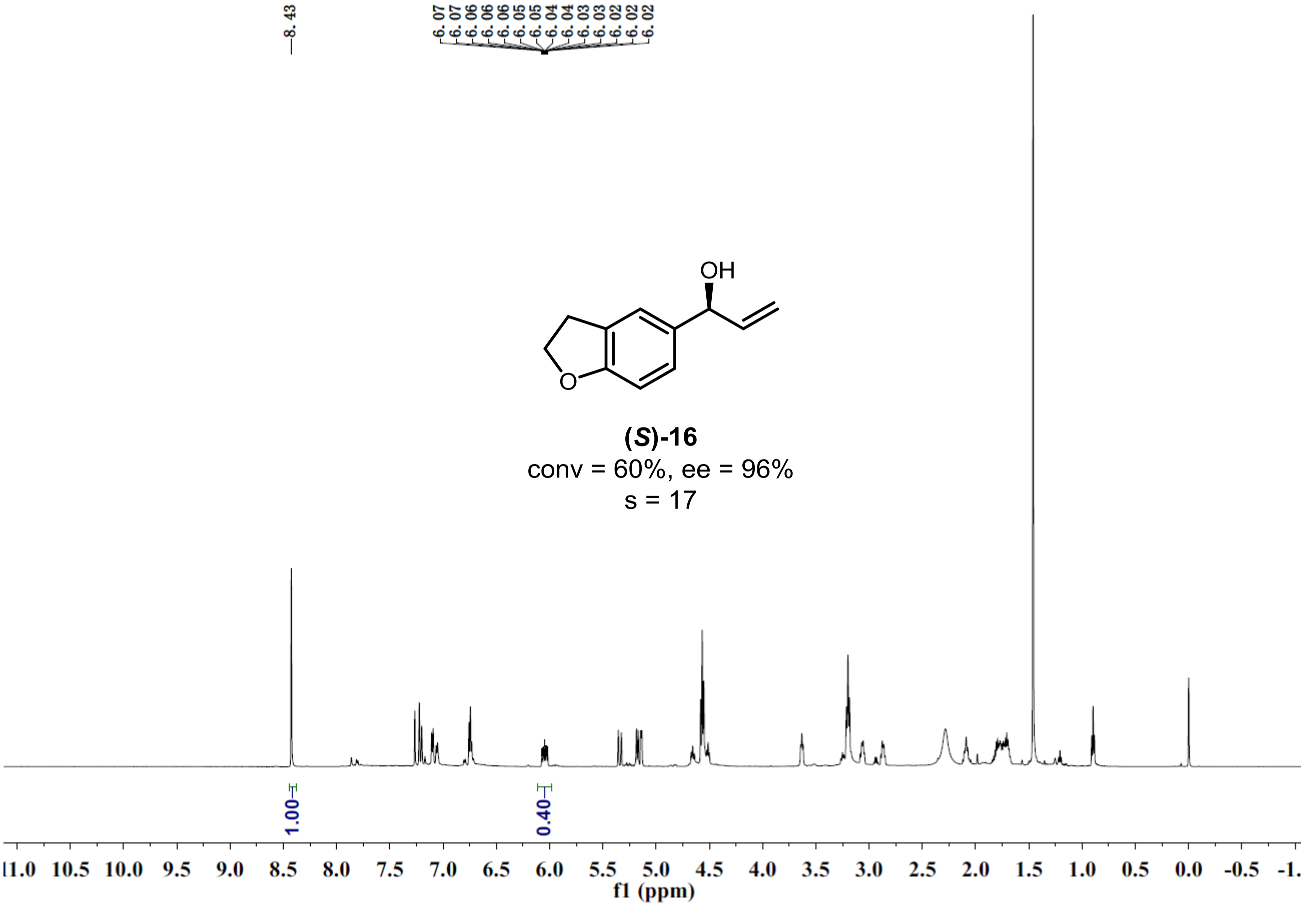

HPLC (AD-H, 0.46*25 cm, 5μm, hexane/isopropanol = 95/5, flow 1 mL/min, detection at 210 nm) retention time = 15.831 min (minor) and 16.999 min (major).

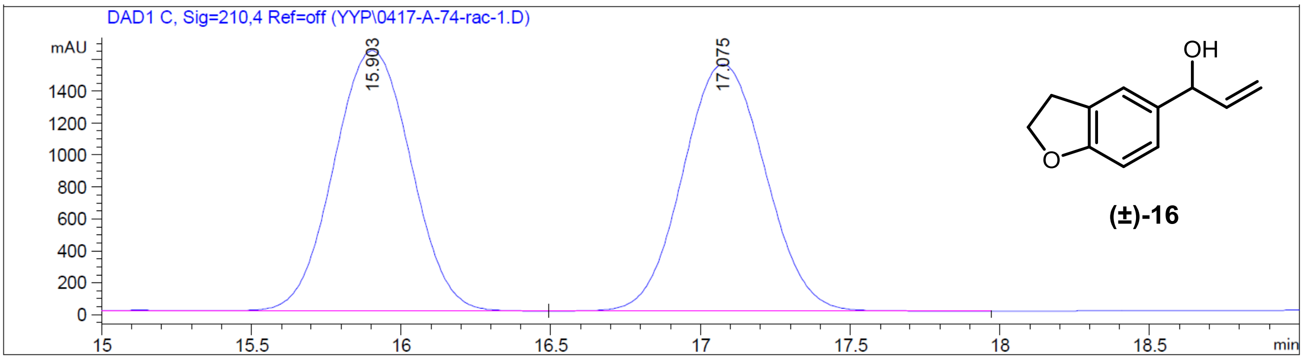

Signal 2: DAD1 C, Sig=210,4 Ref=off

Peak #	RetTime [min]	Type	Width [min]	Area [mAU*s]	Height [mAU]	Area %
1	15.903	VB R	0.2853	2.94356e4	1630.55933	49.8454
2	17.075	BB	0.3035	2.96182e4	1545.94629	50.1546

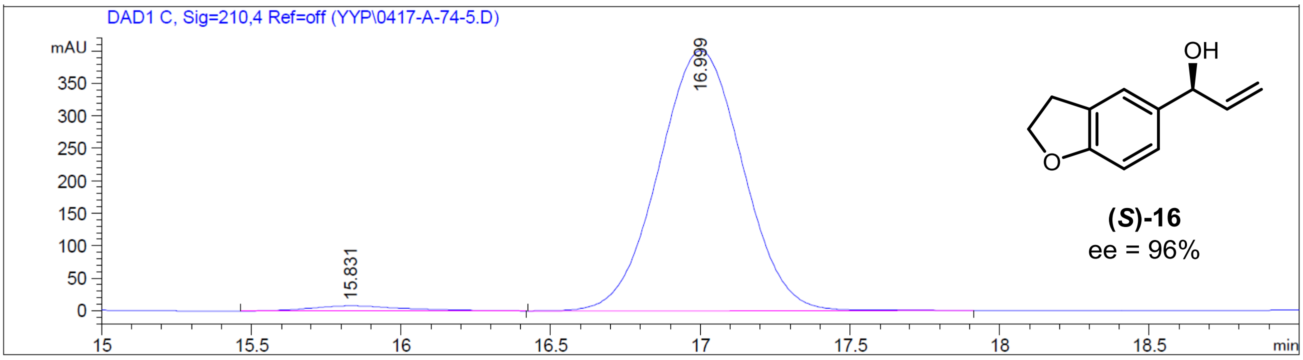

Signal 2: DAD1 C, Sig=210,4 Ref=off

Peak #	RetTime [min]	Type	Width [min]	Area [mAU*s]	Height [mAU]	Area %
1	15.831	BB	0.3180	169.92874	7.86612	2.1257
2	16.999	BB	0.3057	7824.15723	400.88120	97.8743

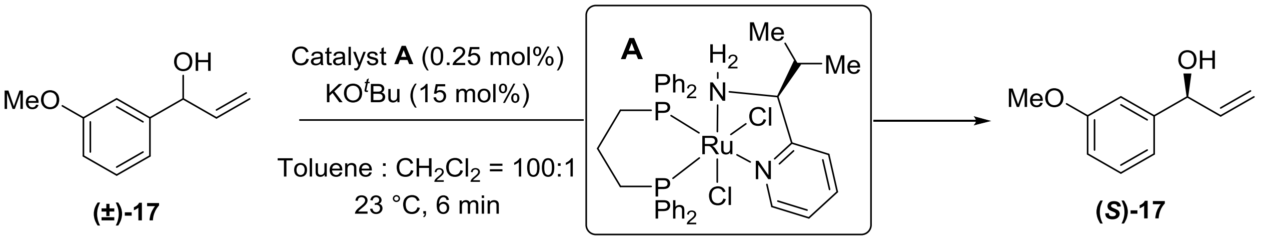

The general procedure **II** was followed. The conversion of **(±)-17** was determined by crude ^{1}H NMR (58% conversion, 42% yield, 82% ee, *s* = 10).

Crude **^{1}H NMR:**

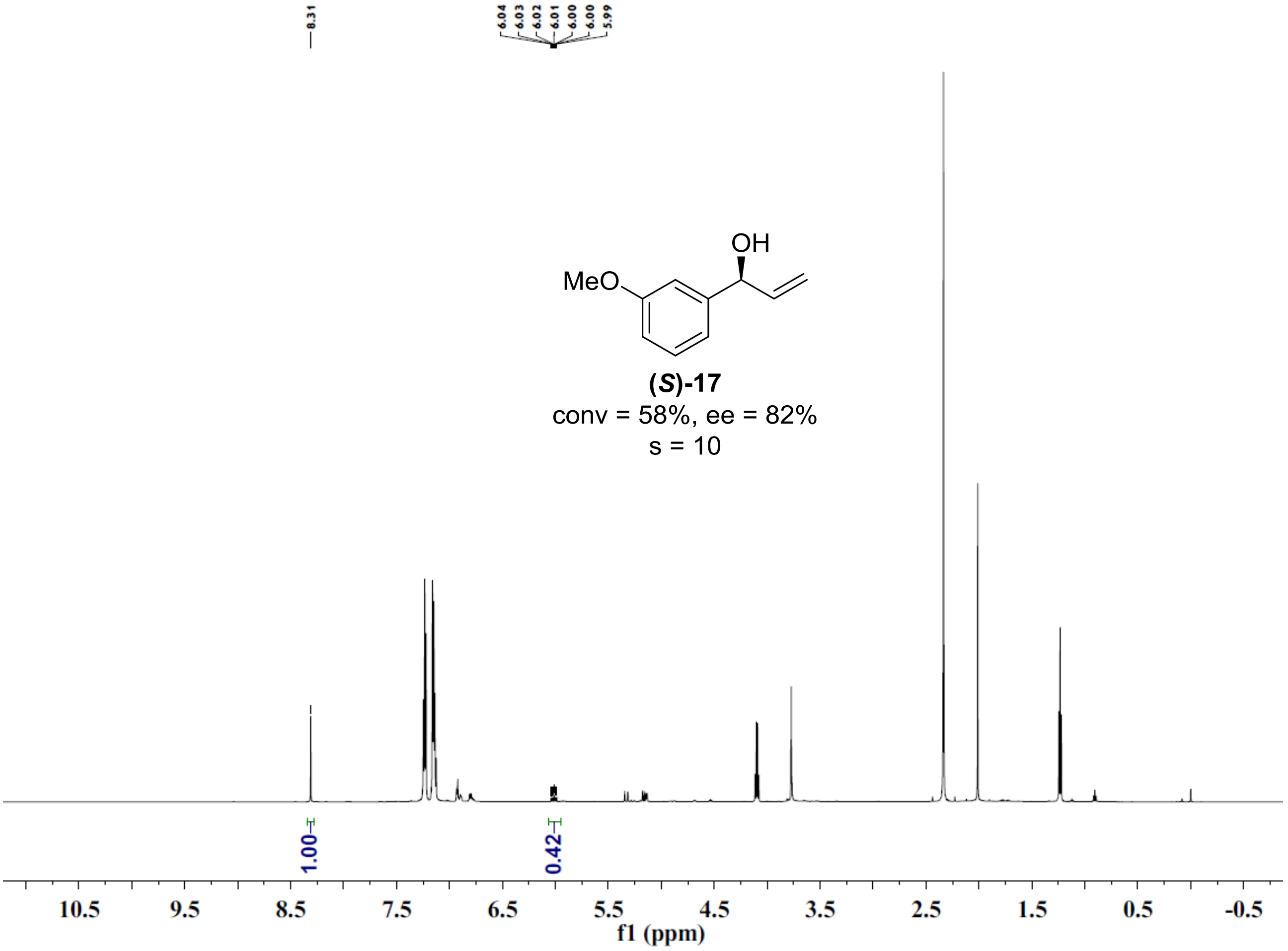

HPLC (OD-H, 0.46*25 cm, 5μm, hexane/isopropanol = 95/5, flow = 1.0 mL/min, detection at 210 nm) retention time = 14.090 min (minor) and 16.574 min (major).

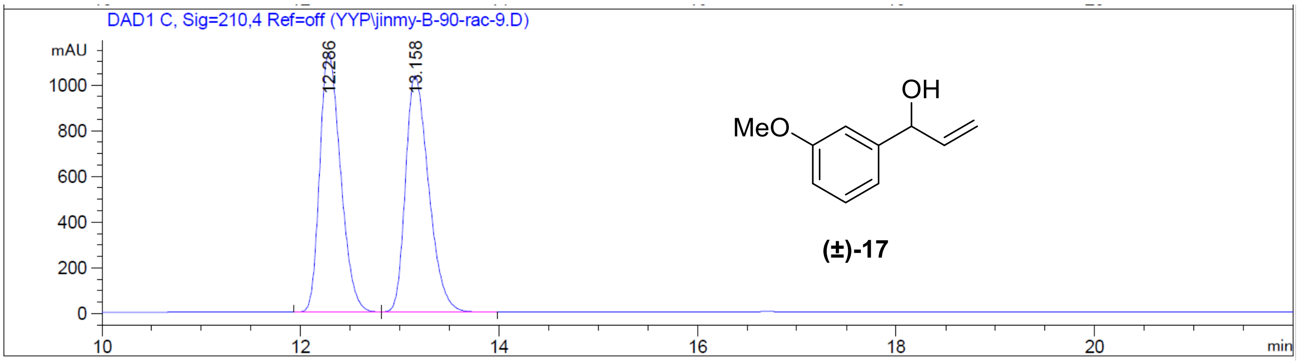

Signal 2: DAD1 C, Sig=210,4 Ref=off

Peak #	RetTime [min]	Type	Width [min]	Area [mAU*s]	Height [mAU]	Area %
1	12.286	BB	0.2262	1.65125e4	1131.63770	49.6992
2	13.158	BB	0.2511	1.67124e4	1031.15894	50.3008

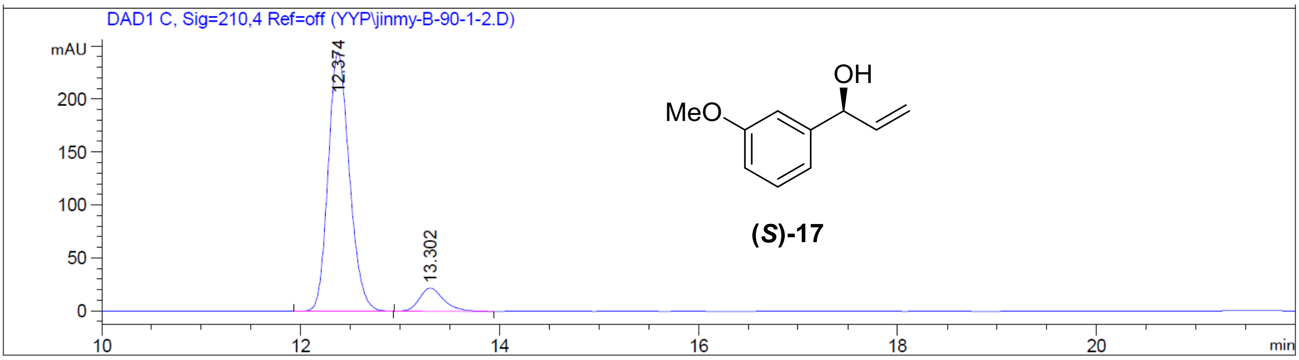

Signal 2: DAD1 C, Sig=210,4 Ref=off

Peak #	RetTime [min]	Type	Width [min]	Area [mAU*s]	Height [mAU]	Area %
1	12.374	BB	0.2286	3617.30249	244.53305	90.7365
2	13.302	BB	0.2542	369.30026	21.95518	9.2635

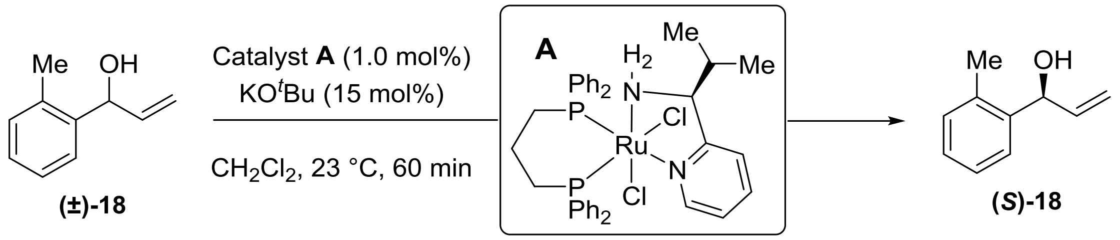

The general procedure **II** was followed. The conversion of **(±)-18** was determined by crude ^{1}H NMR (60% conversion, 40% yield, 90% ee, *s* = 12)

Crude ^{1}**H NMR:**

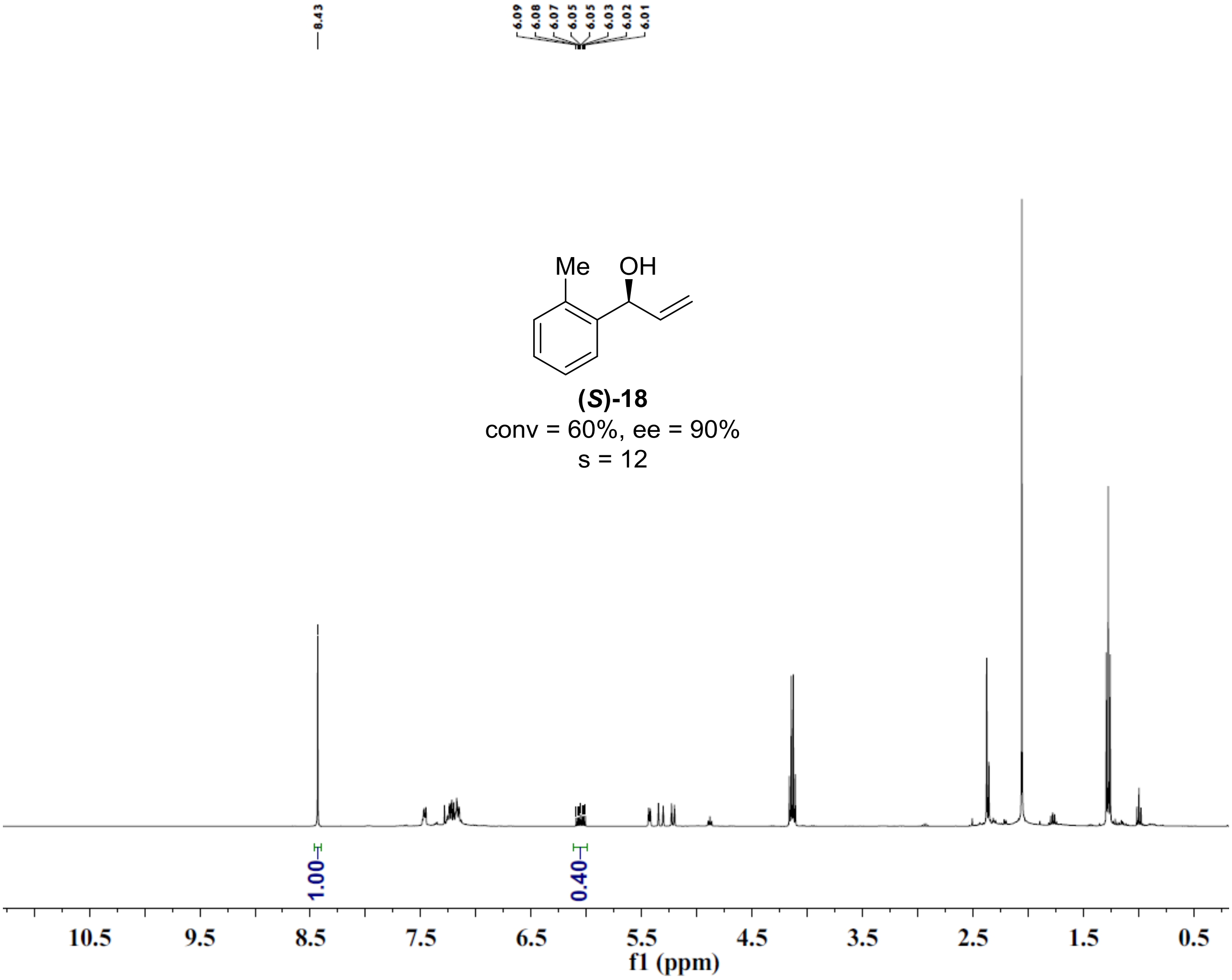

HPLC (OD-H, 0.46*25 cm, 5μm, hexane/isopropanol = 95/5, flow = 1.0 mL/min, detection at 210 nm) retention time = 15.215 min (minor) and 16.513 min (major).

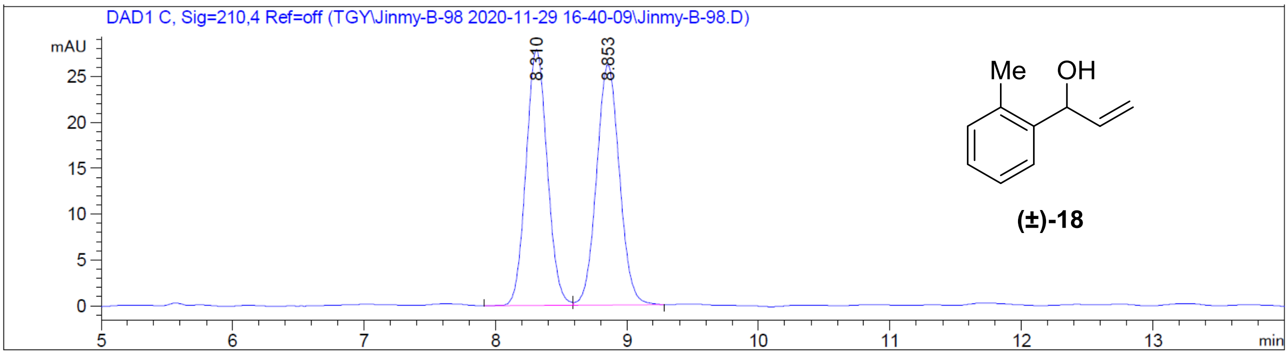

Signal 2: DAD1 C, Sig=210,4 Ref=off

Peak #	RetTime [min]	Type	Width [min]	Area [mAU*s]	Height [mAU]	Area %
1	8.310	BV	0.1751	313.70532	27.92222	49.6774
2	8.853	VB	0.1873	317.77905	26.23969	50.3226

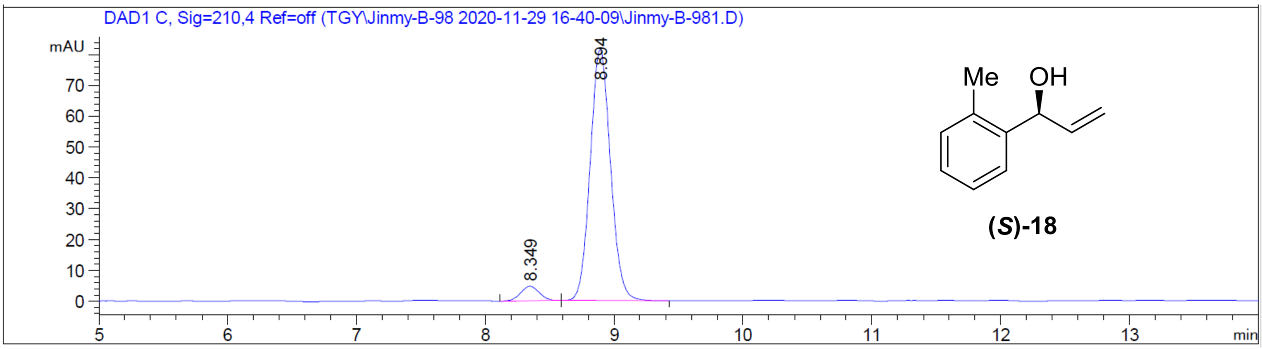

Signal 2: DAD1 C, Sig=210,4 Ref=off

Peak #	RetTime [min]	Type	Width [min]	Area [mAU*s]	Height [mAU]	Area %
1	8.349	BB	0.1567	47.40353	4.73980	5.0254
2	8.894	BB	0.1703	895.88013	81.44602	94.9746

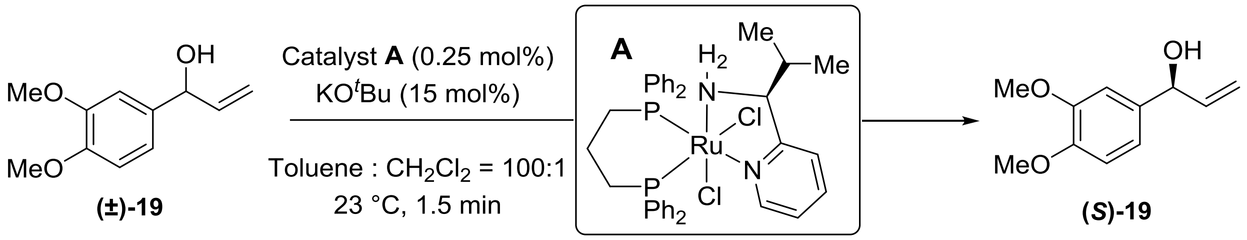

The general procedure **II** was followed. The conversion of **(±)-19** was determined by crude ^{1}H NMR (60% conversion, 40% yield, 94% ee, *s* = 15).

Crude **^{1}H NMR:**

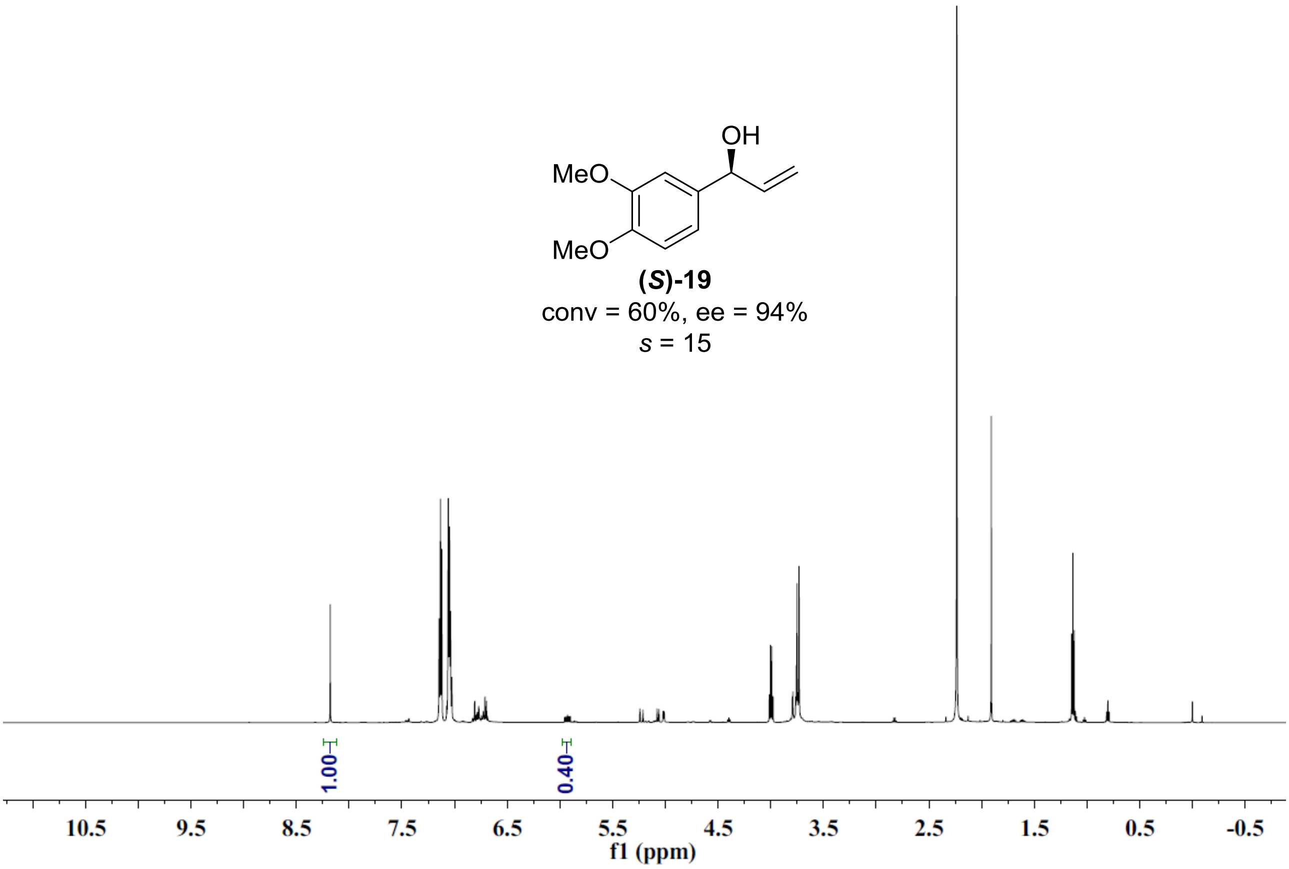

HPLC (OJ-H, 0.46*25 cm, 5μm, hexane/isopropanol = 80/20, flow = 1.0 mL/min, detection at 210 nm) retention time = 15.215 min (minor) and 16.513 min (major).

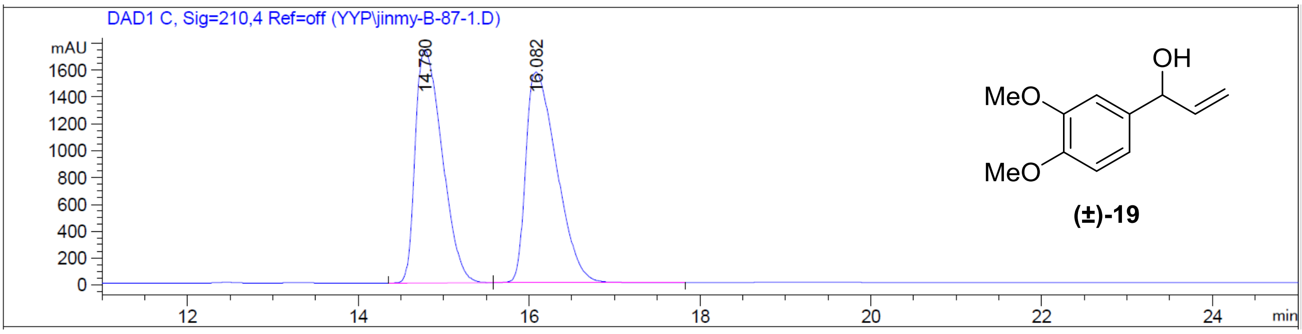

Signal 3: DAD1 C, Sig=210,4 Ref=off

Peak #	RetTime [min]	Type	Width [min]	Area [mAU*s]	Height [mAU]	Area %
1	14.780	BB	0.3468	3.79587e4	1737.87012	49.0718
2	16.082	BB	0.3926	3.93948e4	1570.64282	50.9282

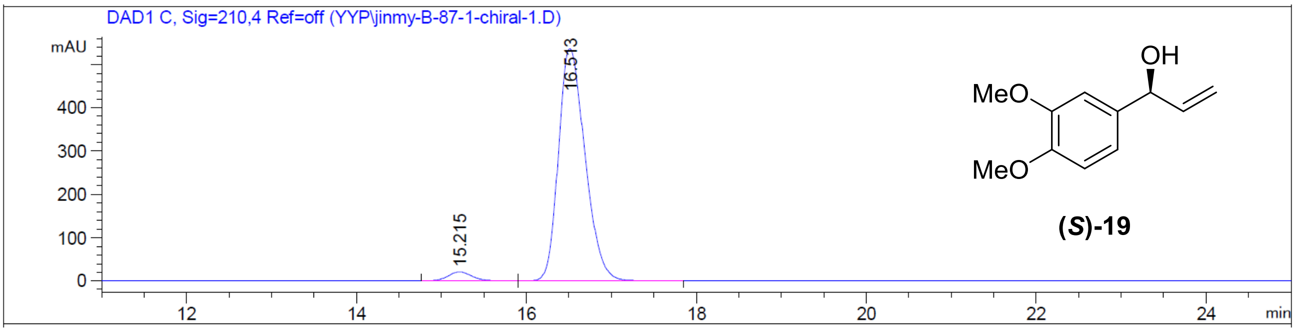

Signal 3: DAD1 C, Sig=210,4 Ref=off

Peak #	RetTime [min]	Type	Width [min]	Area [mAU*s]	Height [mAU]	Area %
1	15.215	BB	0.2923	385.94513	20.43919	3.2164
2	16.513	BB	0.3369	1.16134e4	535.84351	96.7836

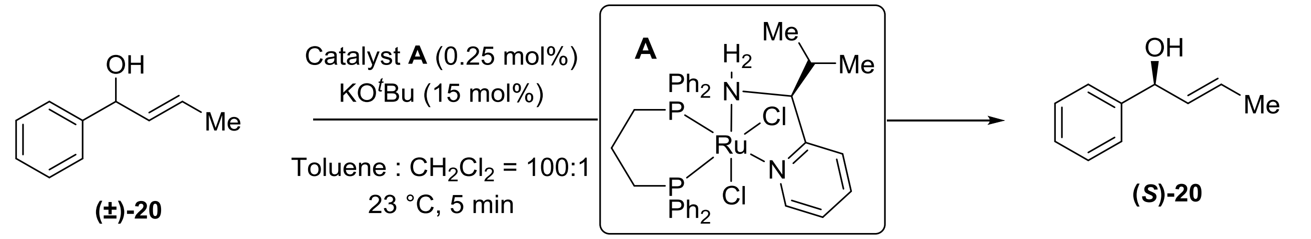

The general procedure **II** was followed. The conversion of **(±)-20** was determined by crude ^{1}H NMR (60% conversion, 40% yield, 97% ee, *s* = 19).

Crude **^{1}H NMR:**

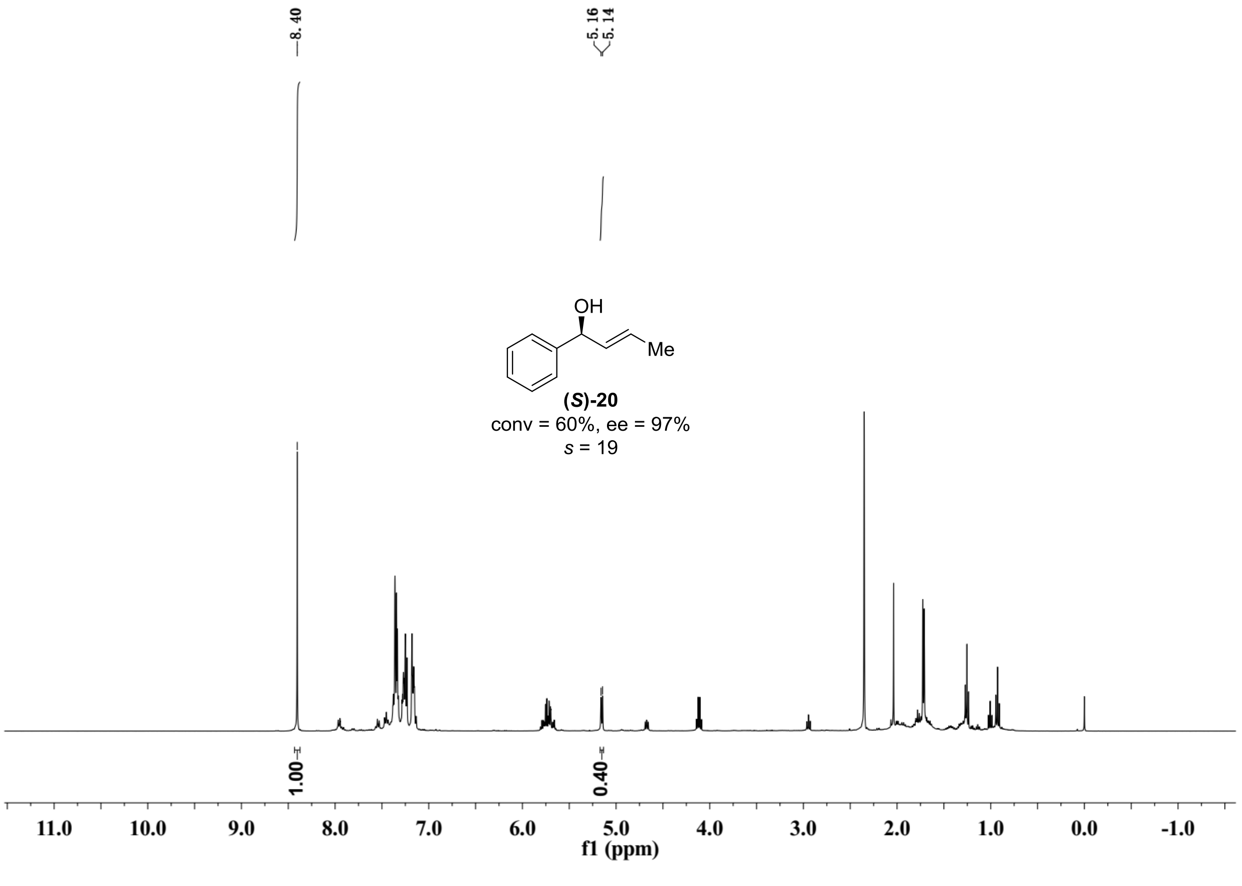

HPLC (OD-H, 0.46*25 cm, 5μm, hexane/isopropanol = 98/2, flow = 1.0 mL/min, detection at 210 nm) retention time = 16.202 min (minor) and 21.157 min (major).

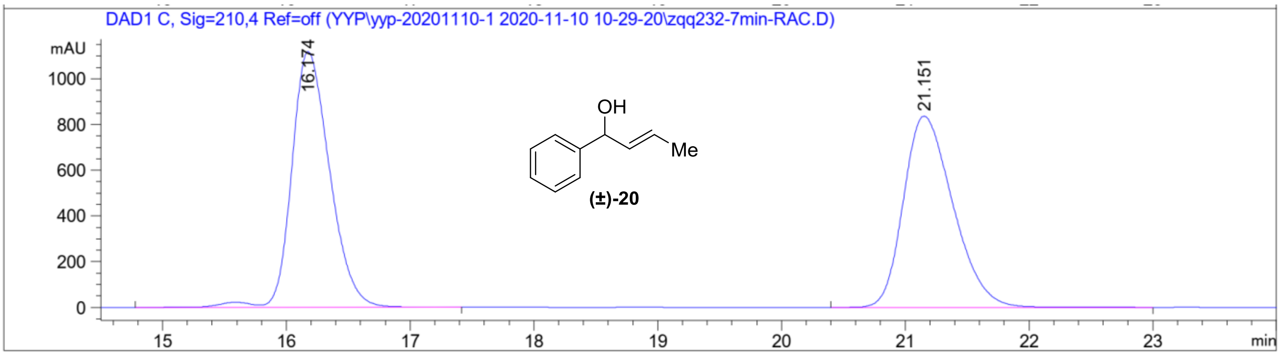

Signal 2: DAD1 C, Sig=210,4 Ref=off

Peak #	RetTime [min]	Type	Width [min]	Area [mAU*s]	Height [mAU]	Area %
1	16.174	VB R	0.3250	2.38853e4	1118.38000	50.6329
2	21.151	BB	0.4327	2.32882e4	836.87445	49.3671

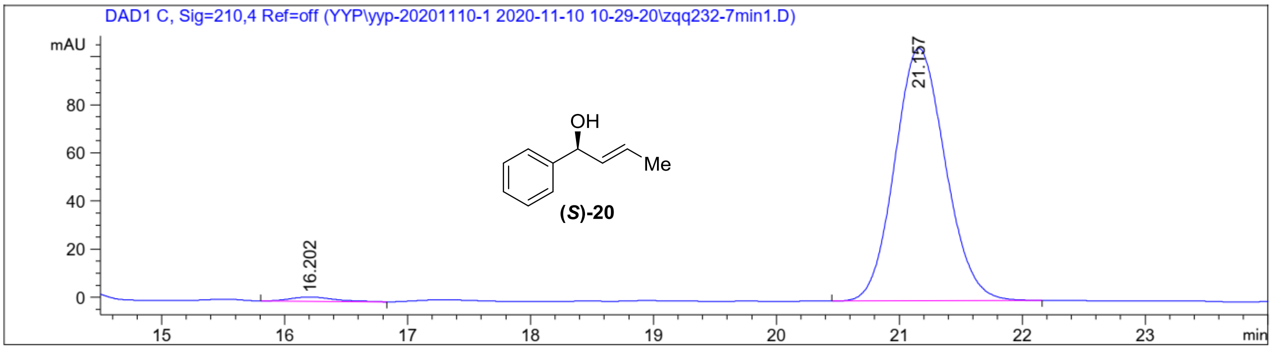

Signal 2: DAD1 C, Sig=210,4 Ref=off

Peak #	RetTime [min]	Type	Width [min]	Area [mAU*s]	Height [mAU]	Area %
1	16.202	BB	0.3549	43.30797	1.74875	1.4236
2	21.157	BB	0.4433	2998.87280	104.98636	98.5764

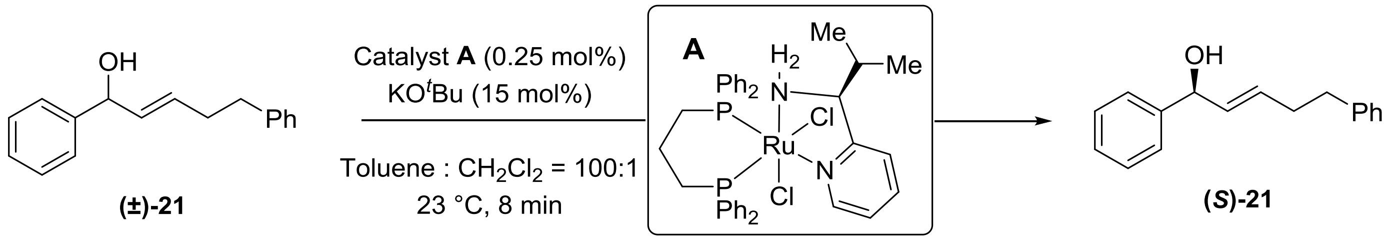

The general procedure **II** was followed. The conversion of **(±)-21** was determined by crude ^{1}H NMR (64% conversion, 36% yield, 94% ee, *s* = 11).

Crude **^{1}H NMR:**

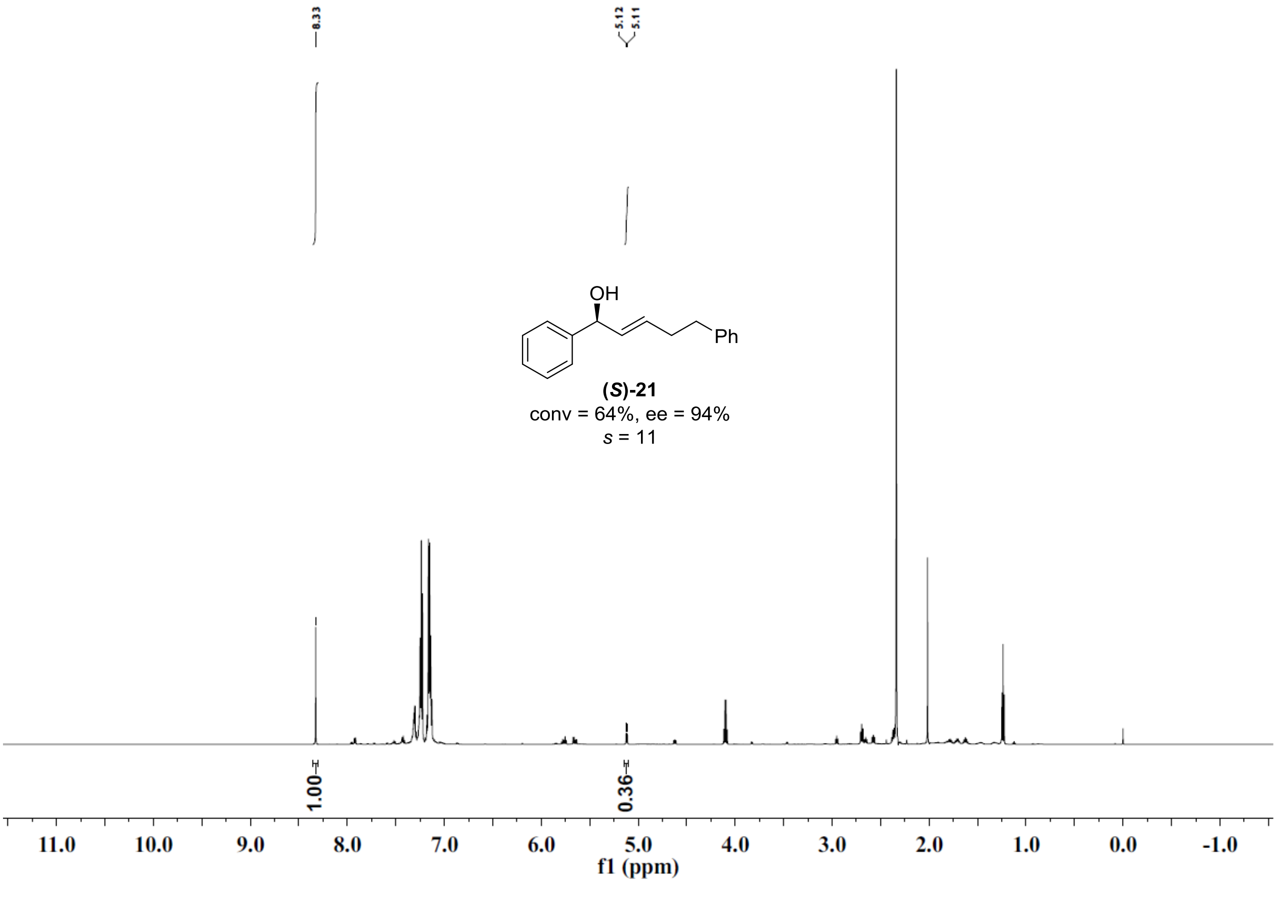

HPLC (OD-H, 0.46*25 cm, 5µm, hexane/ethanol = 95/5, flow = 1.0 mL/min, detection at 210 nm) retention time = 15.318 min (minor) and 16.947 min (major).

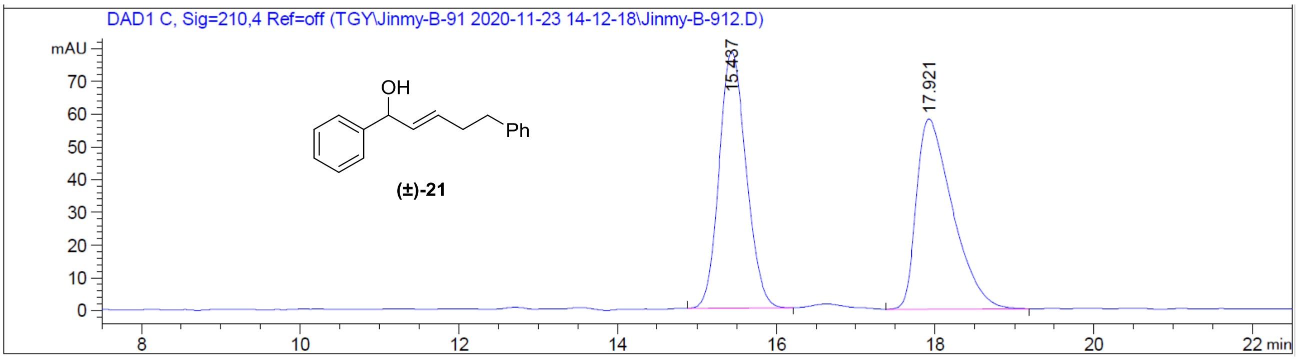

Signal 2: DAD1 C, Sig=210,4 Ref=off

Peak #	RetTime [min]	Type	Width [min]	Area [mAU*s]	Height [mAU]	Area %
1	15.437	BB	0.3654	1848.56592	78.30972	50.1704
2	17.921	BB	0.4811	1836.00720	58.03273	49.8296

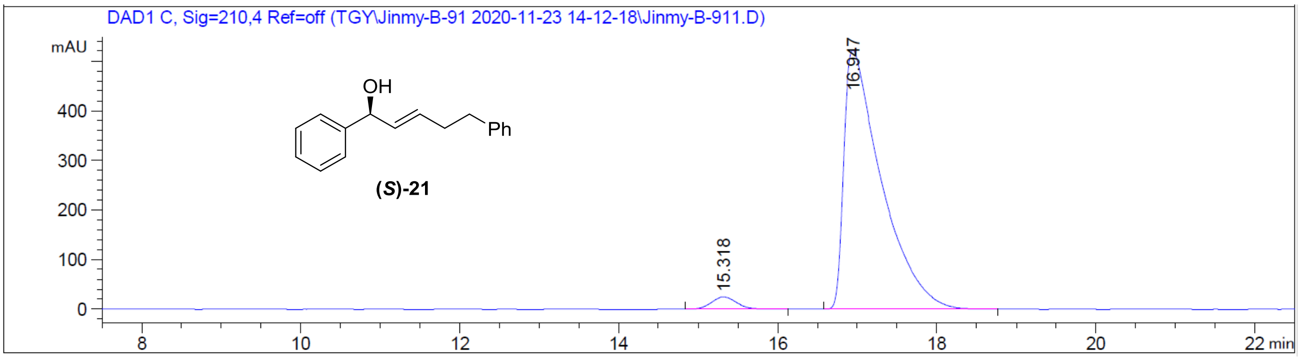

Signal 2: DAD1 C, Sig=210,4 Ref=off

Peak #	RetTime [min]	Type	Width [min]	Area [mAU*s]	Height [mAU]	Area %
1	15.318	BB	0.3385	538.80035	24.70313	3.0503
2	16.947	BB	0.4683	1.71249e4	522.60358	96.9497

5. X-Ray crystal data

Determination of the absolute configuration of (*S*)-3:

In order to obtain a high-quality crystal, the major optically pure enantiomer (100% ee) of **(*S*)-3** was purified by chiral preparative HPLC (AD-H, 2.0*25 cm, 5μm), then the pure **(*S*)-3** was further transformed to its ester derivative. The pure **(*S*)-3** (1.0 equiv), (**1*S***)-(-)-camphanic acid chloride (1.5 equiv) and DMAP (1.0 equiv) were dissolved in dichloromethane, Et_3N (1.5 equiv) was added slowly at room temperature and the reaction mixture was stirred for 12 h. The reaction mixture was quenched with saturated NH_4Cl and extracted with dichloromethane. The combined extracts were dried over Na_2SO_4. and evaporated the solvent under vaccum. The organic phase was concentrated under reduced pressure to give the crude product and purified by silica gel column chromatography (eluent : EtOAc/Hexane, 10:1) to afford **4** as a white solid.

The single crystal of the optically pure compound **4** was obtained via dichloromethane/*n*-hexane system. CCDC No. 1978582. Displacement ellipsoids are drawn at the 50% probability level.

(1*S*,4*R*)-((*S*)-1-(biphenyl-4-yl)allyl) 4,7,7-trimethyl-3-oxo-2-oxabicyclo[2.2.1]heptane-1-carboxylate.

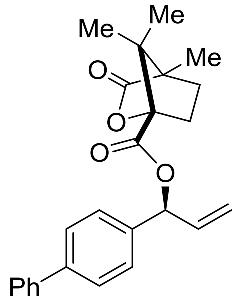

4

^{1}H NMR (400 MHz, CDCl$_3$): δ 7.56 - 7.64 (m, 4 H), 7.42 - 7.50 (m, 4H), 7.32 - 7.40 (m, 1H), 6.44 (d, *J* = 8.0 Hz, 1H), 6.10 - 6.15 (m, 1H), 5.40 (d, *J* = 16.0 Hz, 1H), 5.32 (d, *J* = 12.0 Hz, 1H), 2.38 - 2.50 (m, 1H), 1.99 - 2.11 (m, 1H), 1.87 - 1.98 (m, 1H), 1.64 - 1.75 (m, 1H), 1.61 (s, 1H), 1.11 (s, 3H), 1.04 (s, 3H), 0.94 (s, 3H) ppm.

^{13}C NMR (151 MHz, CDCl$_3$): δ 178.2, 166.6, 141.4, 140.5, 137.0, 135.4, 128.8, 127.6, 127.4, 127.1, 117.8, 91.0, 54.8, 54.3, 30.6, 28.9, 16.8, 16.7, 9.6 ppm.

HRMS (ESI) m/z: $[M + Na]^+$ Calcd for $C_{25}H_{26}O_4Na$ 416.1723; Found 413.1721.

HPLC (AD-H, 0.46*25 cm, 5μm, hexane/isopropanol = 95/5, flow = 1.0 mL/min, detection at 210 nm) retention time = 13.072 min (major) and 14.494 min (minor).

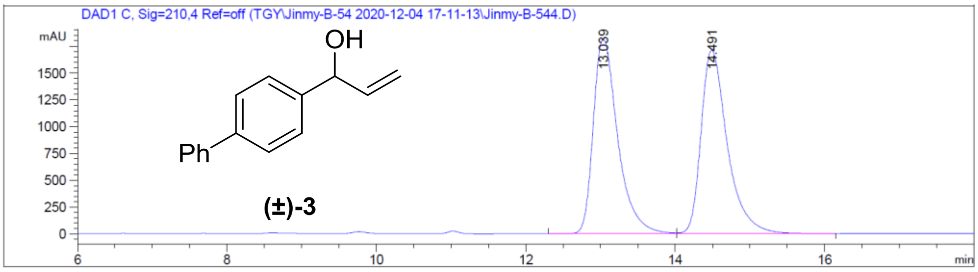

Signal 2: DAD1 C, Sig=210,4 Ref=off

Peak #	RetTime [min]	Type	Width [min]	Area [mAU*s]	Height [mAU]	Area %
1	13.039	BV	0.3480	4.14834e4	1819.43921	49.7224
2	14.491	VB	0.3718	4.19466e4	1712.21204	50.2776

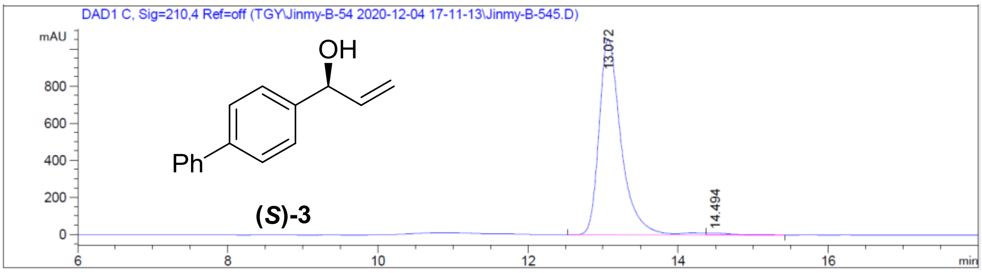

Signal 2: DAD1 C, Sig=210,4 Ref=off

Peak #	RetTime [min]	Type	Width [min]	Area [mAU*s]	Height [mAU]	Area %
1	13.072	BV R	0.3045	2.16323e4	1057.56714	99.0616
2	14.494	VB E	0.3109	204.91400	9.60375	0.9384

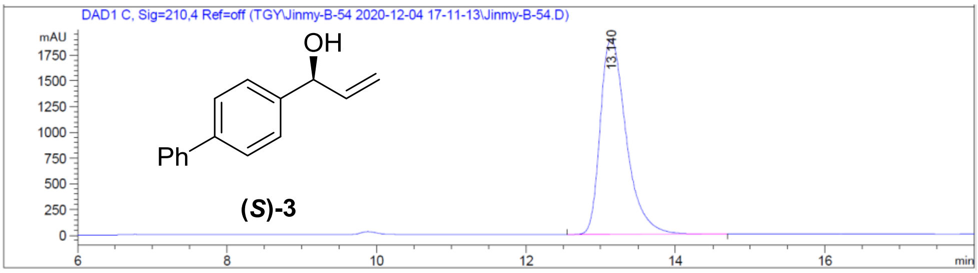

Signal 2: DAD1 C, Sig=210,4 Ref=off

Peak #	RetTime [min]	Type	Width [min]	Area [mAU*s]	Height [mAU]	Area %
1	13.140	BB	0.3745	4.58514e4	1893.30969	100.0000

HPLC (AD-H, 0.46*25 cm, 5μm, hexane / ethanol = 90/10, flow = 1.0 mL/min, detection at 254 nm) retention time = 14.523 min.

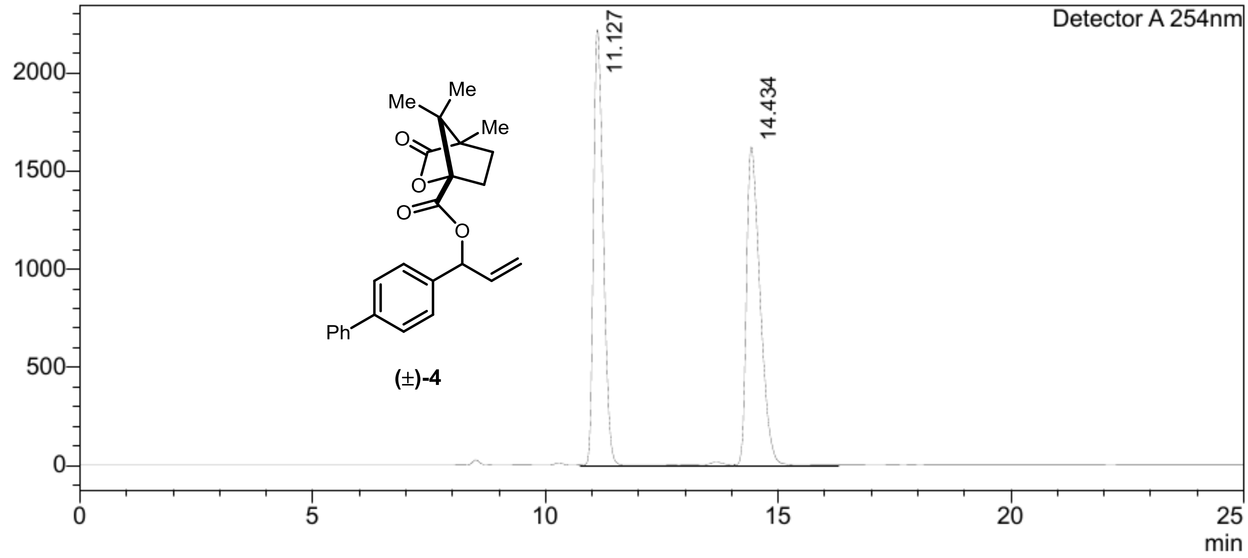

<Peak Table>

Detector A 254nm

Peak#	Ret. Time	Area	Height	Conc.	Unit	Mark	Name
1	11.127	32671432	2217599	49.894			
2	14.434	32810149	1620406	50.106		V	
Total		65481581	3838004				

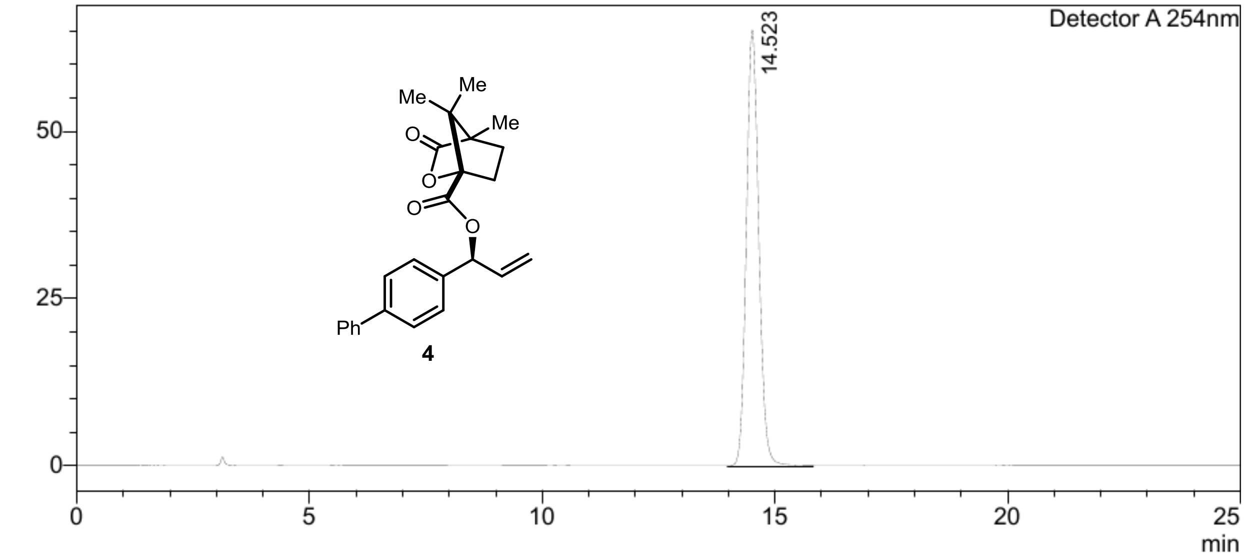

<Peak Table>

Detector A 254nm

Peak#	Ret. Time	Area	Height	Conc.	Unit	Mark	Name
1	14.523	1205421	65196	100.000			
Total		1205421	65196				

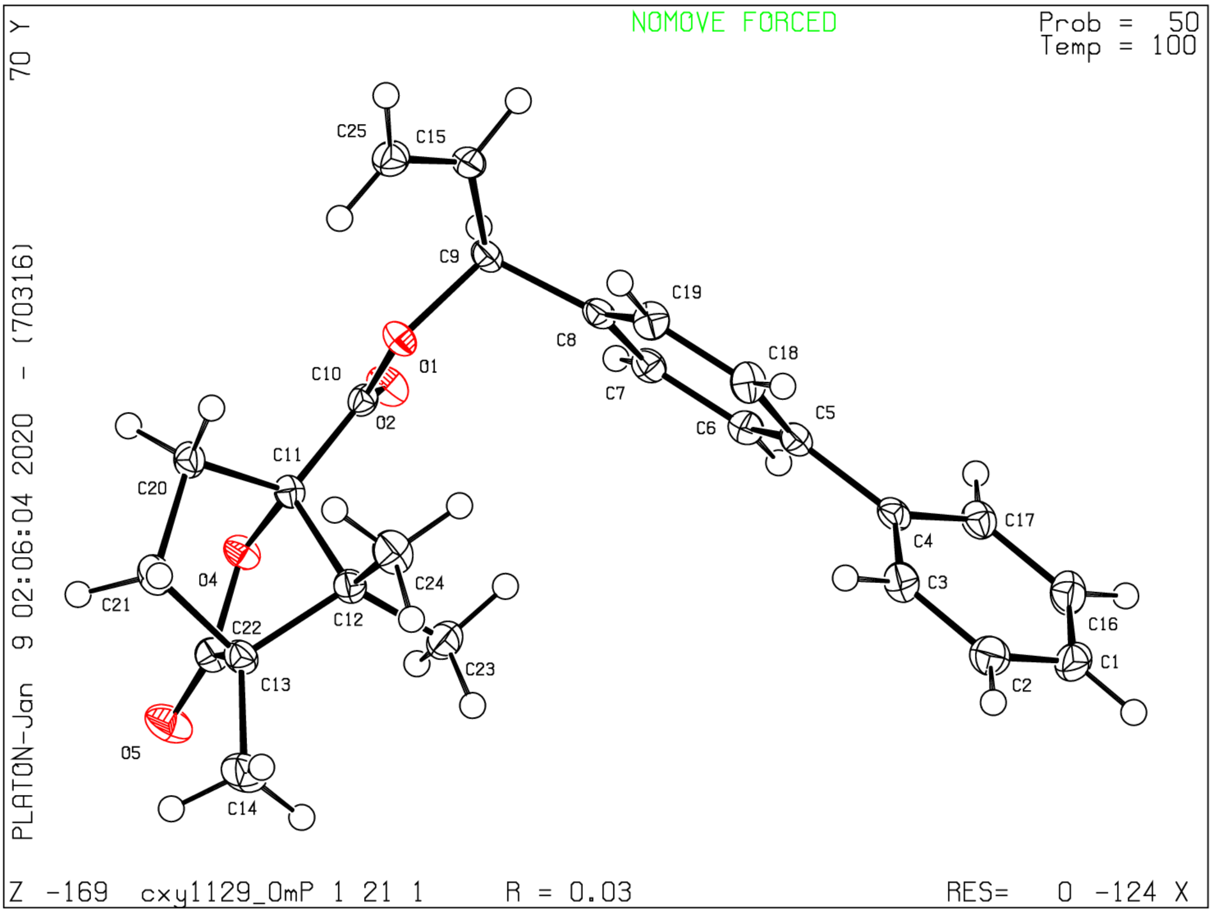

Fig S1. Crystal data and structure refinement for **4**.

Table S1. Crystal data and structure refinement for complex **4**

CCDC Number	1978582
Empirical formula	$C_{25}H_{26}O_4$
Formula weight	390.46
Temperature/K	100
Crystal system	monoclinic
Space group	$P2_1$
a/Å	6.1746(3)
b/Å	23.4483(12)
c/Å	7.3185(4)
α/°	90
β/°	104.2282(2)
γ/°	90
Volume/Å^3	1027.10(9)
Z	2
ρ_{calc}g/cm^3	1.263
μ/mm^{-1}	0.678
F(000)	416.0
Crystal size/mm^3	0.41 × 0.38 × 0.09
Radiation	CuKα (λ = 1.54178)
2Θ range for data collection/°	7.54 to 136.974
Index ranges	-7 ≤ h ≤ 7, -28 ≤ k ≤ 28, -8 ≤ l ≤ 8
Reflections collected	16222
Independent reflections	3743 [R_{int} = 0.0394, R_{sigma} = 0.0326]
Data/restraints/parameters	3743/1/266
Goodness-of-fit on F^2	1.044
Final R indexes [I>=2σ (I)]	R_1 = 0.0252, wR_2 = 0.0640
Final R indexes [all data]	R_1 = 0.0254, wR_2 = 0.0642
Largest diff. peak/hole / e Å^{-3}	0.16/-0.14
Flack parameter	0.10(7)

6. References

1. Chen, F.; He, D.; Chen, L.; Chang, X.Y.; Wang, D.Z.; Xu, C.; Xing, X. Chirality-Economy Catalysis: Asymmetric Transfer Hydrogenation of Ketones by Ru-Catalysts of Minimal Stereogenicity. *ACS Catal*. **2019**, *9*, 5562–5566.

2. Pan, Y.; You,Y.; He, D.; Chen. F.; Chang, X.; Jin, M.Y.; Xing, X. Asymmetric Synthesis of γ-Secondary Amino Alcohols via a Borrowing-Hydrogen Cascade. *Org. Lett*. **2020**, *22*, 7278–7283.

3. He, D.; Xu, X.; Lu, Y.; Zhou, M.J.; Xiang, X. Asymmetric Transfer Hydrogenation of Densely Functionalized Diheteroaryl and Diaryl Ketones by a Ru-Catalyst of Minimal Stereogenicity. *Org. Lett*. **2020**, *22*, 8458–8463.

4. Jin, M.Y.; Zhou, Y.; Xiao, D.; You, Y.; Zhen, Q.; Tao, G.; Yu, P.; Xing, X. Simultaneous Kinetic Resolution and Asymmetric Induction within a Borrowing Hydrogen Cascade Mediated by a Single Catalyst. *Angew. Chem. Int. Ed. Engl*. **2021**, DOI: 10.1002/anie.202112993.

5. Trost, B. M., Kalnmals, C. A., Ramakrishnan, D., Ryan, M. C., Smaha, R. W. & Parkin, S. Ruthenium-Catalyzed Asymmetric Allylic Alkylation of Isatins. *Org. Lett*. **2020**, *22*, 2584–2589.

7. NMR

Parameter	Value
1 Title	lcc-S-011-H1.1.1.1r
2 Comment	
3 Origin	Bruker BioSpin GmbH
4 Owner	nmrsu
5 Instrument	AVANCE NEO 400 MHZ DIGITAL NMR SPECTROMETER
6 Author	
7 Solvent	CDCl3
8 Temperature	296.3
9 Pulse Sequence	zg30
10 Experiment	1D
11 Probe	Z116098_0723 (PA BBO 400S1 BBF-H-D-05 Z SP)
12 Number of Scans	8
13 Receiver Gain	101.0
14 Relaxation Delay	1.0000
15 Pulse Width	10.0000
16 Acquisition Time	3.9977
17 Acquisition Date	2020-04-14T02:09:58
18 Modification Date	2020-04-14T09:20:38
19 Spectrometer Frequency	400.13
20 Spectral Width	8196.7
21 Lowest Frequency	-1637.1
22 Nucleus	1H
23 Acquired Size	32768
24 Spectral Size	65536

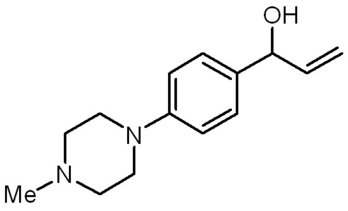

(±)-11

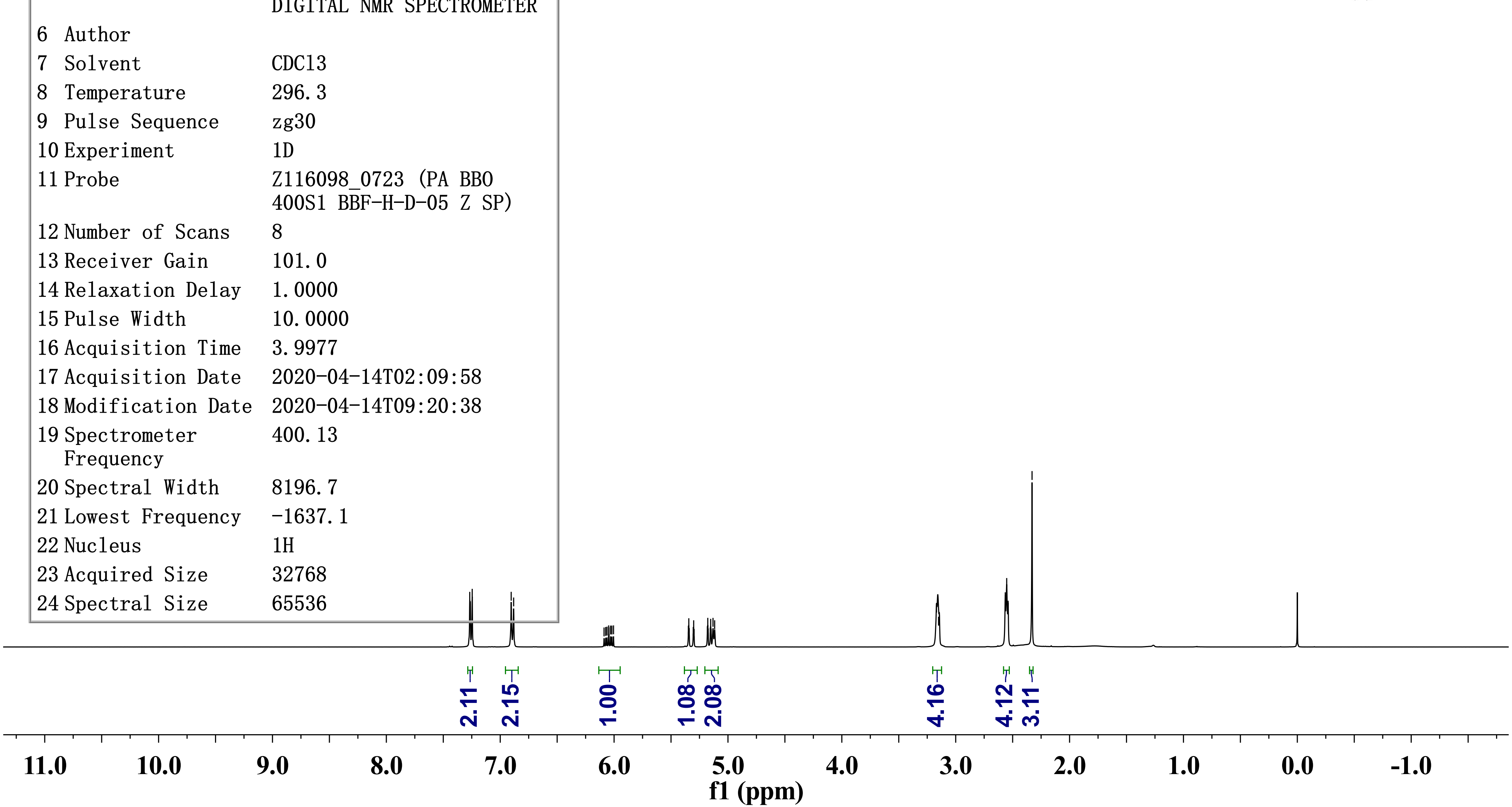

	Parameter	Value
1	Title	lcc-S-011-C13.3.1.1r
2	Comment	
3	Origin	Bruker BioSpin GmbH
4	Owner	nmrsu
5	Instrument	AVANCE NEO 400 MHZ DIGITAL NMR SPECTROMETER
6	Author	
7	Solvent	CDCl3
8	Temperature	296.2
9	Pulse Sequence	zgpg30
10	Experiment	1D
11	Probe	Z116098_0723 (PA BBO 400S1 BBF-H-D-05 Z SP)
12	Number of Scans	1024
13	Receiver Gain	71.5
14	Relaxation Delay	2.0000
15	Pulse Width	10.0000
16	Acquisition Time	1.3763
17	Acquisition Date	2020-04-14T03:10:09
18	Modification Date	2020-04-14T09:20:38
19	Spectrometer Frequency	100.61
20	Spectral Width	23809.5
21	Lowest Frequency	-1845.6
22	Nucleus	13C
23	Acquired Size	32768
24	Spectral Size	32768

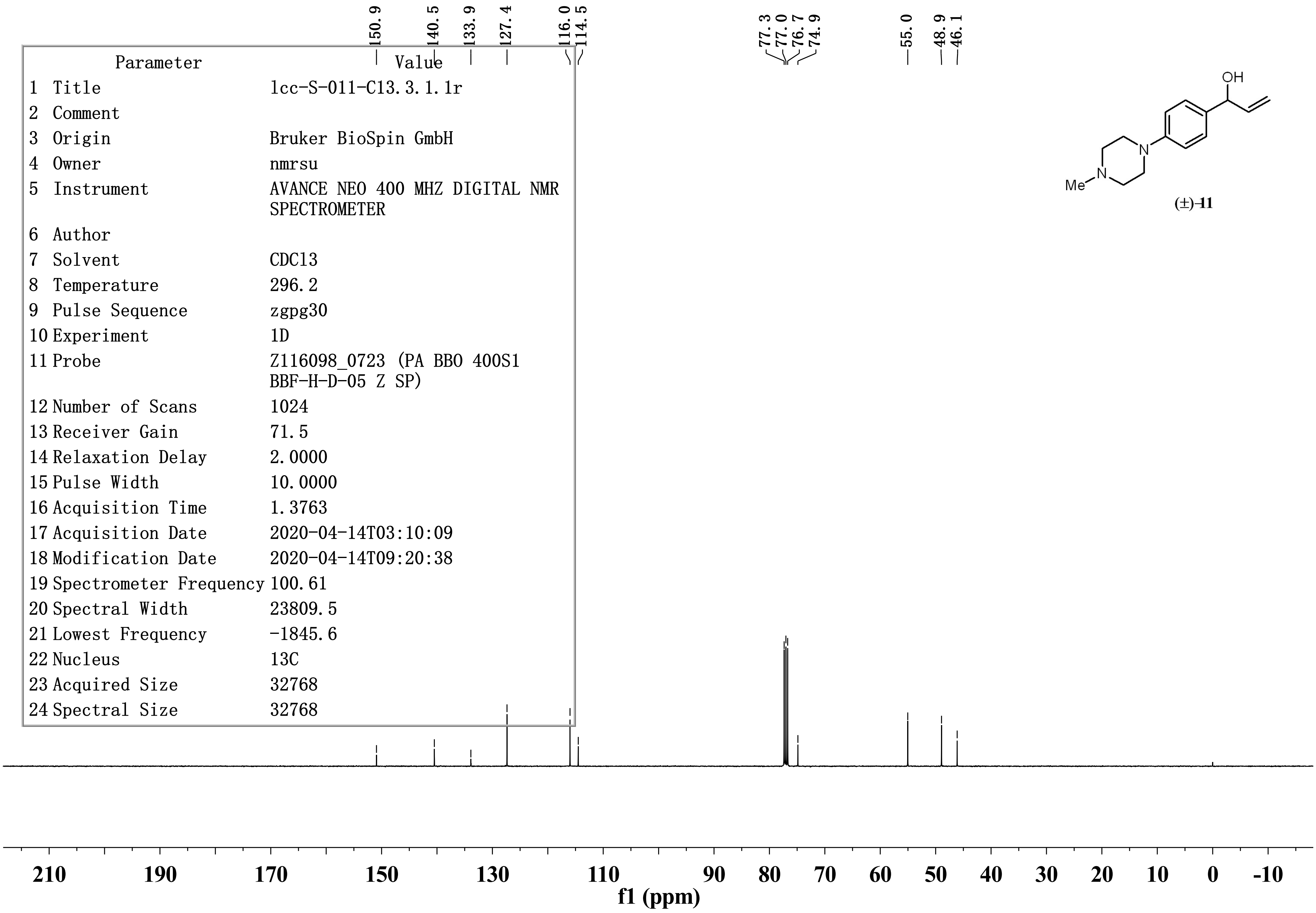

(±)-11

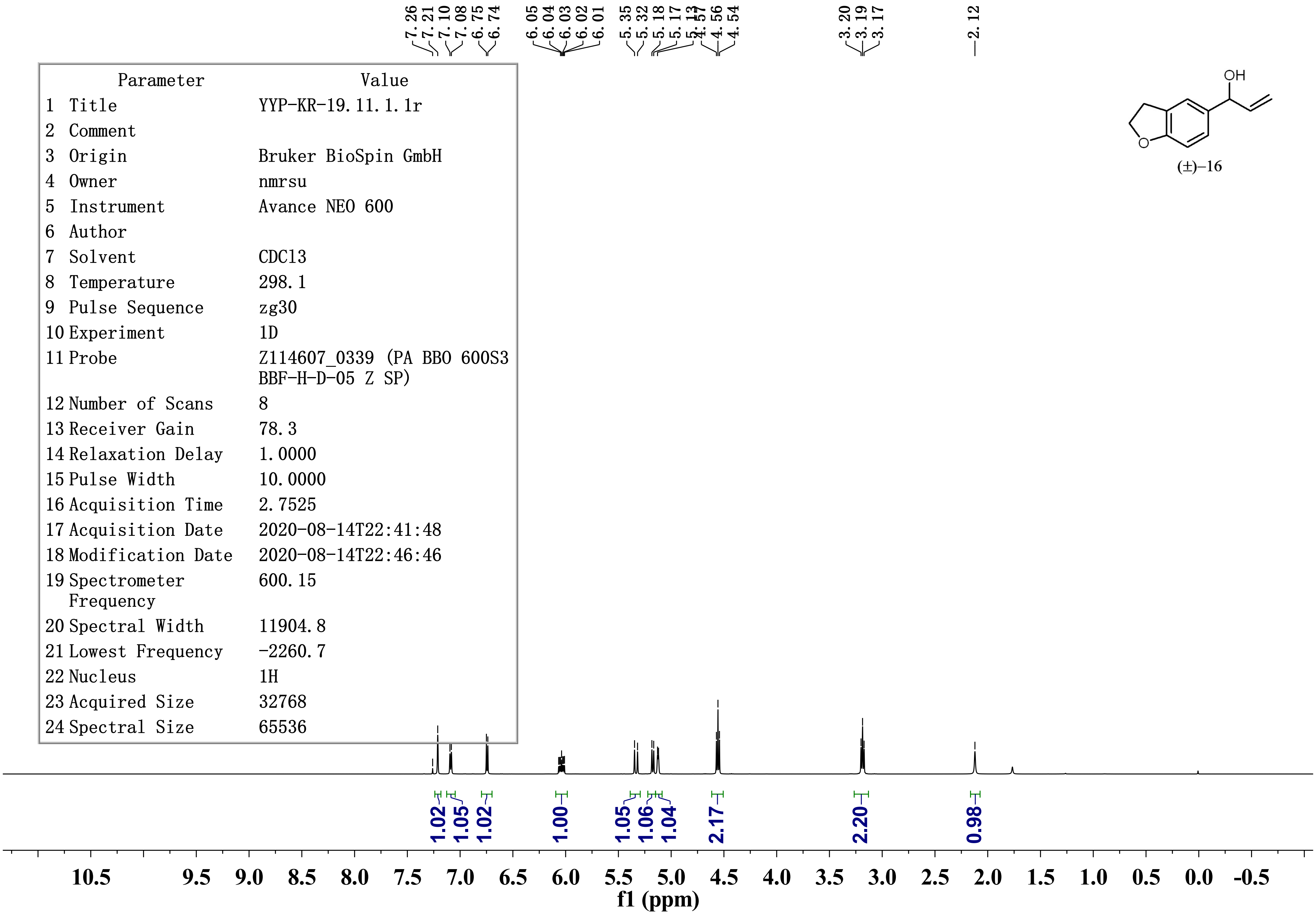

	Parameter	Value
1	Title	YYP-KR-19.11.1.1r
2	Comment	
3	Origin	Bruker BioSpin GmbH
4	Owner	nmrsu
5	Instrument	Avance NEO 600
6	Author	
7	Solvent	CDCl3
8	Temperature	298.1
9	Pulse Sequence	zg30
10	Experiment	1D
11	Probe	Z114607_0339 (PA BBO 600S3 BBF-H-D-05 Z SP)
12	Number of Scans	8
13	Receiver Gain	78.3
14	Relaxation Delay	1.0000
15	Pulse Width	10.0000
16	Acquisition Time	2.7525
17	Acquisition Date	2020-08-14T22:41:48
18	Modification Date	2020-08-14T22:46:46
19	Spectrometer Frequency	600.15
20	Spectral Width	11904.8
21	Lowest Frequency	-2260.7
22	Nucleus	1H
23	Acquired Size	32768
24	Spectral Size	65536

(±)-16

	Parameter	Value
1	Title	lcc-S-016-C13.10.1.1r
2	Comment	
3	Origin	Bruker BioSpin GmbH
4	Owner	nmrsu
5	Instrument	Avance NEO 600
6	Author	
7	Solvent	CDCl3
8	Temperature	296.8
9	Pulse Sequence	zgpg30
10	Experiment	1D
11	Probe	Z114607_0339 (PA BBO 600S3 BBF-H-D-05 Z SP)
12	Number of Scans	40
13	Receiver Gain	101.0
14	Relaxation Delay	2.0000
15	Pulse Width	12.0000
16	Acquisition Time	0.9175
17	Acquisition Date	2020-04-15T23:07:59
18	Modification Date	2020-04-15T23:20:18
19	Spectrometer Frequency	150.91
20	Spectral Width	35714.3
21	Lowest Frequency	-2777.2
22	Nucleus	13C
23	Acquired Size	32768
24	Spectral Size	32768

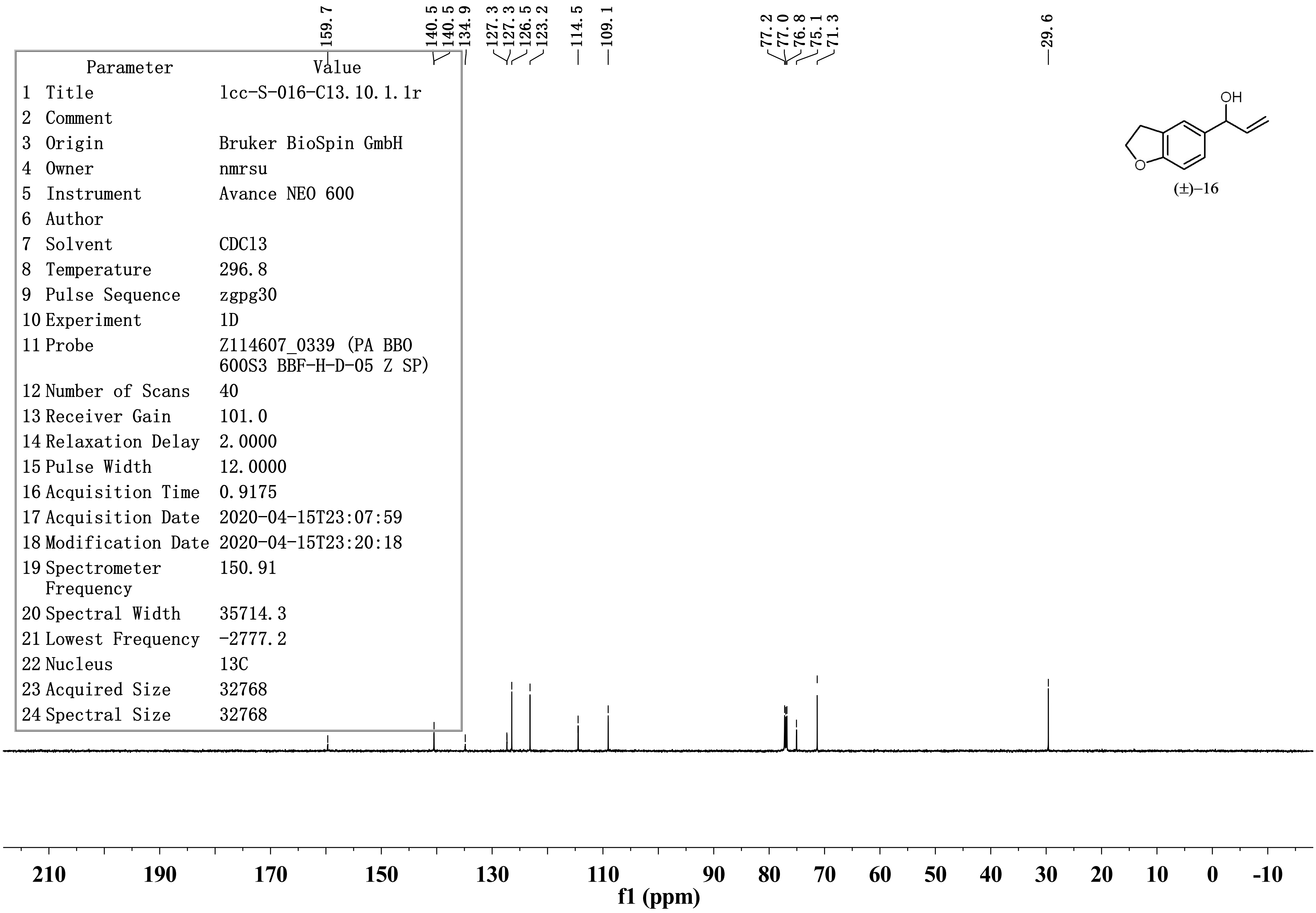

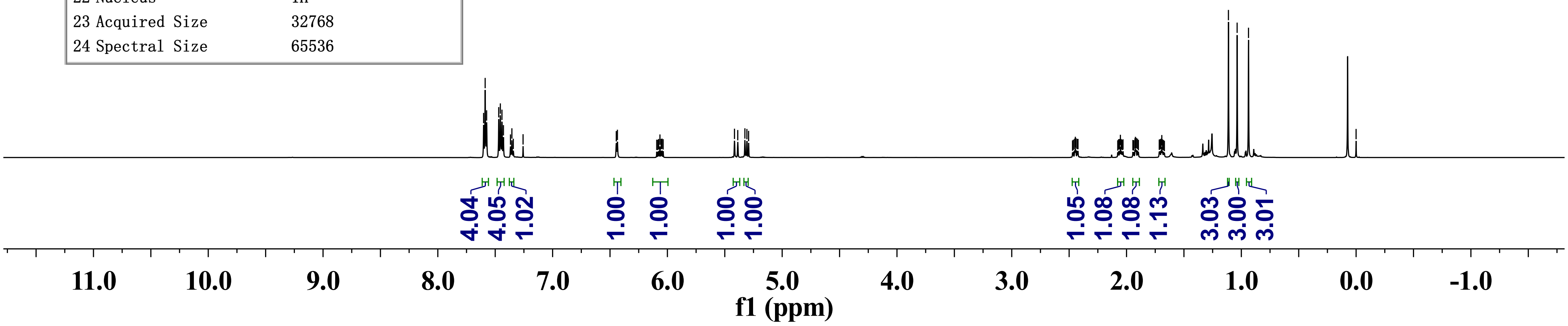

	Parameter	Value
1	Title	hdx-4-90-chiral
2	Comment	
3	Origin	Bruker BioSpin GmbH
4	Owner	nmrsu
5	Site	
6	Spectrometer	Avance NEO 600
7	Author	
8	Solvent	CDCl3
9	Temperature	296.6
10	Pulse Sequence	zg30
11	Experiment	1D
12	Number of Scans	16
13	Receiver Gain	87
14	Relaxation Delay	1.0000
15	Pulse Width	10.0000
16	Acquisition Time	2.7525
17	Acquisition Date	2020-01-04T22:19:29
18	Modification Date	2020-11-24T16:31:36
19	Spectrometer Frequency	600.15
20	Spectral Width	11904.8
21	Lowest Frequency	-2262.7
22	Nucleus	1H
23	Acquired Size	32768
24	Spectral Size	65536

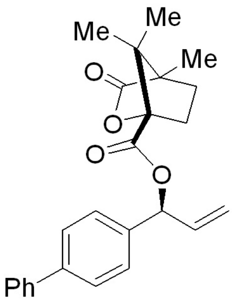

4

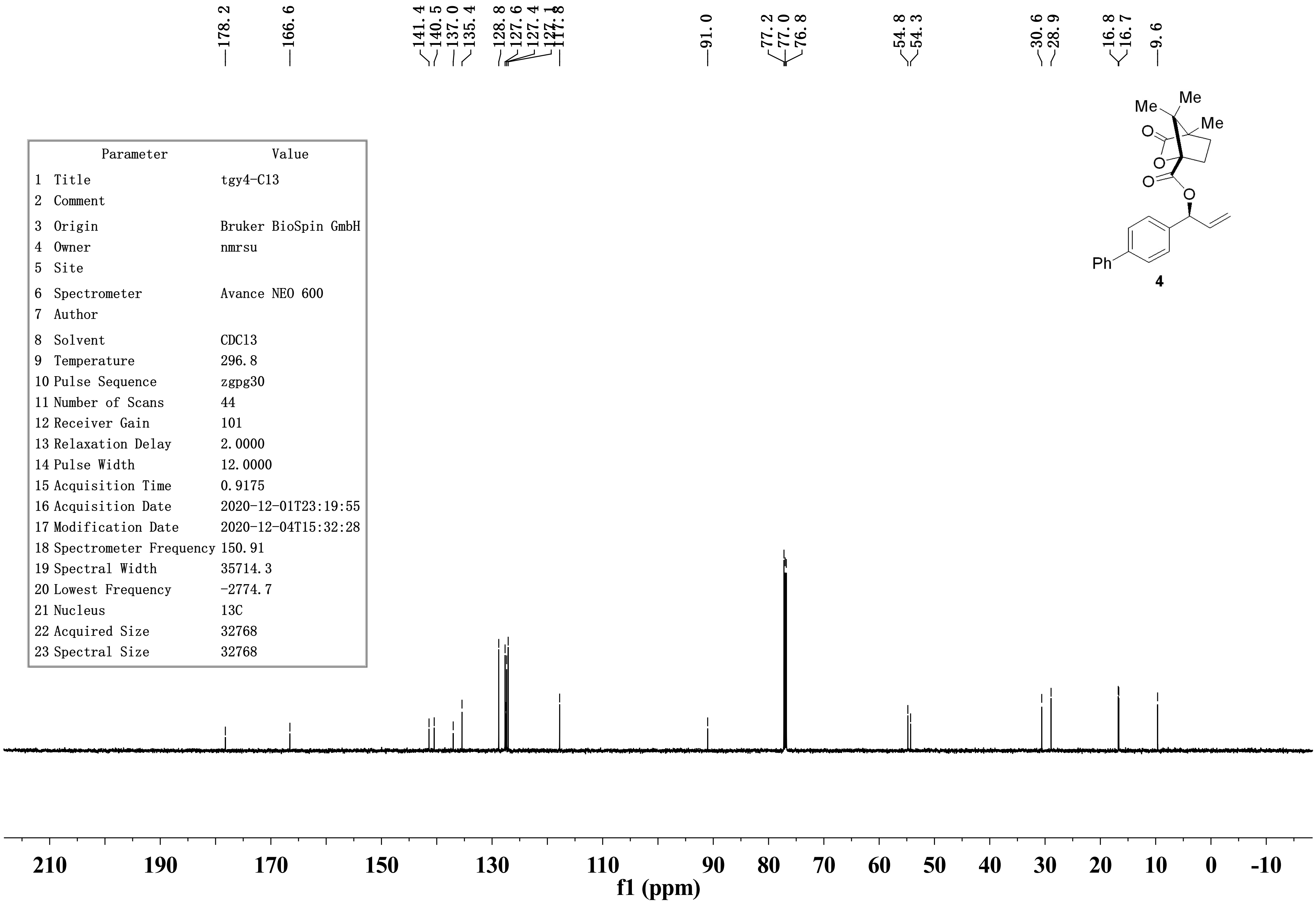

4

	Parameter	Value
1	Title	tgy4-C13
2	Comment	
3	Origin	Bruker BioSpin GmbH
4	Owner	nmrsu
5	Site	
6	Spectrometer	Avance NEO 600
7	Author	
8	Solvent	CDCl3
9	Temperature	296.8
10	Pulse Sequence	zgpg30
11	Number of Scans	44
12	Receiver Gain	101
13	Relaxation Delay	2.0000
14	Pulse Width	12.0000
15	Acquisition Time	0.9175
16	Acquisition Date	2020-12-01T23:19:55
17	Modification Date	2020-12-04T15:32:28
18	Spectrometer Frequency	150.91
19	Spectral Width	35714.3
20	Lowest Frequency	-2774.7
21	Nucleus	13C
22	Acquired Size	32768
23	Spectral Size	32768

Parameter	Value
1 Title	20200313-1-Ph-1-propanol.10.1.1r
2 Comment	
3 Origin	Bruker BioSpin GmbH
4 Owner	nmrsu
5 Site	
6 Instrument	Avance NEO 600
7 Author	
8 Solvent	CDCl3
9 Temperature	296.1
10 Pulse Sequence	zg30
11 Experiment	1D
12 Probe	Z114607_0339 (PA BBO 600S3 BBF-H-D-05 Z SP)
13 Number of Scans	8
14 Receiver Gain	59.8
15 Relaxation Delay	1.0000
16 Pulse Width	10.0000
17 Presaturation Frequency	
18 Acquisition Time	2.7525
19 Acquisition Date	2020-03-13T14:40:29
20 Modification Date	2020-03-14T09:19:35
21 Class	
22 Spectrometer Frequency	600.15
23 Spectral Width	11904.8
24 Lowest Frequency	-2272.3
25 Nucleus	1H
26 Acquired Size	32768
27 Spectral Size	65536

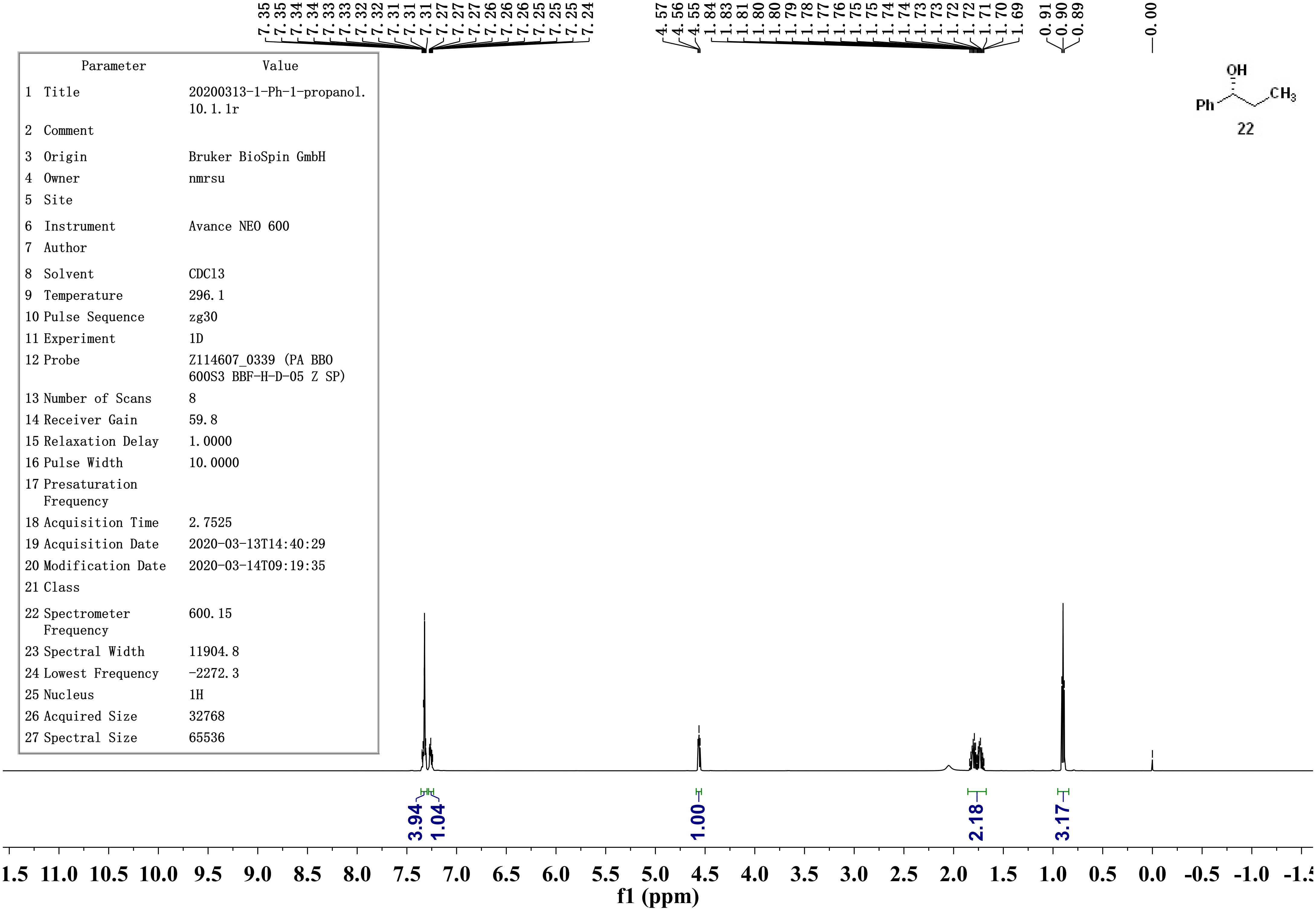

22

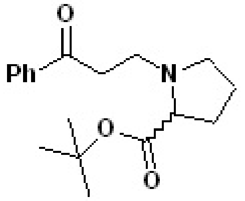

23

Parameter	Value
1 Comment	
2 Origin	Bruker BioSpin GmbH
3 Owner	nmrsu
4 Site	
5 Spectrometer	Avance NEO 600
6 Author	
7 Solvent	CDCl3
8 Temperature	298.2
9 Pulse Sequence	zg30
10 Number of Scans	8
11 Receiver Gain	101
12 Relaxation Delay	1.0000
13 Pulse Width	11.1300
14 Acquisition Time	2.7525
15 Acquisition Date	2021-12-08T15:16:45
16 Modification Date	2021-12-08T15:29:07
17 Spectrometer Frequency	600.15
18 Spectral Width	11904.8
19 Lowest Frequency	-2258.8
20 Nucleus	1H
21 Acquired Size	32768
22 Spectral Size	65536

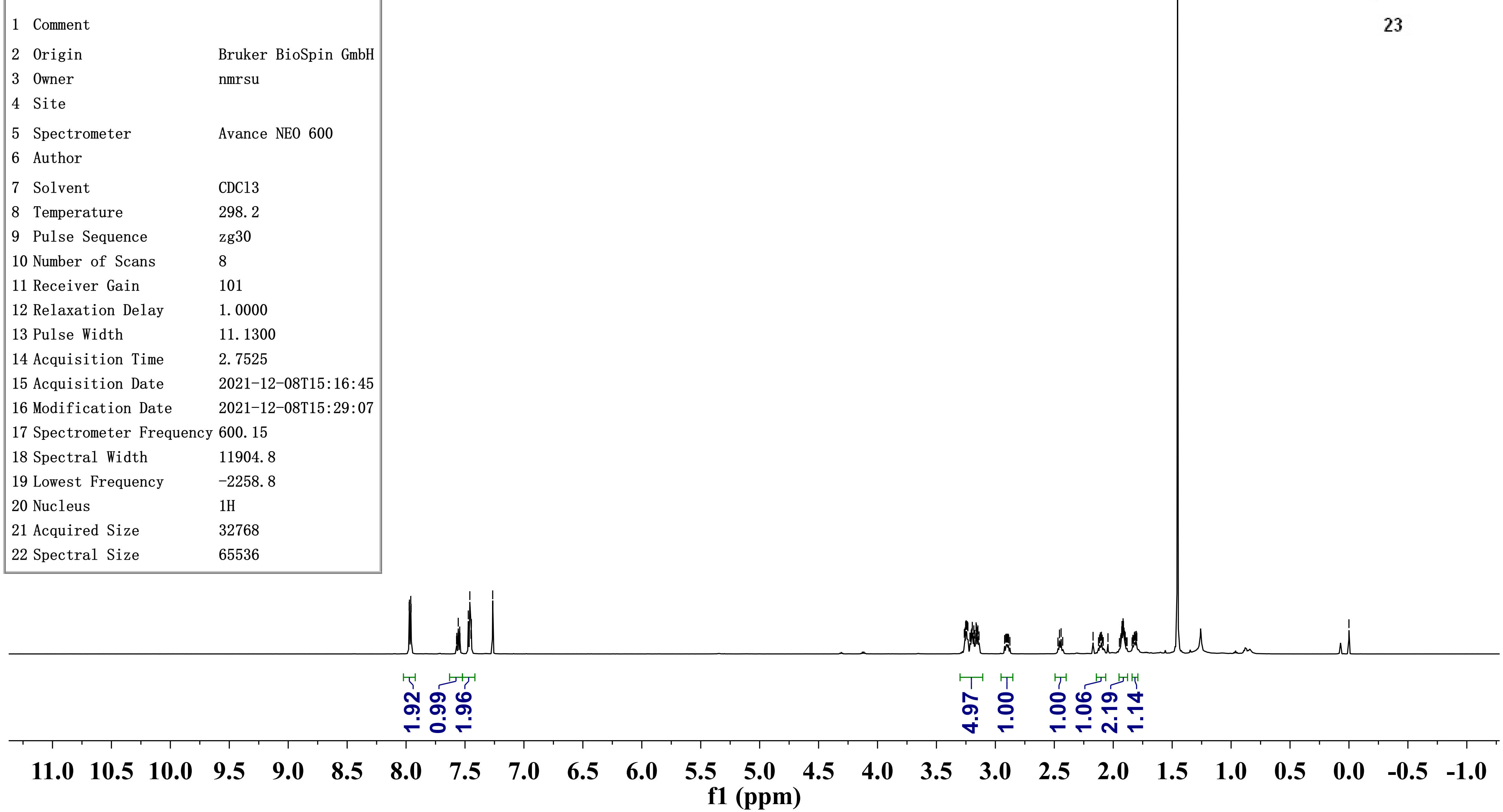

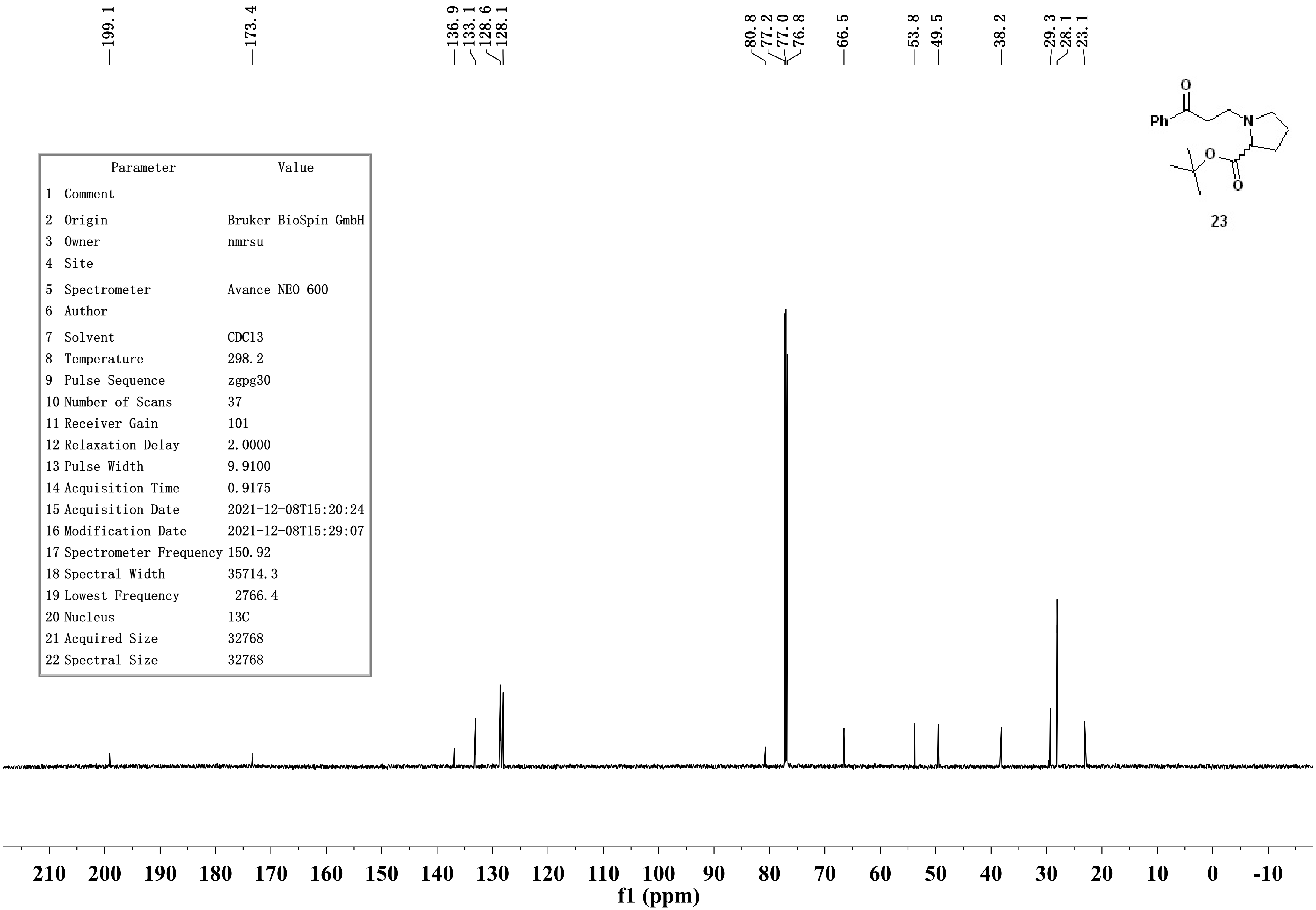

	Parameter	Value
1	Comment	
2	Origin	Bruker BioSpin GmbH
3	Owner	nmrsu
4	Site	
5	Spectrometer	Avance NEO 600
6	Author	
7	Solvent	CDCl3
8	Temperature	298.2
9	Pulse Sequence	zgpg30
10	Number of Scans	37
11	Receiver Gain	101
12	Relaxation Delay	2.0000
13	Pulse Width	9.9100
14	Acquisition Time	0.9175
15	Acquisition Date	2021-12-08T15:20:24
16	Modification Date	2021-12-08T15:29:07
17	Spectrometer Frequency	150.92
18	Spectral Width	35714.3
19	Lowest Frequency	-2766.4
20	Nucleus	13C
21	Acquired Size	32768
22	Spectral Size	32768

23

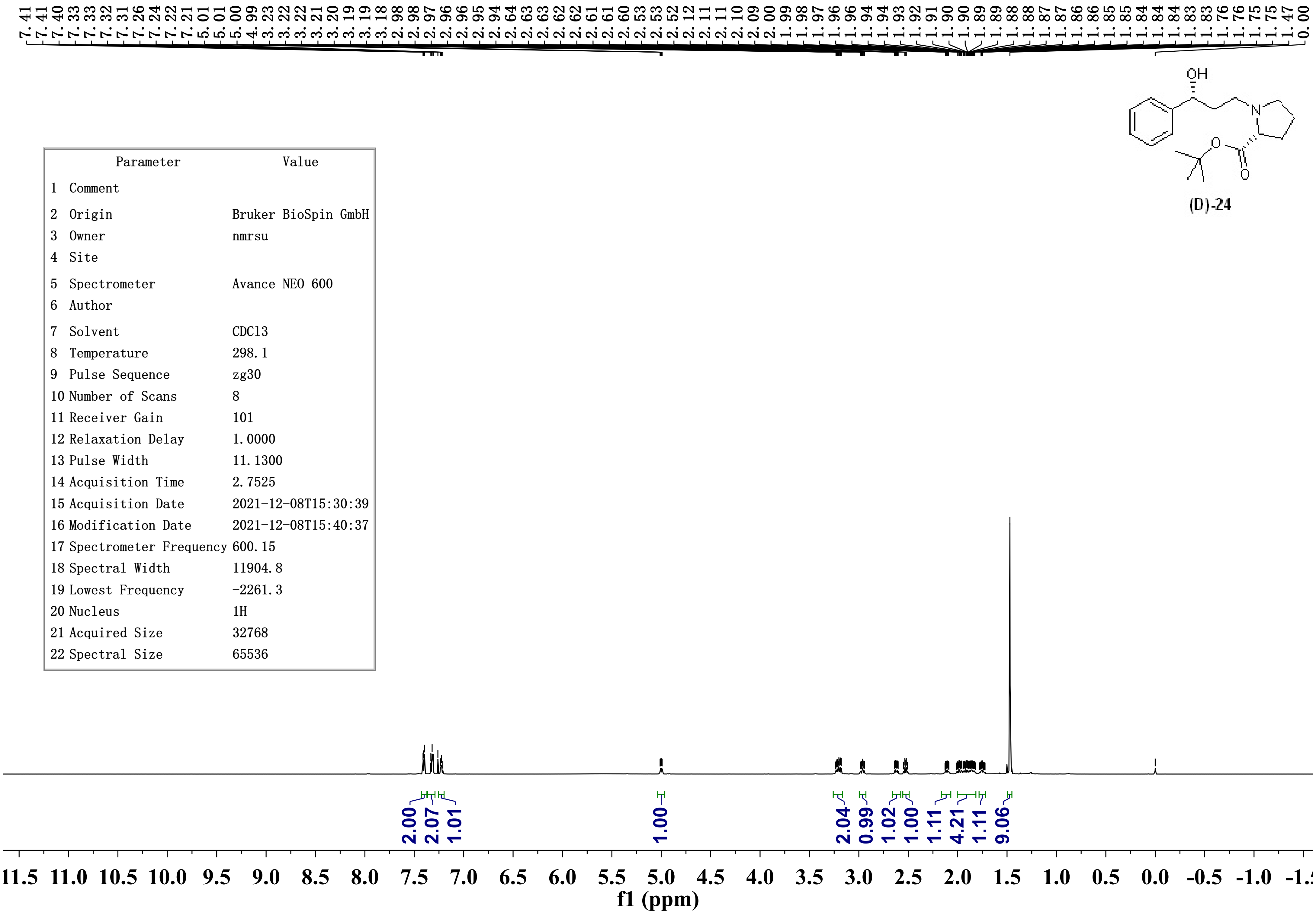

Parameter	Value
1 Comment	
2 Origin	Bruker BioSpin GmbH
3 Owner	nmrsu
4 Site	
5 Spectrometer	Avance NEO 600
6 Author	
7 Solvent	CDCl3
8 Temperature	298.1
9 Pulse Sequence	zg30
10 Number of Scans	8
11 Receiver Gain	101
12 Relaxation Delay	1.0000
13 Pulse Width	11.1300
14 Acquisition Time	2.7525
15 Acquisition Date	2021-12-08T15:30:39
16 Modification Date	2021-12-08T15:40:37
17 Spectrometer Frequency	600.15
18 Spectral Width	11904.8
19 Lowest Frequency	-2261.3
20 Nucleus	1H
21 Acquired Size	32768
22 Spectral Size	65536

(D)-24

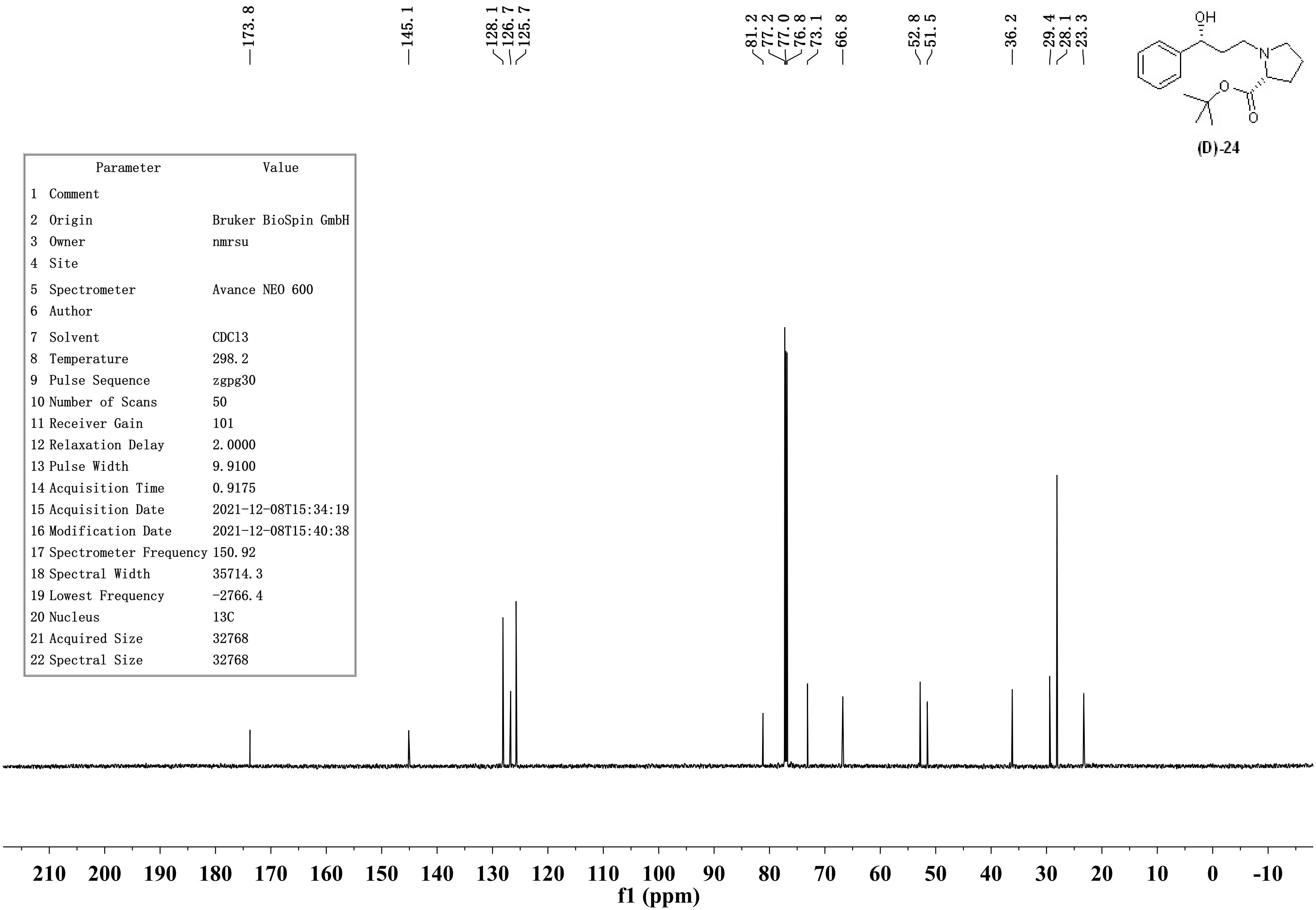

173.8
145.1
128.1
126.7
125.7
81.2
77.2
77.0
76.8
73.1
66.8
52.8
51.5
36.2
29.4
28.1
23.3
OH
N
O
O
(D)-24
Parameter Value
1 Comment
2 Origin Bruker BioSpin GmbH
3 Owner nmrsu
4 Site
5 Spectrometer Avance NEO 600
6 Author
7 Solvent CDCl3
8 Temperature 298.2
9 Pulse Sequence zgpg30
10 Number of Scans 50
11 Receiver Gain 101
12 Relaxation Delay 2.0000
13 Pulse Width 9.9100
14 Acquisition Time 0.9175
15 Acquisition Date 2021-12-08T15:34:19
16 Modification Date 2021-12-08T15:40:38
17 Spectrometer Frequency 150.92
18 Spectral Width 35714.3
19 Lowest Frequency -2766.4
20 Nucleus 13C
21 Acquired Size 32768
22 Spectral Size 32768
210 200 190 180 170 160 150 140 130 120 110 100 90 80 70 60 50 40 30 20 10 0 -10
f1 (ppm)

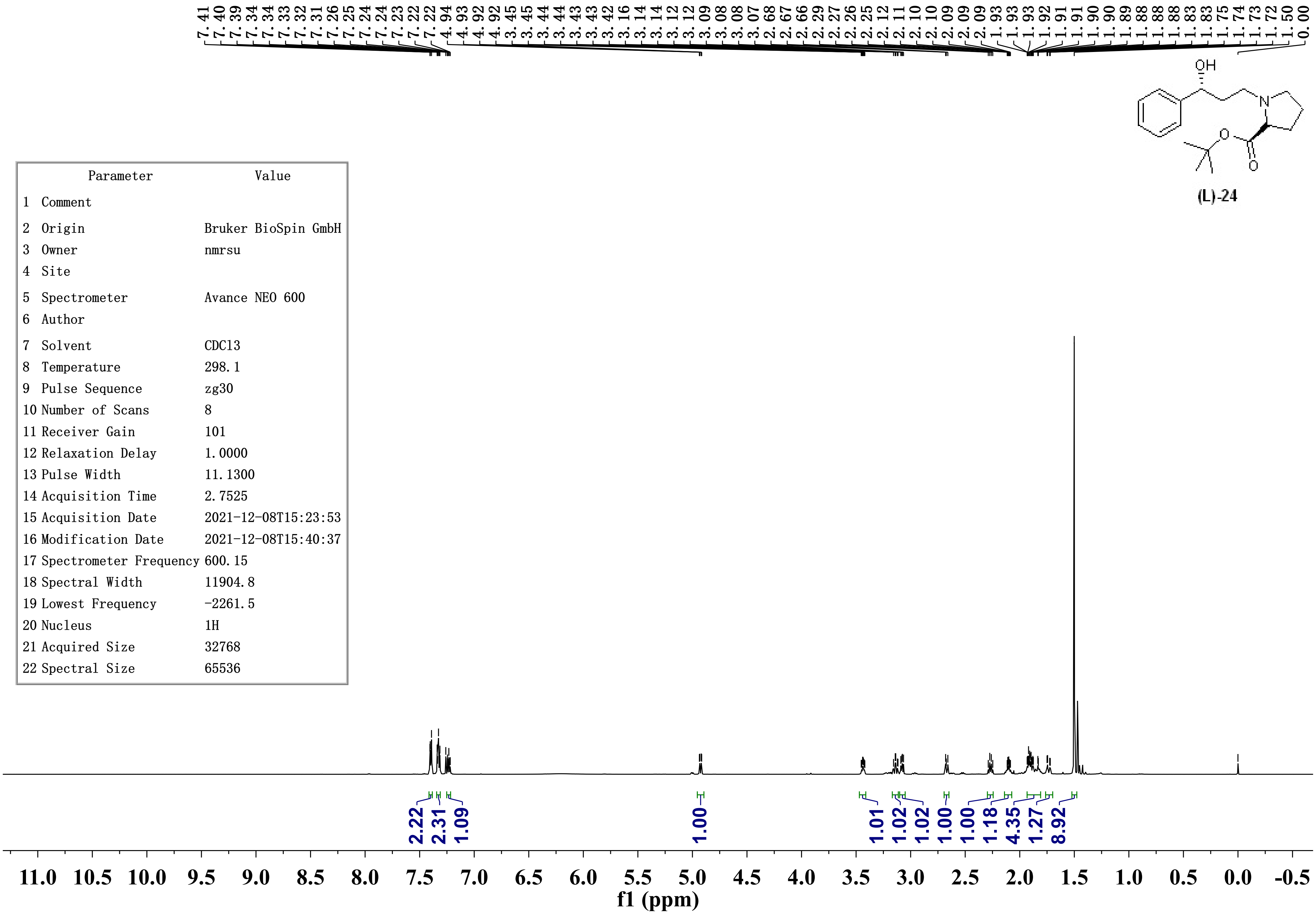

(L)-24

	Parameter	Value
1	Comment	
2	Origin	Bruker BioSpin GmbH
3	Owner	nmrsu
4	Site	
5	Spectrometer	Avance NEO 600
6	Author	
7	Solvent	CDCl3
8	Temperature	298.1
9	Pulse Sequence	zg30
10	Number of Scans	8
11	Receiver Gain	101
12	Relaxation Delay	1.0000
13	Pulse Width	11.1300
14	Acquisition Time	2.7525
15	Acquisition Date	2021-12-08T15:23:53
16	Modification Date	2021-12-08T15:40:37
17	Spectrometer Frequency	600.15
18	Spectral Width	11904.8
19	Lowest Frequency	-2261.5
20	Nucleus	1H
21	Acquired Size	32768
22	Spectral Size	65536

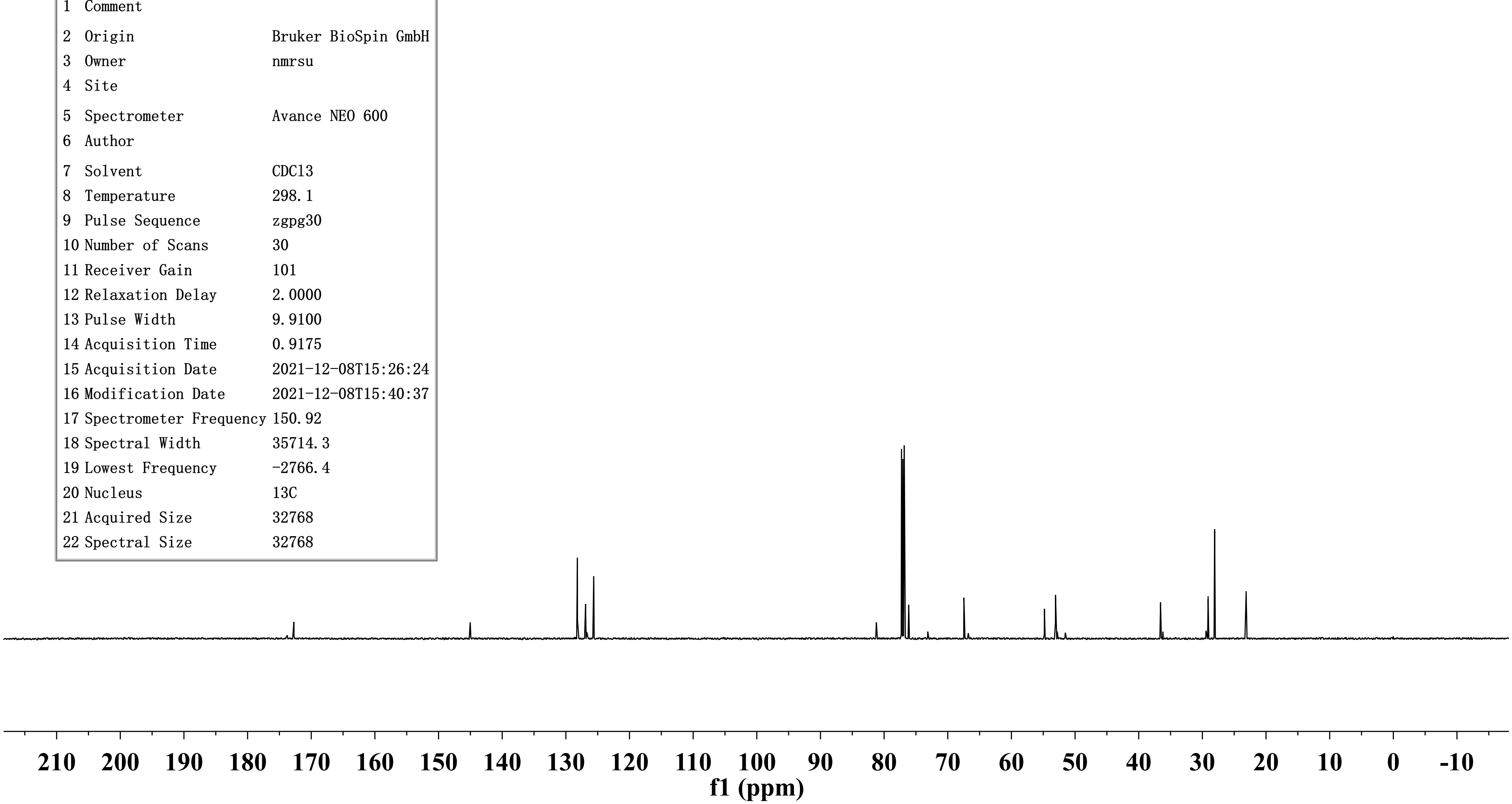

	Parameter	Value
1	Comment	
2	Origin	Bruker BioSpin GmbH
3	Owner	nmrsu
4	Site	
5	Spectrometer	Avance NEO 600
6	Author	
7	Solvent	CDCl3
8	Temperature	298.1
9	Pulse Sequence	zgpg30
10	Number of Scans	30
11	Receiver Gain	101
12	Relaxation Delay	2.0000
13	Pulse Width	9.9100
14	Acquisition Time	0.9175
15	Acquisition Date	2021-12-08T15:26:24
16	Modification Date	2021-12-08T15:40:37
17	Spectrometer Frequency	150.92
18	Spectral Width	35714.3
19	Lowest Frequency	-2766.4
20	Nucleus	13C
21	Acquired Size	32768
22	Spectral Size	32768

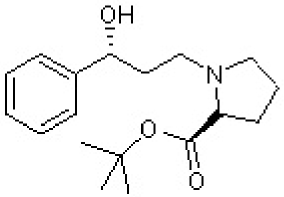

(L)-24
